# Microbiota-derived metabolite landscapes modulate *Fusobacterium* fitness and colorectal cancer cell behaviour

**DOI:** 10.1186/s13099-026-00859-9

**Published:** 2026-07-16

**Authors:** Chhavi Dhiman, Abhiram Kumar, Shreya S. Sonak, Priyanka Erukulla, Vijaykumar Dayaram Nimbarte, Kumar Pranav Narayan

**Affiliations:** 1 Department of Biological Sciences, Birla Institute of Technology and Science (BITS) - Pilani, Hyderabad campus, Jawahar Nagar, Hyderabad, 500078 India; 2 Department of Pharmacy, Birla Institute of Technology and Science (BITS) - Pilani, Hyderabad campus, Jawahar Nagar, Hyderabad, 500078 India

**Keywords:** Gut microbiota, Dysbiosis, Colorectal cancer, *Fusobacterium*, Oral microbiota, Host-microbiota interactions

## Abstract

**Background:**

Gut microbiota dysbiosis is increasingly viewed as a disruption of microbial metabolic functions rather than only a shift in microbial composition. Microbiota-derived metabolites not only shape microbial ecology but also directly influence surrounding host tissues by modulating epithelial cell signaling, inflammation, and tumor-associated processes. Among dysbiosis-associated microbes, *Fusobacterium* is consistently enriched in colorectal cancer (CRC) and contributes to tumor progression, yet the ecological factors regulating its expansion and interaction with host tissues remain unclear. Here, we investigated how microbiome-derived metabolite environments associated with healthy and dysbiotic microbial communities influence *Fusobacterium* fitness and colorectal epithelial cell behavior.

**Methods:**

CRC-associated dysbiosis was generated using an orthotopic murine CRC model combined with antibiotic-induced microbiota perturbation. Gut microbial communities were profiled using 16 S rRNA gene sequencing. Metabolite-enriched supernatants derived from healthy gut microbiota, oral microbiota, dysbiotic microbiota and probiotic cultures were evaluated for their effects on CRC-associated bacteria and *Fusobacterium* sp. growth, adhesion and invasion. These metabolite supernatants were applied to colorectal cancer cells and their effects on viability (MTT assay), migration (scratch assay), apoptosis (Annexin V-FITC flow cytometry), and inflammatory signaling (Western blot analysis of inflammatory markers) were evaluated.

**Results:**

CRC-associated dysbiosis showed reduced microbial diversity with enrichment of opportunistic taxa including *Fusobacterium* and depletion of beneficial commensals such as *Lactobacillus* and *Bifidobacterium*. Metabolite-enriched supernatants from healthy gut and oral microbiota suppressed *Fusobacterium* growth by 55–65% and reduced bacterial adhesion and invasion in epithelial cells. In epithelial models, these metabolite environments reduced CRC viability to 60% of untreated control, with comparatively smaller effects observed in non-cancerous epithelial cells. They also inhibited cell migration, accompanied by suppression of inflammatory signaling pathways including IL-6, IL-1β, NF-κB, and HIF-1α. In contrast, conditioned media from dysbiotic cancer-microbiota interactions increased tumor cell viability to 120–140% of controls. These findings suggest an association between microbiota-derived metabolite landscapes, pathobiont fitness and epithelial responses under CRC-associated dysbiosis.

**Graphical abstract:**

**Figure:**
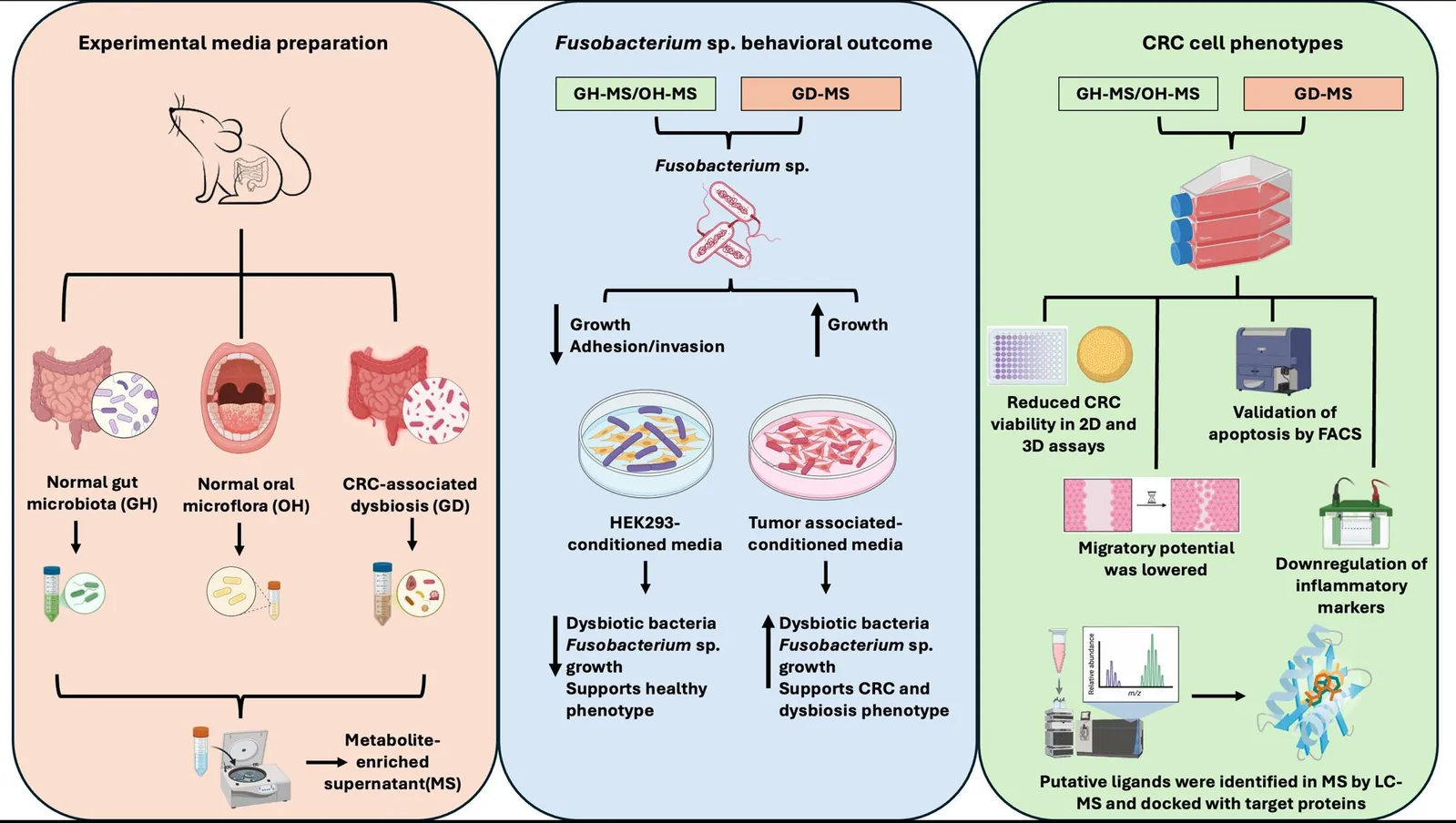

**Supplementary Information:**

The online version contains supplementary material available at https://doi.org/10.1186/s13099-026-00859-9.

## Introduction

The gut microbiota represents a highly complex ecological network whose metabolic activity plays a critical role in maintaining gut homeostasis [1, 2]. Rather than functioning solely as a collection of microbial taxa, these communities influence epithelial physiology, immune regulation, and cellular stress responses through the production of a wide array of metabolites that operate at the host-microbe interface [2]. As a result, disturbances within this ecosystem, commonly described as dysbiosis [3], are increasingly understood not simply as shifts in microbial composition, but as alterations in microbial functionality and host-microbial signaling dynamics [2, 4].

A common feature of dysbiosis across many gastrointestinal diseases is the reduction of microbial diversity accompanied by the expansion of opportunistic organisms known as pathobionts [5]. These resident microbes normally exist as commensals but can acquire pathogenic properties under certain conditions [5]. Among them, bacteria belonging to the genus *Fusobacterium* have repeatedly been reported to increase in abundance in both inflammatory and cancer-associated intestinal environments [6]. *Fusobacterium nucleatum* exhibits key virulence traits including biofilm formation, adhesion to epithelial cells, and invasion of host tissues, which facilitate its persistence within mucosal environments. These processes are mediated by adhesins such as FadA and Fap2 that promote binding to E-cadherin and enable bacterial internalization, thereby activating oncogenic and inflammatory signaling pathways in colorectal epithelial cells [7, 8]. Such adhesion and invasion mechanisms are directly relevant to its role in CRC progression and underpin the functional assays investigated in the present study. Although sequencing-based studies have consistently demonstrated correlations between *Fusobacterium* enrichment and disease states, such observations provide limited insight into the ecological mechanisms that enable its expansion, persistence and interaction with host tissues [9]. In particular, it remains uncertain whether the increased presence of *Fusobacterium* arises from inherent microbial characteristics or from environmental conditions created by altered microbial metabolism and host-derived factors.

Recent advances in microbiome research highlight the importance of microbiota-derived metabolites as central mediators of both microbial ecology and host-microbe communication [10, 11]. These small molecules can influence bacterial growth, competition between microbial species, and the expression of virulence-associated traits [12]. At the same time, they affect host epithelial signaling pathways involved in processes such as inflammation, hypoxia, barrier integrity, apoptosis, and cellular plasticity [11]. Importantly, the biological impact of microbial metabolites is highly context dependent and varies according to microbial community structure and the surrounding host environment [13]. Despite growing recognition of this metabolic dimension of microbiome function, the mechanistic link connecting dysbiosis-associated metabolite profiles, pathobiont behavior, and host epithelial responses remain incompletely understood.

Host-derived signals also contribute significantly to shaping microbial dynamics during dysbiosis [14]. Under conditions of epithelial stress or inflammation, host tissues release cytokines, redox mediators and metabolic by-products that may selectively promote the growth of certain microbial populations, thereby reinforcing dysbiotic conditions [15]. In contrast, interactions between healthy epithelial cells and commensal microbiota can produce soluble factors that restrict microbial overgrowth and help maintain intestinal balance [16, 17]. Clarifying how microbial metabolites interact with host-derived signals to influence bacterial fitness and epithelial function is therefore essential for understanding the functional consequences of dysbiosis beyond simple taxonomic changes [14].

In addition to gut-resident microbiota, microbial communities from other mucosal sites can influence intestinal microbial ecology and host physiology through shared metabolite pools and microbial translocation along the GI tract [18]. The oral cavity represents a major microbial reservoir whose taxa and metabolites can enter the GI system through continuous swallowing and dietary interactions [19]. Emerging evidence suggests that oral-gut microbial transmission contributes to intestinal dysbiosis and has been implicated in GI disorders, including cancer [20]. Notably, *Fusobacterium* species are common constituents of the oral microbiota and are capable of translocating to the gastrointestinal tract, where they have been implicated in colorectal cancer-associated dysbiosis [8]. This provides a functional rationale for evaluating oral microbiota-derived metabolite environments in the present study. Therefore, in addition to gut-derived microbiota metabolites, we included oral microbiota-derived metabolite-enriched supernatant as a comparative microbial metabolite environment to examine whether metabolites originating from distinct microbial niches exert differential effects on CRC-associated bacterial fitness and epithelial responses.

Colorectal cancer provides a tractable model system to investigate these interactions, as it is consistently associated with altered gut microbial composition, enrichment of *Fusobacterium*, and profound changes in the mucosal inflammatory and metabolic landscape [21]. However, antibiotic-induced microbial perturbation alone does not fully recapitulate disease-associated microbial signatures, suggesting that disease-specific ecological pressures contribute to shaping microbial function [22, 23]. Distinguishing nonspecific microbial disruption from functionally distinct dysbiotic states remains a key challenge in the field.

Most existing studies have focused either on cataloguing microbial taxa or on examining isolated host responses, often treating microbes and host tissues as independent variables [24]. Far fewer studies have directly examined how microbiota-derived metabolites simultaneously are associated with bacterial fitness and host epithelial behavior within the same experimental framework. Moreover, while *Fusobacterium* has been implicated in epithelial inflammation, immune modulation, and tissue remodeling, the extent to which its growth and host interactions are actively governed by community-level metabolite landscapes remains largely unexplored.

In this study, we address these gaps by integrating microbial community profiling with functional assays to define how dysbiosis-associated microbial states and their metabolite repertoires modulate *Fusobacterium* fitness and host epithelial responses. Using an orthotopic colorectal disease model as an ecological perturbation rather than a primary endpoint, we directly compare antibiotic-induced dysbiosis with disease-associated dysbiosis to disentangle generic microbial disruption from functionally distinct microbial states. By combining bacterial growth, adhesion, and invasion assays with 2D and 3D epithelial models, inflammatory and stress signaling analyses, and untargeted metabolomics, we move beyond descriptive microbiome profiling to functionally examine how microbial metabolite environments are associated with bacterial ecology and host-microbe signaling. Collectively, this work supports the role of microbiota-derived metabolites as potential higher-order modulators of dysbiosis-driven interactions, with implications extending beyond colorectal disease to a broad spectrum of intestinal disorders characterized by selective microbial expansion and altered host responses.

## Materials and methods

### Materials and reagents

All chemicals and reagents were of analytical or cell culture grade and used according to the manufacturer’s instructions. Key reagents included Dulbecco’s Modified Eagle Medium (DMEM), fetal bovine serum (FBS), Matrigel (Corning, USA), metronidazole, vancomycin, MTT reagent, and reagents required for LC-MS and molecular docking analyses. Detailed reagent information is provided in Supplementary methods.

### Orthotopic colorectal cancer model and microbiota sampling

Female BALB/c mice (6–8 weeks old, 18–22 g) were housed under specific pathogen-free conditions with ad libitum access to chow and water. Mice from different experimental groups were housed separately to prevent cross-transfer of microbiota. They were used to generate two related models of gut dysbiosis: (i) antibiotic-induced dysbiosis, and (ii) CRC-associated dysbiosis superimposed on antibiotic treatment. To induce baseline gut dysbiosis, mice were administered a combination of ciprofloxacin (500 mg/L) and metronidazole (1000 mg/L) in RO water for 14 consecutive days, which was refreshed every 2 days. This antibiotic regimen was selected to broadly disrupt the gut microbial community and reduce microbial diversity without inducing overt host pathology [25–27]. Following antibiotic treatment, a subset of mice was subjected to orthotopic colorectal cancer induction. CT26 murine colorectal carcinoma cells were cultured in DMEM (10% FBS, 1% penicillin-streptomycin), harvested at 70–80% confluence, resuspended in PBS at 6 $\:\times\:$ 10^7^ cells/mL, and mixed with 1:1 Matrigel. Mice were anesthetized (isoflurane: 5% induction, 2–3% maintenance), and cells were orthotopically injected into the distal colon wall. Mice received postoperative analgesia and were monitored daily for weight, activity, stool and signs of distress [28, 29]. Fresh fecal pellets were collected directly into sterile tubes and immediately processed within < 15 min. Samples were stored at −80 °C until further use. Fecal pellets were collected weekly to monitor microbial changes. Tumor development was assessed by abdominal palpation and general condition from day 7 post-implantation. At day 21, fecal pellets were collected (6 mice per group) for bacterial isolation and downstream analyses.

### 16 S rRNA gene sequencing

Fecal pellets collected from six mice per group were pooled prior to DNA extraction and sequencing in order to obtain group-level microbial community profiles. Genomic DNA was extracted from the pooled fecal samples using a commercial stool DNA extraction kit (Xploregen, India) according to the manufacturer’s protocol. DNA quality was assessed by NanoDrop spectrophotometry (260/280 ratio) and agarose gel electrophoresis prior to amplification. The V3-V4 region of the 16 S rRNA gene was amplified using universal primers (341 F/806R), and libraries were prepared using Illumina-compatible barcoded adapters. Paired-end sequencing (2 $\:\times\:$ 300 bp) was performed on an Illumina MiSeq platform [30]. Raw reads were quality-checked, filtered, merged and clustered into operational taxonomic units (OTUs). Taxonomic assignment was performed using a NCBI 16 S rRNA database. Alpha- and beta-diversity analyses (Chao1, Shannon, Simpson and Bray-Curtis PCoA) and taxonomic comparison were conducted using standard microbiome analysis pipelines [31, 32]. Detailed methodology has been provided in supplementary methods.

### Generation of microbiota-derived metabolite-enriched supernatants

Fresh fecal pellets from healthy (GH) and tumor-bearing (GD) mice were aseptically inoculated into thioglycolate broth and incubated anaerobically at 37 °C for 48 h. Cultures were grown to OD_600_ 0.7–1.0, centrifuged at 5,000 rpm for 15 min at 4 °C, and supernatants were passed through 0.22 μm filters to obtain cell-free “metabolite-enriched supernatants” (MS) (GH-MS and GD-MS) [33]. The term ‘metabolite-enriched supernatants’ refers to complex cell-free microbial secretomes and does not exclusively represent purified metabolites. Oral microbiota-derived metabolites (OH-MS) were prepared similarly using buccal swabs from healthy mice [34, 35]. Probiotic-derived MS were generated by inoculating 100 mg of lyophilised probiotic cultures (*Lactobacillus casei* strain DN-114 001, *Lactobacillus rhamnosus* strain LR32, *Lactobacillus plantarum* strain LP-115 and *Bifidobacterium lactis* strain BB-12) into 5 ml of MRS broth and incubated anaerobically at 37°C. Cultures were scaled up, centrifuged and filtered to obtain probiotic-derived cell-free, filter-sterilized metabolite-enriched supernatants. Sterile broth controls were processed identically to metabolite samples, including filtration and storage at −20°C. All supernatants were stored at −20°C and used within four weeks.

### Isolation and culture of CRC-associated bacteria

Bacterial experiments were conducted using a gut-derived bacterial isolate (entire mixed culture of fecal sample obtained from mice harboring orthotopic tumor) and a putative *Fusobacterium* sp., (enriched in CRC-associated dysbiosis, isolated from the mixed culture). Fresh fecal pellets were processed under anaerobic conditions. Samples were immediately transferred into thioglycollate medium to maintain an oxygen-limited environment and homogenized by gentle vortexing. The resulting suspension was incubated anaerobically at 37 °C to enrich anaerobic bacterial populations prior to plating on selective media for isolation of CRC-associated *Fusobacterium* sp. Selective isolation of *Fusobacterium* sp. was performed on an anaerobic blood agar (defibrinated 5% mouse blood) base supplemented with vancomycin (5 µg/mL) and neomycin (50 µg/mL), following historical selective media formulations. It was also supplemented with hemin and vitamin K, and incubated anaerobically at 37 °C for 48–72 h [36]. Isolates were further validated based on antibiotic selection, hemolytic activity on blood agar [37], and basic biochemical profiling. Representative bacterial colonies obtained under selective anaerobic conditions were further characterized by Gram staining and field emission scanning electron microscopy (FE-SEM). For Gram staining, bacterial smears were heat-fixed on glass slides and stained using standard crystal violet-iodine staining followed by alcohol decolorization and safranin counterstaining. Stained preparations were visualized under an oil immersion objective microscope.

For FE-SEM analysis, bacterial pellets were fixed in 2.5% glutaraldehyde at 4 °C overnight, washed with 1X PBS, and dehydrated through a graded ethanol series. Samples were dried, sputter-coated with gold, and imaged using a field emission scanning electron microscope under high vacuum conditions. Morphological observations from Gram staining, FE-SEM, selective anaerobic culturing conditions, and biochemical profiling collectively supported phenotypic characteristics consistent with *Fusobacterium* spp.

Based on selective culturing conditions, anaerobic growth, antibiotic selection, biochemical profiling, and morphological validation, the isolate was classified as a putative *Fusobacterium* sp. Community-level 16 S rRNA sequencing further supported the presence of *Fusobacterium* (notably *F. varium*) in CRC-associated dysbiosis samples. All bacterial work was conducted under BSL-2 conditions following institutional biosafety guidelines.

### Bacterial growth inhibition and regrowth assays

Effect of MS on bacterial growth was assessed using a broth-based growth kinetics assay. Cultures of the GD bacterial isolate and *Fusobacterium* sp. were grown to mid-log phase and adjusted to an optical density (OD₆₀₀) of 0.08–0.1. Standardized bacterial suspensions were transferred into sterile, capped culture tubes containing thioglycolate media supplemented with GH- or OH-MS metabolite preparations at final concentrations of 5% and 10% (v/v). Untreated bacterial cultures grown in broth without metabolites served as controls. All cultures were incubated under anaerobic conditions at 37 °C. Bacterial growth was monitored by measuring OD₆₀₀ at designated time intervals to generate growth curves. Growth inhibition was determined by comparing OD₆₀₀ values of treated cultures to untreated controls over time. To distinguish bacteriostatic and bactericidal effects, treated cultures were washed and re-inoculated into fresh medium and regrowth was monitored for 48 h [38, 39]. Experiments were conducted in triplicate and repeated independently to ensure reproducibility.

### Bacterial adhesion and invasion assays

HCT116 cells were seeded in 24-well plates at the density of 10,000 per well and grown to confluence in antibiotic-free DMEM [7, 40]. *Fusobacterium* sp. used for infection experiments were grown to mid-log phase and used as planktonic cultures. For adhesion and invasion assays, bacteria were incubated with epithelial cells for 4 h prior to antibiotic treatment to allow host-bacteria interaction and surface attachment. The *Fusobacterium* sp. was harvested from mid-log cultures, washed, and resuspended in DMEM to achieve a multiplicity of infection (MOI) of 100 bacteria per cell [41]. For host pre-treatment, HCT116 monolayers were incubated with 5% OH- and GH-MS for 4 h before infection; for bacteria pre-treatment, bacteria were incubated with 5% OH- and GH-MS for 60 min before infection; combined conditions included both. Cells were infected for 4 h at 37 °C. For adhesion assays, cells were washed with PBS to remove non-adherent bacteria. Cells were collected by mechanical scraping and adhered bacteria were quantified by plating it on selective blood agar under anaerobic conditions for CFU enumeration. For invasion assays, extracellular bacteria were eliminated using gentamicin (300 g/mL) and metronidazole (200 $\:\mu\:$g/mL) for 1 h prior to cell lysis, ensuring that only intracellular (invaded) bacteria were quantified by CFU enumeration. To validate the efficacy of antibiotic treatment against surface-associated *Fusobacterium* sp., bacteria were incubated in tissue culture-treated plates for 4 h under anaerobic conditions to allow surface attachment. Wells were gently washed to remove non-adherent bacteria and treated with gentamicin (300 µg/mL) and metronidazole (200 µg/mL) for 1 h. Following treatment, bacteria were collected by scraping and plated on selective blood agar for CFU enumeration. Statistical comparisons between control and antibiotic-treated groups were performed separately for planktonic and surface-associated conditions using unpaired two-tailed Student’s t-test with Welch’s correction. CFU values were normalized to cell number and expressed as CFU per 10⁶ cells. For comparative analysis, adhesion and invasion were additionally expressed as percentage of infected control (set to 100%) [42].

### Mammalian cell culture models

CT26, HCT116, and HEK293 cells were cultured in DMEM supplemented with 10% FBS and 1% penicillin-streptomycin at 37 °C in 5% CO₂. Cells were passaged at 70–80% confluence and used between passages 15–20.

### Host-microbiota interaction-derived conditioned media

To evaluate the effects of soluble factors generated during host-microbiota interactions, host-microbiota conditioned media (HM-CM) were prepared from two distinct co-culture settings. Mammalian cells were cultured in DMEM supplemented with 10% FBS and seeded at a density of 2 $\:\times\:$ 10⁵ cells per well in 6-well plates and incubated overnight. Prior to co-culture, mammalian cells were maintained in antibiotic-free DMEM supplemented with 1% FBS to support cell viability while minimizing potential complement-mediated effects on bacterial cells during the co-culture period. Bacterial cultures were grown separately in their respective broth media to mid-log phase, adjusted to an optical density (OD₆₀₀) of 0.1 and diluted in sterile PBS. For HEK293-probiotic HM-CM, HEK293 cells were co-cultured with probiotic bacteria at a multiplicity of infection (MOI) of 50:1 for 12 h in antibiotic-free medium [43, 44]. For CT26- and HCT116-derived HM-CM, CT26 or HCT116 cells were co-cultured with GD bacteria at an MOI of 50:1 for 4 h under identical culture conditions. At the end of incubation, supernatants were collected, centrifuged at 5000 rpm for 15 min at 4 °C and passed through a 0.22 μm membrane filter to obtain sterile, cell-free host-microbiota conditioned media (HM-CM). Control media were processed in parallel from uninfected cell cultures and bacteria-only cultures and used as negative and bacteria-only controls, respectively.

### Microbiota- and host-conditioned media effects on cancer cell behaviour

Cell viability was assessed using MTT assays in 2D and 3D spheroid models. To evaluate whether the effects of MS are specific to cancer cells, HEK293 cells were treated with GH-, OH-, and GD-MS under conditions identical to CRC cell experiments, and viability was assessed using the MTT assay. Migration was evaluated by scratch assays. Apoptosis was quantified using Annexin V/PI flow cytometry. Western blotting was performed using antibodies against EMT, apoptotic, inflammatory, and hypoxia markers, with β-actin as a loading control. Detailed methodology has been given in supplementary methods.

### Biochemical analysis of MS

To investigate the biochemical nature of the active compounds present in MS, GH- and OH-MS were subjected to heat treatment, proteinase K digestion and pH normalization prior to cell treatment. For heat-treatment, MS were incubated at 80 °C for 30 min, cooled to room temperature and centrifuged to remove precipitated material. For proteinase-K digestion, MS were incubated with proteinase-K (100$\:\mu\:$g/mL) at 37 °C for 2 h followed by enzyme inactivation at 95 °C for 10 min. For pH normalization, MS were adjusted to physiological pH 7.2–7.4 using sterile NaOH. Treated supernatants were subsequently used for MTT-based viability assays in CT26 and HCT116 cells under conditions identical to untreated MS experiments. To functionally validate representative metabolites identified by LC-MS profiling, sodium butyrate and phenylacetic acid were individually dissolved in sterile culture medium and applied to CRC cells at indicated concentrations followed by MTT analysis.

### In silico analysis

Untargeted LC-QTOF-MS analysis was performed on pooled microbiota-derived MS to profile differentially abundant metabolites across experimental groups. LC-MS chromatograms are given as separate supplementary pdf files. Metabolite annotation was based on accurate mass matching and was considered putative. Metabolite peak intensities were visually assessed and categorized as low, moderate, or high based on relative signal strength. (Supplementary Table S3). No targeted metabolite validation or absolute quantification was performed. Relative metabolite abundance analysis was done by Q-TOF. Selected metabolites were used for hypothesis-generating molecular docking analyses against host proteins involved in epithelial integrity, inflammation, and apoptosis. Detailed methodology has been given in supplementary methods.

### Statistical analysis

All experiments were performed with a minimum of three independent biological replicates (*n* = 3), and data are presented as mean ± SEM. Statistical analyses were conducted using GraphPad Prism version 10.0. Differences between two groups were evaluated using an unpaired two-tailed Student’s *t*-test, while comparisons among multiple groups were performed using one-way or two-way ANOVA followed by Dunnett’s post hoc test. A *p* value < 0.05 was considered statistically significant.

## Results

### CRC-associated dysbiosis is characterized by a pronounced loss of gut microbial diversity

**Fig. 1:**
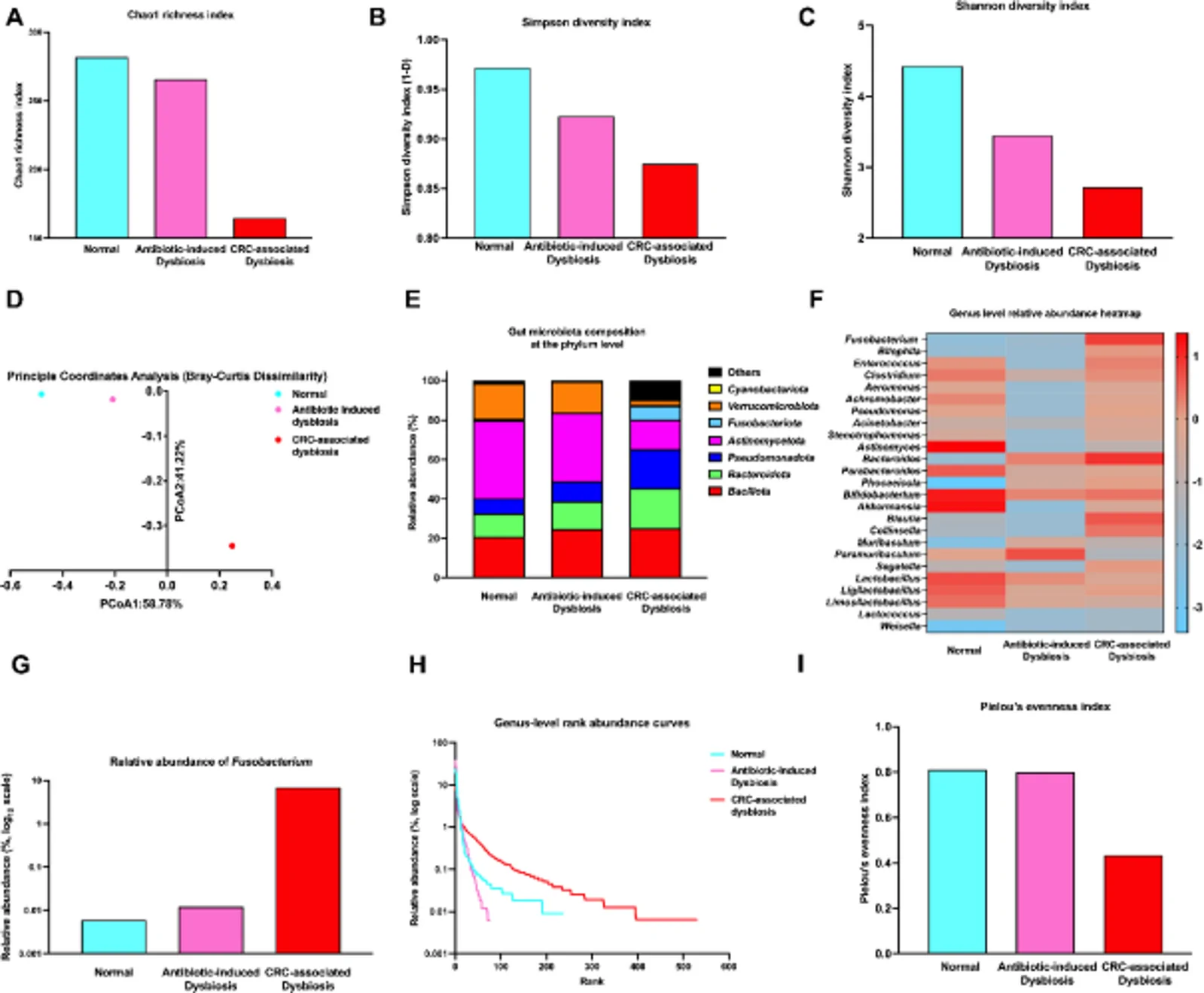
Gut microbiota diversity and compositional alterations in normal, antibiotic-induced dysbiosis, and CRC-associated dysbiosis. Fecal samples from six mice per group were pooled prior to DNA extraction and 16S rRNA sequencing to generate group-level microbiome profiles. Alpha diversity analyses revealed a reduction in microbial richness and diversity in dysbiotic mice compared with normal controls, as assessed by the Chao1 richness index (**A**) Simpson diversity index (**B**) and Shannon diversity index (**C**); (**D**) Principal coordinates analysis (PCoA) based on Bray-Curtis dissimilarity illustrates differences in community composition among normal, antibiotic-induced dysbiosis, and CRC-associated dysbiosis groups, indicating marked differences in overall community structure; (**E**) Phylum-level composition of the gut microbiota represented as stacked bar plots, illustrating global shifts in microbial community structure across experimental groups. Low-abundance phyla were grouped as “Others” for clarity; (**F**) Heatmap showing scaled (Z-score) log₁₀-transformed relative abundances of selected gut-associated bacterial genera across experimental groups. CRC-associated dysbiosis showed enrichment of CRC-associated and opportunistic taxa (e.g., *Fusobacterium*, *Bilophila*, *Bacteroides*) and depletion of beneficial commensals such as *Akkermansia*, *Bifidobacterium*, and *Lactobacillus;* (**G**) Relative abundance of *Fusobacterium* plotted on a log₁₀ scale, highlighting a marked enrichment in CRC-associated dysbiosis compared with normal and antibiotic-induced dysbiosis groups; (**H**) Rank abundance profile showing the distribution and relative abundance of microbial taxa across the three groups. The curve illustrates species richness and evenness within each microbial community; and **(I)** Pielou’s evenness index comparing microbial community evenness among the three groups

To evaluate the impact of antibiotic exposure and colorectal cancer on gut microbial diversity, 16 S rRNA gene sequencing was performed on fecal samples collected from normal mice, antibiotic-induced dysbiosis mice, and CRC-associated dysbiosis mice. Because fecal samples were pooled prior to sequencing, diversity metrics and taxonomic profiles reflect group-level microbiome composition rather than inter-individual variation. Accordingly, these analyses are presented as ecological context to guide functional experiments rather than as statistically powered comparisons of microbial diversity. The samples had uniform library size, comparable GC content and sequencing depth across groups, ensuring robustness of downstream microbiome analyses (Supplementary Fig. S1 and S2; Table S1). Alpha diversity analyses suggested a progressive reduction in microbial richness and diversity across the three groups (Fig. 1A-C). Collectively, these descriptive findings are consistent with CRC-associated dysbiosis exhibiting a more pronounced reduction in richness and diversity than antibiotic-induced dysbiosis alone, recognizing that pooled samples preclude statistical comparison. To visualise overall differences in microbial community structure, beta diversity was examined using principal coordinate analysis (PCoA) based on Bray-Curtis dissimilarity. The pooled microbiome profiles from normal, antibiotic-induced dysbiosis and CRC-associated dysbiosis groups occupied distinct positions in ordination space (Fig. 1D), indicating differences in overall community composition across the three ecological states. Although these groups are presented for comparative analysis, antibiotic-induced dysbiosis and CRC-associated dysbiosis represent distinct biological states rather than a linear progression. To further contextualize taxonomic changes, phylum-level microbial composition was analysed. Stacked bar plots revealed global alterations in community structure across experimental groups (Fig. 1E). CRC-associated dysbiosis microbiome profile showed an increased representation of Fusobacteriota and Pseudomonadota, phyla commonly associated with inflammation and dysbiosis, alongside reductions in health-associated phyla such as Verrucomicrobiota and Actinomycetota. To identify taxonomic features underlying the observed community shifts, genus-level relative abundances were examined. Core microbiome and heatmap analysis revealed distinct patterns of microbial enrichment and depletion across experimental groups (Fig. 1F; Supplementary Fig. S3-S4). CRC-associated dysbiosis was characterized by genera previously reported to be associated with CRC or inflammatory conditions, including *Fusobacterium* and *Bacteroides*. In contrast, beneficial commensal taxa such as *Akkermansia*, *Bifidobacterium*, *Lactobacillus*, and *Muribaculum* were depleted in dysbiotic conditions, with the most pronounced reductions observed in CRC-associated dysbiosis. Given the established association of *Fusobacterium* with CRC, its relative abundance was examined in detail. *Fusobacterium* abundance was low in normal mice and modestly increased following antibiotic treatment; however, a marked enrichment was observed in CRC-associated dysbiosis (Fig. 1G). These observations suggest that *Fusobacterium* enrichment is more pronounced in CRC-associated dysbiosis than in antibiotic-induced dysbiosis alone. Rank abundance analysis indicated differences in microbial distribution patterns among the groups, with CRC-associated dysbiosis displayed an extended rank distribution with altered abundance patterns compared with normal microbiota (Fig. 1H). Consistently, Pielou’s evenness index was lower in the CRC-associated dysbiosis group compared with normal and antibiotic-induced dysbiosis groups (Fig. 1I). Together, these results suggest that CRC-associated dysbiosis represents a distinct microbial state characterized by both loss of beneficial taxa and expansion of disease-associated lineages.

### GH-MS and OH-MS constrain CRC-associated bacterial growth

**Fig. 2:**
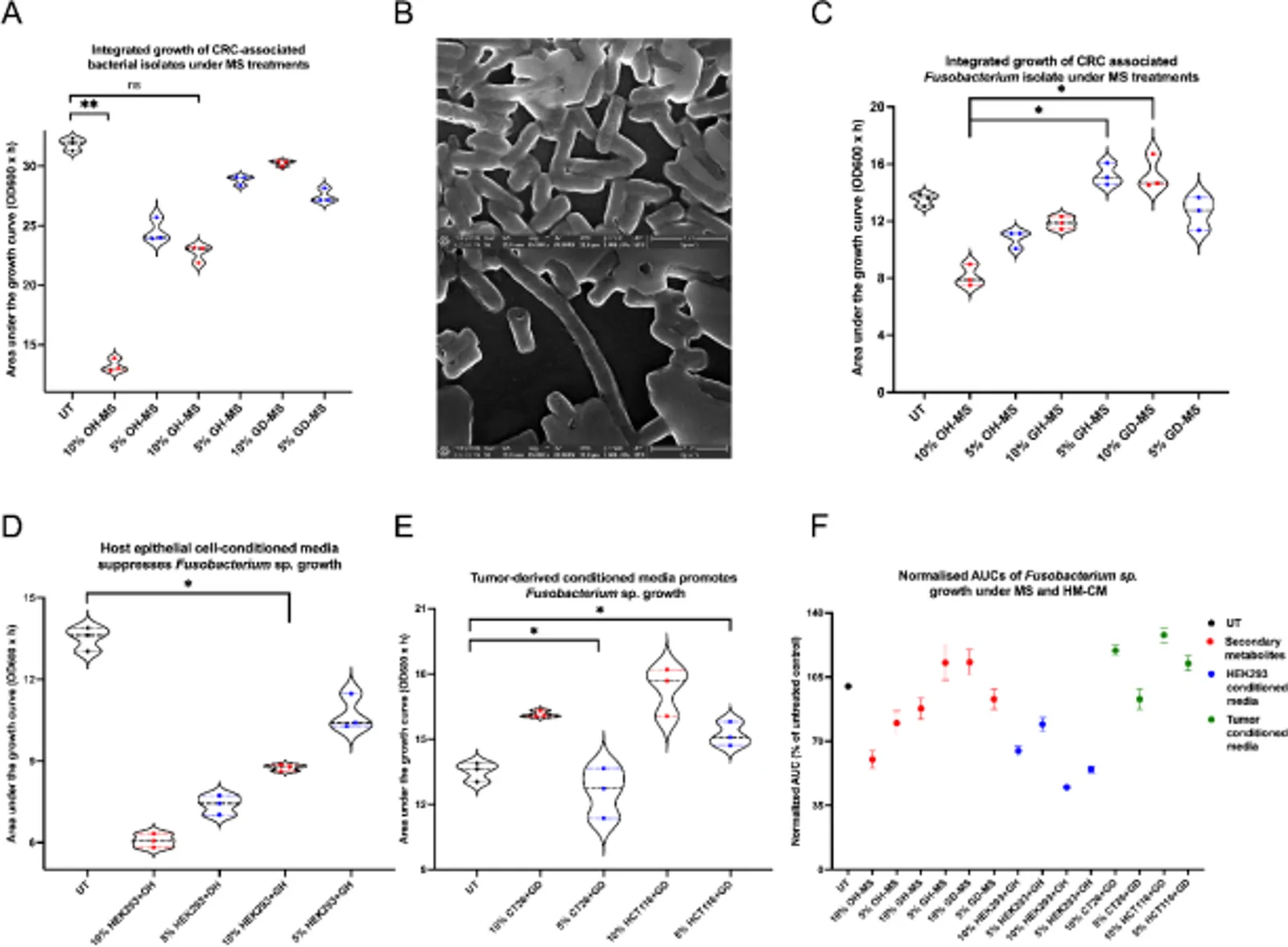
MS-, host-, and tumor-derived cues differentially influence the growth of CRC-associated bacteria and *Fusobacterium* sp. (**A**) Integrated growth of CRC-associated bacterial isolates under MS treatments. Bacterial growth was monitored over time by OD₆₀₀, and integrated growth was quantified as area under the curve (AUC). Metabolite-enriched supernatants derived from different conditions (OH-MS, GH-MS, and GD-MS at 5% and 10%) elicited distinct effects on CRC-associated bacterial growth compared with untreated (UT) controls; (**B**) FE-SEM image of isolated Fusobacterium sp. (Scale bar = 3$\:\mu\:$m); (**C**) Integrated growth of isolated *Fusobacterium* sp. under MS treatments, demonstrating differential sensitivity of this CRC-associated pathobiont to metabolite-derived cues; (**D**) Effect of host-derived conditioned media (HM-CM) from HEK293 cells on *Fusobacterium* sp. growth, showing suppression of bacterial growth relative to untreated controls; (**E**) Effect of tumor-derived conditioned media from CT26 and HCT116 colorectal cancer cells on *Fusobacterium* sp. growth, indicating growth-promoting effects in a tumor-associated microenvironment; (**F**) Summary of normalized AUC values for *Fusobacterium* sp. growth under MS, host-derived conditioned media (HEK293-CM), and tumor-derived conditioned media (CT26/HCT116-CM). AUC values were normalized to untreated controls (UT = 100%) to allow direct comparison across conditions. Data are shown as mean ± SEM from three independent experiments. Statistical significance was assessed using the Kruskal-Wallis test with Dunn’s post hoc comparisons. *p* < 0.05 was considered statistically significant

GH-, OH- and GD-MS were evaluated for their effects on bacterial growth. To screen how distinct microenvironmental cues shape the growth behavior of CRC-associated gut microbes, we assessed the effects of MS, host-derived conditioned media (HM-CM), and tumor-derived conditioned media on bacterial growth dynamics. At the community level, MS significantly altered the integrated growth of CRC-associated bacteria (Fig. 2A). While OH-MS exerted a pronounced inhibitory effect on bacterial growth, GH-MS partially reduced growth, and GD-MS did not have any effect, suggesting that microenvironment-associated metabolic cues may differentially influence CRC-associated microbial growth under in-vitro conditions. Given the established association of *Fusobacterium* with CRC, we next focused on this pathobiont as a representative CRC-associated bacterium. Microbiome sequencing revealed the presence of *Fusobacterium varium* in CRC-associated dysbiosis samples, whereas *Fusobacterium mortiferum* was detected in antibiotic-induced dysbiosis communities. To investigate how *Fusobacterium* responds to microbial metabolites and host-derived factors, a putative *Fusobacterium* isolate (hereafter referred to as *Fusobacterium* sp.) was cultured from fecal samples obtained from CRC-associated dysbiosis mice and used for subsequent growth assays (Supplementary Fig. S5). Basic biochemical profiling revealed phenotypic characteristics consistent with *Fusobacterium* species (Supplementary Table S2). Representative gram stain images and FE-SEM images also support the presence of *Fusobacterium sp.* (Fig. 2B and Supplementary Fig S6). The isolate is referred to as *Fusobacterium* sp. based on phenotypic and selective characteristics; however, definitive taxonomic identification was not performed. Consistent with community-level observations, *Fusobacterium* sp. displayed differential growth responses to MS (Fig. 2C). OH-MS markedly suppressed bacterial growth, whereas GD-MS supported increased growth, indicating that microbiota-derived metabolic cues are associated with changes in *Fusobacterium* sp. growth dynamics. We next examined the influence of host-derived cues on *Fusobacterium* sp. growth. Conditioned media from non-tumorigenic HEK293 cells significantly suppressed *Fusobacterium* sp. growth compared with untreated controls (Fig. 2D), suggesting that a non-malignant epithelial context may impose constraints on the expansion of this CRC-associated pathobiont. In contrast, conditioned media derived from colorectal cancer cell lines (CT26 and HCT116) promoted *Fusobacterium* sp. growth (Fig. 2E). This tumor-associated permissiveness highlights a potential mechanism by which the colorectal tumor microenvironment selectively supports the expansion of CRC-associated pathobionts. To integrate these findings across experimental conditions, we normalized growth AUC values to untreated controls and compared responses across stromal, host, and tumor-derived environments (Fig. 2F). This summary analysis revealed a clear gradient in *Fusobacterium* sp. growth responses, with host-derived cues suppressing growth, and tumor-derived cues strongly enhancing bacterial fitness. Detailed growth kinetics (OD₆₀₀ over time) for all conditions are provided in the Supplementary Fig. S7, supporting the integrated AUC-based analyses presented here.

### GH-MS and OH-MS reversibly suppress *Fusobacterium* sp. growth and impair host interaction

**Fig. 3:**
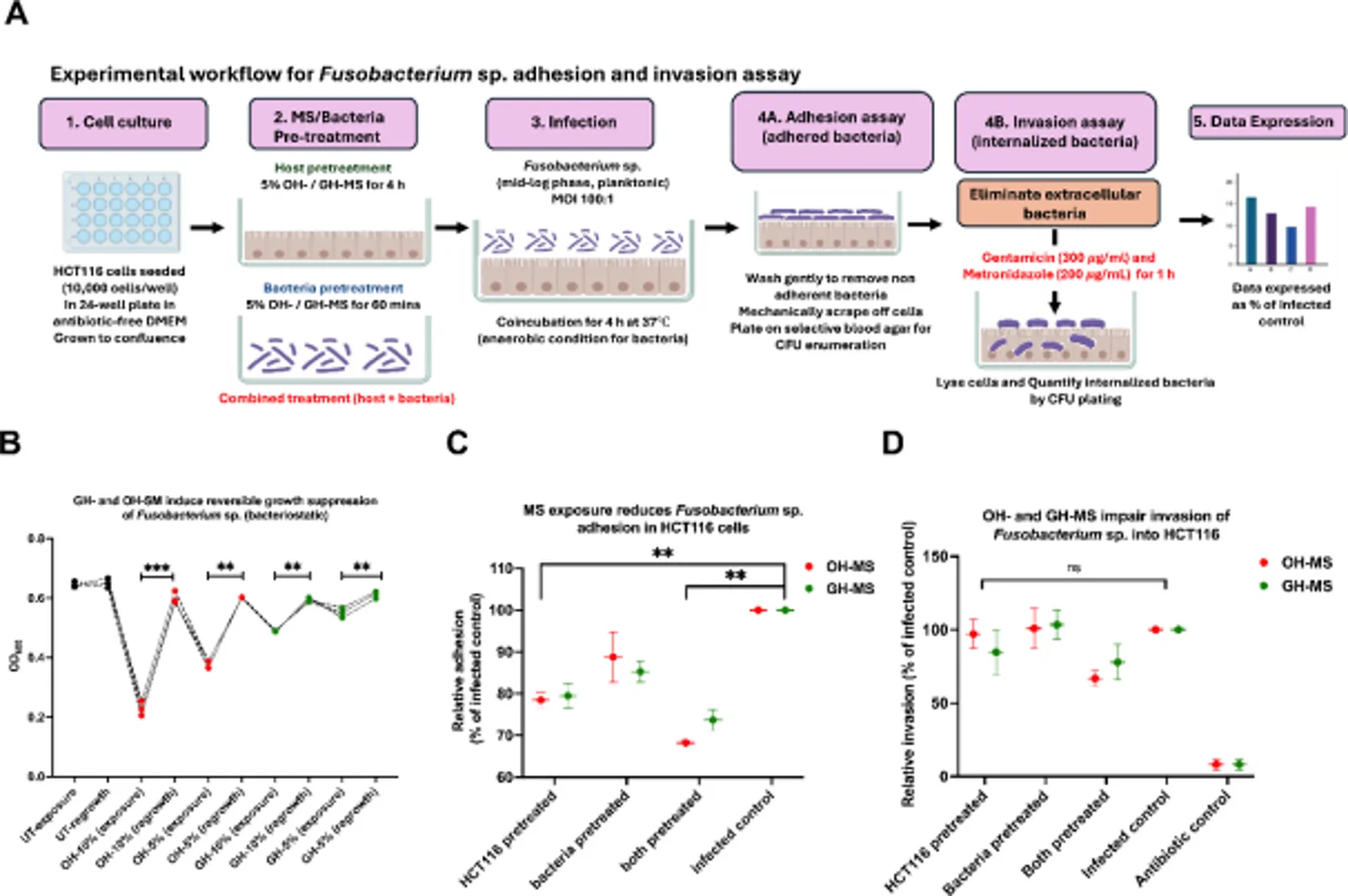
GH-MS and OH-MS induce reversible growth suppression and impair host interaction of *Fusobacterium* sp. (**A**) Schematic figure of adhesion and invasion assay of *Fusobacterium* sp. on HCT116 cells; (**B**) Paired growth analysis showing bacteriostatic suppression during MS exposure and recovery upon washout. Exposure vs. regrowth comparisons were analysed using paired t-test; (**C**) Adhesion of *Fusobacterium* sp. to HCT116 cells following pretreatment of host cells, bacteria, or both with OH-MS or GH-MS, quantified by CFU enumeration and expressed relative to infected control (set to 100%); (**D**) Invasion of *Fusobacterium* sp. into HCT116 cells under the same conditions. Antibiotic-treated samples served as negative controls. Data represent mean ± SEM from three independent experiments. Statistical significance was assessed using one-way ANOVA with Dunnett’s multiple comparisons test, where statistical comparisons were not significant in invasion assay

Exposure to GH- and OH-MS resulted in a reproducible suppression of *Fusobacterium* sp. growth under anaerobic conditions. Treatment with either GH- or OH-MS reduced bacterial growth by 55–65% during exposure, with growth fully recovering to near untreated levels following washout (*p* < 0.01 for exposure vs. regrowth across conditions), consistent with a bacteriostatic effect (Fig. 3B). We next evaluated the effect of MS exposure on *Fusobacterium* sp. adhesion and invasion in HCT116 epithelial cells (Schematic given in Fig. 3A). CFU-based quantification showed that pretreatment of either host cells or bacteria with GH- or OH-MS reduced bacterial adhesion by 15–25%, whereas combined pretreatment resulted in a greater reduction of 30–35% relative to infected control (*p* < 0.01, Fig. 3C). In absolute terms, adhesion decreased from 96 CFU/well in the control group to 65 CFU/well following combined pretreatment, corresponding to ~ 33% reduction. Consistent with these findings, MS exposure also impaired bacterial invasion. Pretreatment of host cells or bacteria alone reduced invasion by 10%, while combined pretreatment decreased invasion by 33%, (Fig. 3D). Absolute CFU-based quantification for both adhesion and invasion is provided in Supplementary Fig. S8.

To assess non-specific surface attachment, *Fusobacterium* sp. was incubated in tissue culture-treated wells in the absence of epithelial cells (Schematic given in Supplementary Fig. S9). Under these conditions, only ~ 1–2% of bacteria remained surface-associated after washing, indicating minimal adherence to plastic surfaces. In contrast, substantially higher adhesion was observed in the presence of HCT116 monolayers (Fig. 3C), consistent with host-dependent bacterial attachment. Antibiotic treatment significantly reduced bacterial survival under planktonic conditions (*p* < 0.05). A similar reduction was observed under surface-associated conditions, although this did not reach statistical significance (*p* ≈ 0.08), likely due to low baseline counts (Supplementary Fig. S10). These observations indicate that MS derived from healthy microbiota can transiently constrain the growth and host interaction capacity of CRC-associated bacteria. Detailed OD₆₀₀ measurements during MS exposure and after washout are shown in Supplementary Fig. S11.

### GH-MS and OH-MS suppress epithelial viability and spheroid growth in 2D and 3D models

**Fig. 4:**
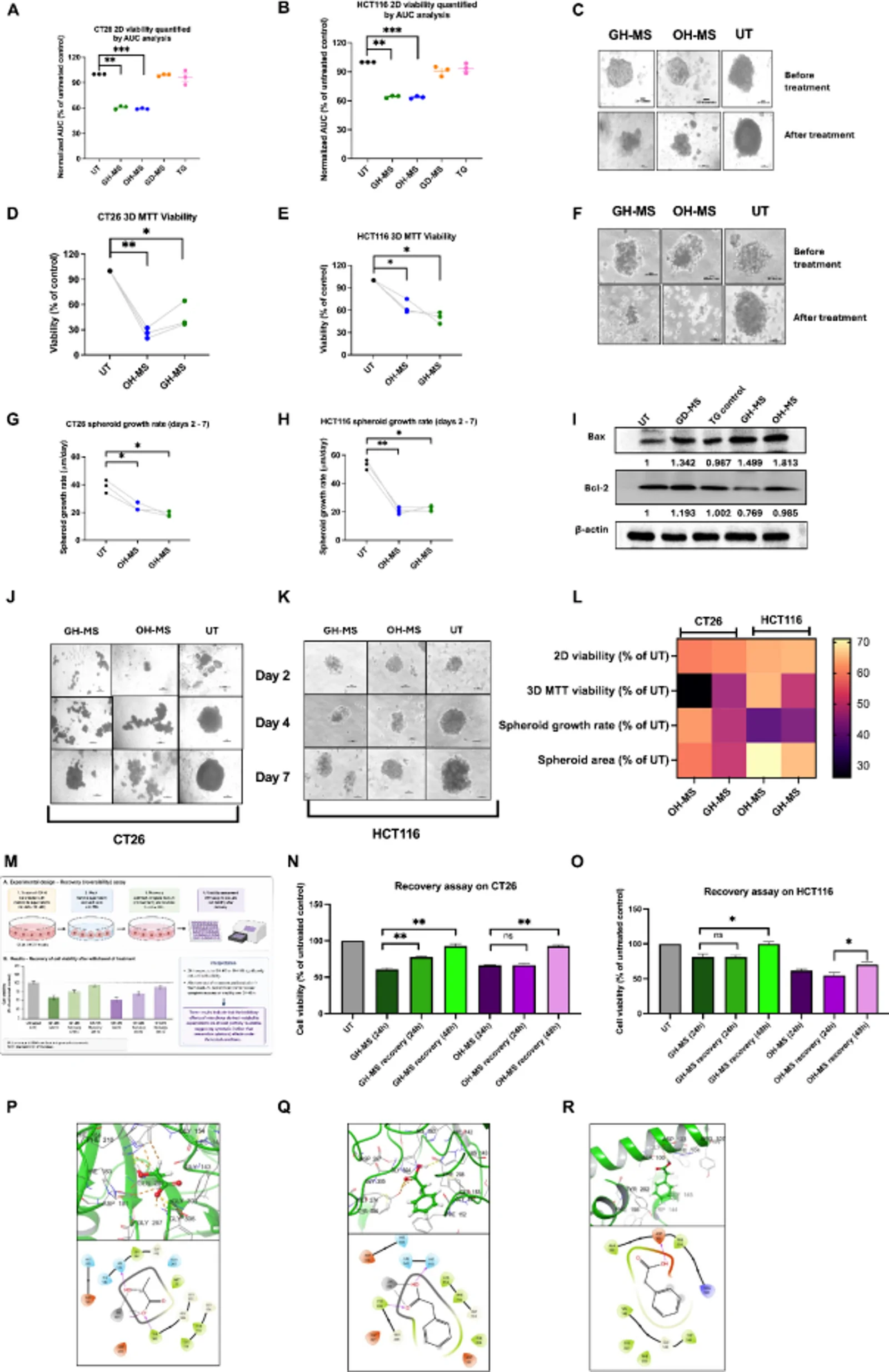
GH-MS and OH-MS suppress epithelial viability and spheroid growth in 2D and 3D models. (**A-B)** CT26 (**A**) and HCT116 (**B**) 2D epithelial viability quantified by AUC analysis following treatment with healthy gut-derived (GH-MS), oral-derived (OH-MS), CRC-associated gut (GD-MS), or thioglycolate media control (TG), expressed as percentage of untreated (UT) controls; (**D**,** E**) 3D spheroid metabolic viability of CT26 (D) and HCT116 (**E**) cells assessed by MTT assay following treatment with 5% (v/v) OH-MS or GH-MS. Individual data points represent matched independent experiments, with lines connecting paired measurements; (**C**,** F**) Representative brightfield images of pre-formed spheroids from CT26 (C) and HCT116 (**F**) 3D MTT experiments shown before and after treatment with OH-MS or GH-MS. Images are representative of three independent experiments and are shown for qualitative assessment of spheroid integrity. Scale bars = 100 μm; (**G-H**) Spheroid growth rates of CT26 (G) and HCT116 (H) cells calculated over the linear growth phase (days 2–7) following treatment with 2.5% (v/v) OH-MS or GH-MS. Growth rate is expressed as µm/day, with paired data shown for independent experiments; (**I**) Western blot analysis of apoptosis markers in CT26 cells following treatment (2.5%, v/v), showing Bax and Bcl-2 expression. β-actin was used as a loading control; (**J-K**) Representative day 2–7 spheroid morphology of CT26 and HCT116 cells from independent spheroid growth experiments treated with OH-MS or GH-MS, corresponding to growth-rate analyses. Scale bars = 100 μm; (**L**) Summary heatmap integrating normalized effects of OH-MS and GH-MS across 2D viability, 3D metabolic activity, spheroid growth rate, and spheroid area in CT26 and HCT116 models. Values represent mean responses expressed as percentage of untreated controls, with darker colors indicating stronger inhibition; (**M**) Schematic diagram of recovery assay; (**N**) and (**O**) Recovery assay in CT26 and HCT116 cells respectively following transient exposure to GH-MS and OH-MS; (**P-R**) Representative in silico docking poses illustrating predicted interactions between selected microbiota-derived metabolites and CRC-associated regulatory proteins (HDAC2, HDAC8, and BCL-2). Detailed interaction analyses are provided in the Supplementary Information. Data are presented as mean ± SEM from three independent experiments. Statistical significance was assessed using one-way ANOVA with Dunnett’s multiple comparisons test relative to untreated controls. Asterisks denote statistical significance (*p* < 0.05, *p < 0.01, **p < 0.001)

To assess the impact of MS on epithelial viability, CT26 and HCT116 cells were first examined in 2D monolayer cultures. Dose-response analyses revealed a concentration-dependent reduction in viability following exposure to both gut-derived (GH-MS) and oral-derived (OH-MS) supernatants. To summarize these effects across concentrations, area under the curve (AUC) analysis was performed to integrate dose-dependent effects across the tested concentration range rather than focusing on a single inhibitory concentration. In CT26 cells, normalized AUC values were reduced to approximately 59–61% of untreated controls following treatment with GH-MS and OH-MS, respectively, compared with 100% in untreated cells (Fig. 4A). Similarly, in HCT116 cells, GH-MS and OH-MS reduced normalized AUC values to 63–65% of control levels (Fig. 4B). In contrast, GD-MS and TG-MS exhibited minimal effects, with AUC values remaining close to untreated controls in both cell lines. Consistent reductions in 2D viability were also observed at 24 h and 72 h exposure time points (Supplementary Fig. S12), while 48 h MTT measurements are presented in the main figure as a representative intermediate time point used for subsequent analyses. Together, these results demonstrate that MS from both gut and oral microbiota impair epithelial viability in 2D culture across multiple exposure durations. To assess whether the observed effects were specific to cancer cells, we evaluated the impact of microbiota-derived MS on non-malignant epithelial cells (HEK293). HEK293 cells exhibited limited sensitivity to GH-MS and OH-MS, whereas GD-MS caused a greater reduction in viability at higher concentrations and longer exposure durations (Supplementary Fig. S13), indicating a differential sensitivity. However, lack of intestinal epithelial model limits tissue-specific interpretation. Along with it, a “protection assay” was performed on HEK293 cells where the pre-treatment with GH-/OH-MS prior to exposure with GD-MS partially restored cell-viability compared with GD-MS alone. This suggests a protective effect of healthy environment against dysbiotic environment (Supplementary Fig. S14). To determine whether these effects were preserved in more physiologically relevant systems, MS were next evaluated in 3D spheroid models. Both CT26 and HCT116 spheroids exhibited marked reductions in metabolic viability and growth following GH- and OH-MS treatment (Fig. 4D-E). Representative brightfield images acquired before and after treatment illustrate pronounced disruption of spheroid integrity following exposure to both GH-MS and OH-MS in CT26 and HCT116 models (Fig. 4C, F respectively). To test whether MS also impair spheroid formation and expansion, independent spheroid growth experiments were performed. In CT26 spheroids, untreated cells exhibited growth rates of 35–45 μm/day, whereas OH-MS and GH-MS treatment reduced growth rates to 20–25 μm/day and 15–20 μm/day, respectively (Fig. 4G). A similar pattern was observed in HCT116 spheroids, where growth rates decreased from 50 to 55 μm/day in untreated controls to 20–25 μm/day following treatment with either OH-MS or GH-MS (Fig. 4H). Representative spheroid images from these growth experiments further illustrate reduced spheroid size and altered morphology in MS-treated conditions (Fig. 4J, K**)**. To support the cytotoxic effects, expression levels of apoptotic markers Bax and Bcl2 was assessed by western blotting in CT26 cells where a marked increase in Bax was observed and Bcl2 reduction was evident (Fig. 4I, Supplementary Fig. S12G). Finally, an integrated summary heatmap was generated to visualize normalized effects of OH-MS and GH-MS across all phenotypic readouts, including 2D viability, 3D metabolic activity, spheroid growth rate, and spheroid area (Fig. 4L). When expressed as percentages relative to untreated controls, both GH- and OH-MS consistently reduced epithelial viability and spheroid growth across CT26 and HCT116 models, with stronger inhibitory effects observed in 3D assays than in 2D systems. To assess the reversibility of the observed effects, recovery assays were performed following treatment withdrawal. Cell viability progressively recovered following replacement with fresh medium, with greater recovery observed after 48 h than after 24 h, although the extent of recovery varied between the cell lines and treatment conditions (Fig. 4M-O). To explore potential molecular mechanisms underlying the effects of microbiota-derived metabolites on CRC cell viability and apoptosis, we performed in-silico interaction analyses using representative putative metabolites identified in GH-/OH-MS. Docking simulations suggested that small microbial metabolites such as lactate and phenylacetic acid can interact with host regulatory proteins associated with CRC survival pathways. Lactate was predicted to bind within the catalytic pocket of HDAC2 with a docking score of −7.88 (Fig. 4P), while phenylacetic acid displayed favorable interactions within the catalytic channel of HDAC8 with a docking score of −7.82 (Fig. 4Q). In addition, phenylacetic acid showed potential binding within the BH3 interaction groove of BCL-2, a key anti-apoptotic regulator (Fig. 4R). It gave a docking score of −4.85. These predicted interactions are consistent with the observed reduction in CRC cell viability and modulation of Bax/Bcl-2 following treatment with MS. Detailed binding information of HDAC2, HDAC8 and Bcl-2 with all identified metabolites is provided in Supplementary_figures Sect. 4 and Supplementary Fig. S16, S17, S18. To further investigate the biochemical nature of the active components present in microbiota-derived MS, heat-treated, proteinase-K digested, and pH-normalized MS treatment was given to CRC and HCT116 cells. Notably, treated supernatants retained substantial anti-proliferative activity comparable to untreated MS preparations (Supplementary Fig. S15A-B). These findings are consistent with the involvement of heat-stable small molecular components within the MS. To examine whether representative metabolites identified by LC-MS profiling contribute to the observed epithelial responses, sodium butyrate and phenylacetic acid were selected for individual functional validation. Treatment of CT26 and HCT116 cells with these metabolites resulted in a concentration-dependent reduction in CRC cell viability, with sodium butyrate producing comparatively stronger anti-proliferative effects (Supplementary Fig. S15C-F). Although these experiments do not establish direct causality within the complex metabolite mixtures, they demonstrate that individual microbiota-derived metabolites can partially recapitulate the anti-proliferative phenotype observed with healthy microbiota-derived MS. Collectively, these data demonstrate that MS derived from healthy gut and oral microbiota exert robust and reproducible inhibitory effects on epithelial viability and 3D spheroid growth, highlighting the importance of three-dimensional model systems for capturing microbiota-derived functional impacts on epithelial biology.

### MS suppress colorectal cancer cell migration and modulate EMT-associated markers

**Fig. 5:**
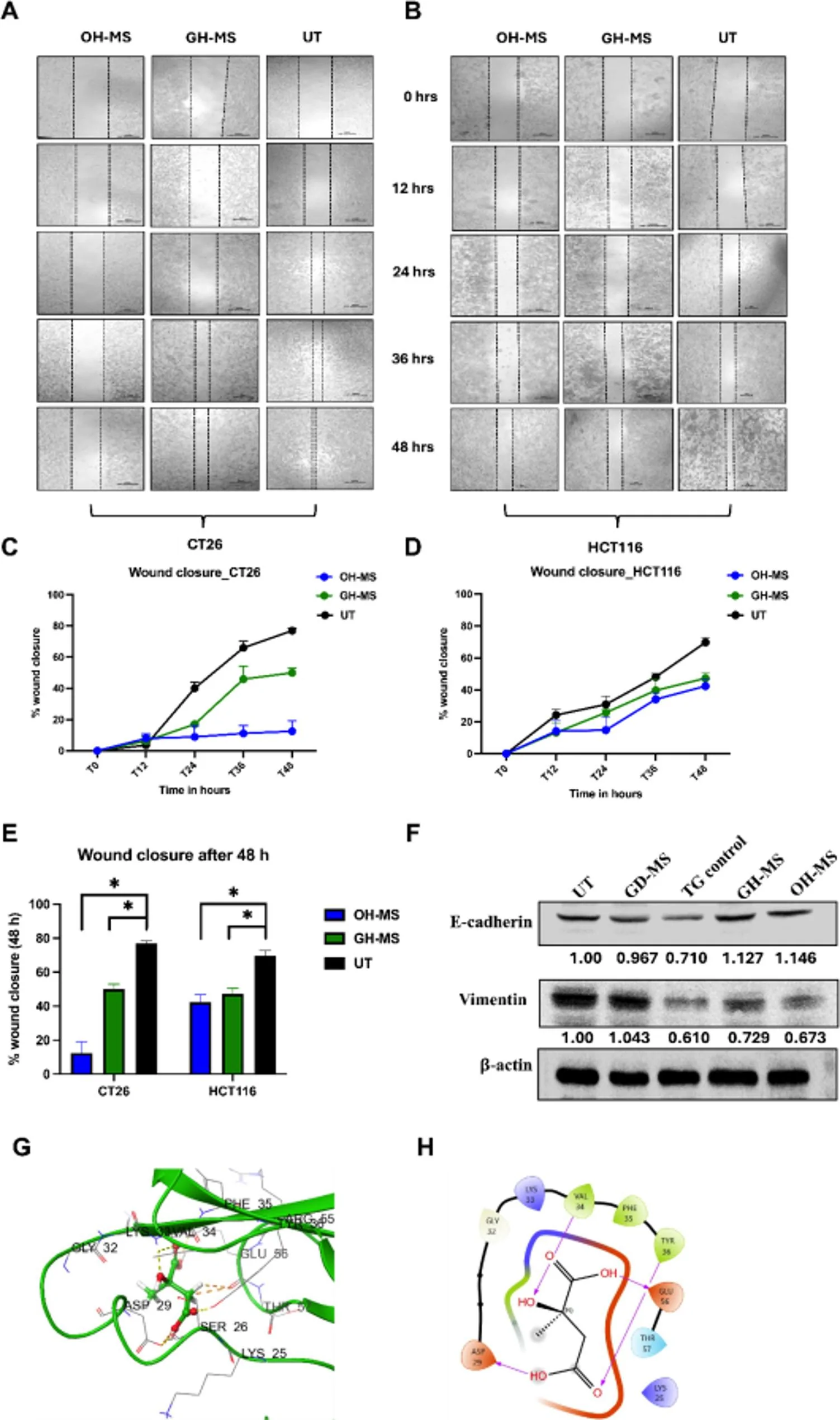
MS inhibits colorectal cancer cell migration and modulates EMT markers. (**A-B**) Representative phase-contrast images of scratch wound-healing assays performed in CT26 (**A**) and HCT116 (**B**) cells treated with OH-MS or GH-MS or left untreated (UT). Images were captured at 0, 12, 24, 36, and 48 h post-scratch. Dashed lines indicate wound boundaries at each time point; (**C-D**) Quantification of wound closure over time in CT26 (C) and HCT116 (D) cells following treatment. Data are presented as percentage wound closure relative to the initial wound area (0 h); (**E**) Endpoint analysis of wound closure at 48 h in CT26 and HCT116 cells. Data represent mean ± SEM from three independent experiments. Statistical significance was assessed using one-way ANOVA followed by Tukey’s multiple comparison test; *p < 0.05; (**F**) Western blot analysis of epithelial-mesenchymal transition (EMT) markers in CT26 cells following treatment with 2.5% of MS (v/v), showing E-cadherin and vimentin expression. β-actin was used as a loading control; **(G)** Predicted 3D binding pose of citramalic acid within the binding site of E-cadherin; and **(H)** 2D interaction diagram depicting the predicted interactions of citramalic acid with amino acid residues within the E-cadherin binding site.

To investigate the effect of MS on colorectal cancer (CRC) cell migration, scratch wound-healing assays were performed using murine CT26 and human HCT116 cell lines. Confluent monolayers were scratched and treated with MS derived from OH or GH, or left untreated (UT). Representative images of CT26 and HCT116 were acquired at 0, 12, 24, 36, and 48 h post-scratch which are shown in Fig. 5A and B respectively. In CT26 cells, untreated controls exhibited rapid wound closure, reaching approximately 75–80% closure at 48 h. In contrast, treatment with GH-MS (2.5%, v/v) significantly reduced migration, with wound closure limited to 45–50%, while OH-MS treatment resulted in a more pronounced inhibition, with closure restricted to 10–15% at 48 h (Fig. 5C). Time-course analysis demonstrated a clear divergence in migration kinetics beginning at 24 h, which further widened at later time points. Similarly, in HCT116 cells, untreated cultures showed progressive wound closure, achieving 65–70% closure at 48 h. Treatment with GH-MS reduced migration to 45–50%, whereas OH-MS treatment limited closure to 40–45% at the final time point (Fig. 5D). Although HCT116 cells displayed overall slower migration compared with CT26 cells, MS treatment consistently suppressed migratory capacity across the entire time course. Quantitative endpoint analysis at 48 h confirmed a statistically significant reduction in wound closure in both CT26 and HCT116 cells following MS treatment compared with untreated controls (Fig. 5E). Statistical significance was assessed using one-way ANOVA followed by Tukey’s multiple comparison test (*p < 0.05). To determine whether the observed inhibition of migration was associated with alterations in epithelial-mesenchymal transition (EMT), expression of key EMT markers was examined by western blotting. MS treatment (2.5%, v/v) resulted in increased expression of the epithelial marker E-cadherin and concomitant downregulation of the mesenchymal marker vimentin, compared with untreated cells (Fig. 5F; densitometry graph is present in Supplementary Fig. S19). These changes indicate a shift toward a more epithelial phenotype, consistent with the observed suppression of migratory behavior. Scratch assays were performed under conditions that did not induce acute cytotoxicity during the migration time course, minimizing confounding effects of reduced viability. Given the observed modulation of EMT markers, particularly the increase in E-cadherin levels following treatment with healthy microbiota-derived MS, we next explored whether representative putative metabolites identified by LC-MS could interact with epithelial adhesion regulators. Molecular docking analysis suggested that citramalic acid, a metabolite enriched in healthy microbiota-derived MS, can occupy a binding pocket within E-cadherin (docking score of −4.811; Fig. 5G-H). The ligand formed multiple hydrogen-bond and electrostatic interactions with residues within the binding cavity, suggesting a potential mechanism by which microbiota-derived metabolites may influence epithelial adhesion pathways. Collectively, these data suggested that MS markedly inhibit CRC cell migration in both murine and human models, an effect that correlates with modulation of EMT-associated markers.

### GH-MS and OH-MS modulate inflammation in CT26 cells

**Fig. 6:**
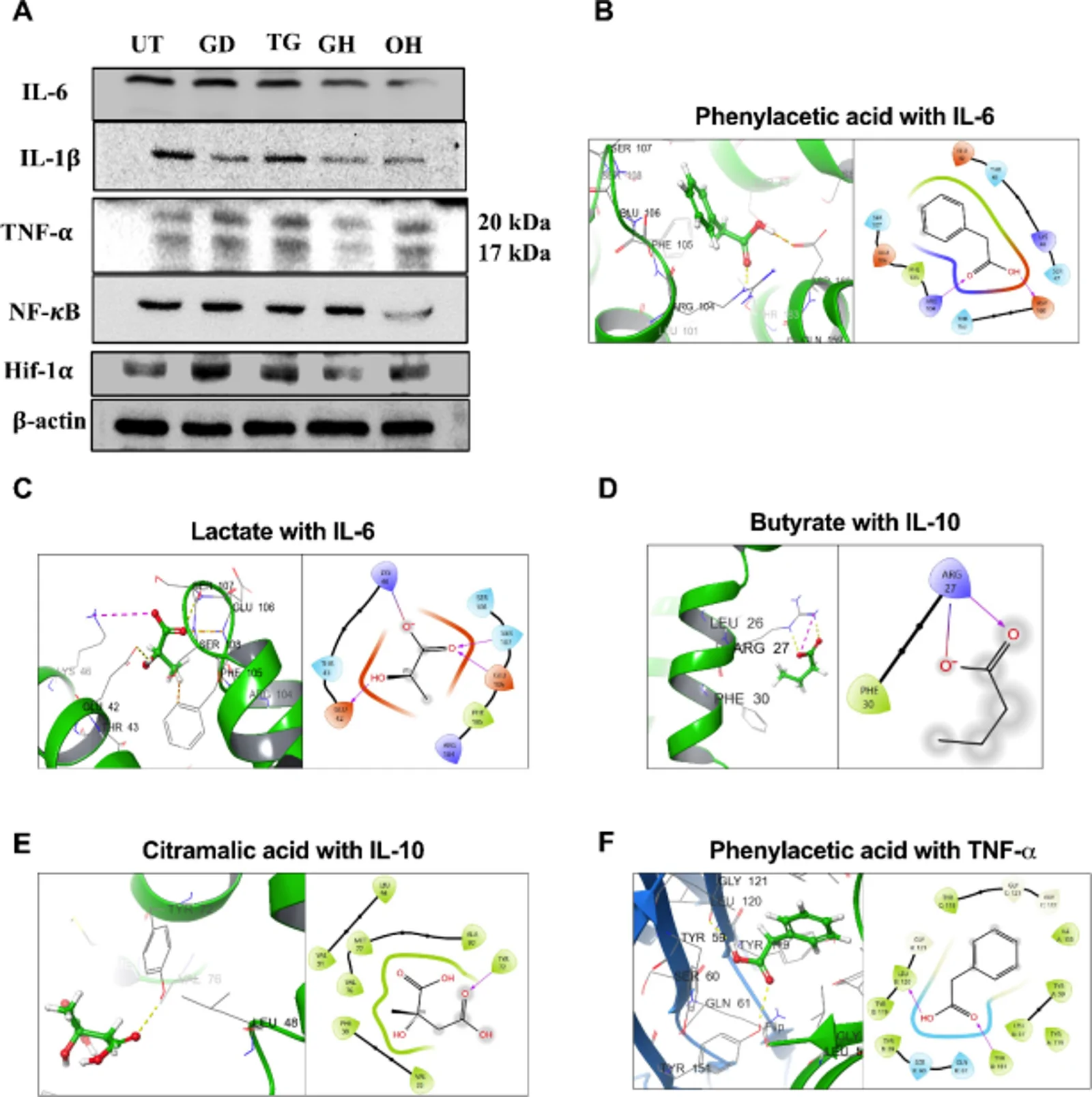
Western blot analysis of inflammatory signaling proteins in CT26 cells following metabolite treatment. CT26 cells were treated with 2.5% microbiota-derived metabolites for 48 h. (**A**) Representative immunoblots of inflammatory and stress-related proteins IL-6, IL-1$\:\beta\:$, TNF-$\:\alpha\:$, NF-$\:\kappa\:$B and HIF-1$\:\alpha\:$. Protein expression levels were normalized to the loading controls and expressed as fold change relative to untreated (UT) controls. Data are presented as mean ± SEM from three independent experiments (*n* = 3). Statistical analysis was performed using one-way ANOVA followed by Dunnett’s multiple comparisons test, compared against UT. Statistical significance is indicated as *p < 0.05, **p < 0.01, ***p < 0.001; (**B**) Representative docking pose illustrating the predicted interaction of phenylacetic acid with human IL-6 (PDB: 1ALU). The ligand is positioned within the shallow pocket where its carboxylate group forms hydrogen-bond and electrostatic interactions with polar residues within the IL-6 binding environment; (**C**) Predicted binding mode of lactate with IL-6, highlighting a polar interaction network involving residues that stabilize the carboxylate and hydroxyl group of lactate within the cytokine pocket; (**D**) Docking interaction of butyrate with IL-10 (PDB: 2H24), showing electrostatic recognition of the ligand carboxylate by Arg27 and additional stabilization through hydrophobic contacts; (**E**) Predicted binding of citramalic acid within an IL-10 surface pocket, where multiple hydrogen bonds and hydrophobic contacts stabilize ligand positioning; (**F**) Docking interaction of phenylacetic acid with TNF-$\:\alpha\:$ (PDB: 9OK6), illustrating ligand accommodation within a trimer-interface pocket stabilized by hydrophobic and polar interactions

Taken together, these results indicate that MS derived from healthy microbial communities suppress CRC migration and modulate EMT markers. Because EMT and tumor motility are closely linked to inflammatory signaling pathways that drive CRC progression, we next examined whether MS also influence key inflammatory mediators in CRC. CT26 cells were treated with dysbiotic gut (GD-MS), thioglycolate control (TG), healthy gut (GH-MS), and oral microbiota (OH-MS) at a concentration of 2.5% v/v, and analyzed by immunoblotting. Compared with untreated controls, GD and TG treatments maintained or modestly increased expression of pro-inflammatory cytokines, including IL-6, IL-1β, and TNF-α (Fig. 6A). In contrast, treatment with GH-MS and OH-MS resulted in a significant reduction in IL-6 and IL-1β expression, with a more pronounced suppressive effect observed for OH-MS. Consistent with these findings, activation of the inflammatory signaling mediator NF-κB was significantly attenuated following GH-MS and OH-MS treatment relative to untreated and dysbiotic conditions. In parallel, expression of the hypoxia-associated transcription factor HIF-1α was significantly reduced in cells treated with GH-MS whereas OH-MS produced a modest modulation, and GD-MS treatment induced elevated HIF-1α levels. Quantitative densitometric analysis showed a coordinated suppression of inflammatory and stress-associated signaling pathways by GH-, and OH-MS (Supplementary Fig. S20). These results indicate that microbiota-derived metabolites exert differential effects on inflammatory and hypoxia-linked signaling in colorectal cancer cells, aligning with their observed impact on epithelial migration and phenotypic plasticity. To further explore potential molecular mechanisms underlying these observations, representative metabolites identified in GH-, and OH-MS were subjected to in silico docking analysis against selected inflammatory cytokines and signaling regulators. Docking simulations suggested that metabolites such as lactate, phenylacetic acid, butyrate and citramalic acid can interact with proteins involved in inflammatory signaling, including IL-6, IL-10 and TNF- α, through hydrogen bonding, electrostatic interactions, and hydrophobic contacts (Fig. 6B-F). detailed interactions are given in supplementary file. Together, the data are consistent with a model in which MS from healthy microbiota constrain CRC-associated microbial fitness and dampen epithelial inflammatory and hypoxia-linked signaling pathways, coinciding with reduced migratory behavior in colorectal cancer cells.

### Probiotic-derived metabolites and host-microbiota conditioned media differentially modulate colorectal cancer cell viability

**Fig. 7:**
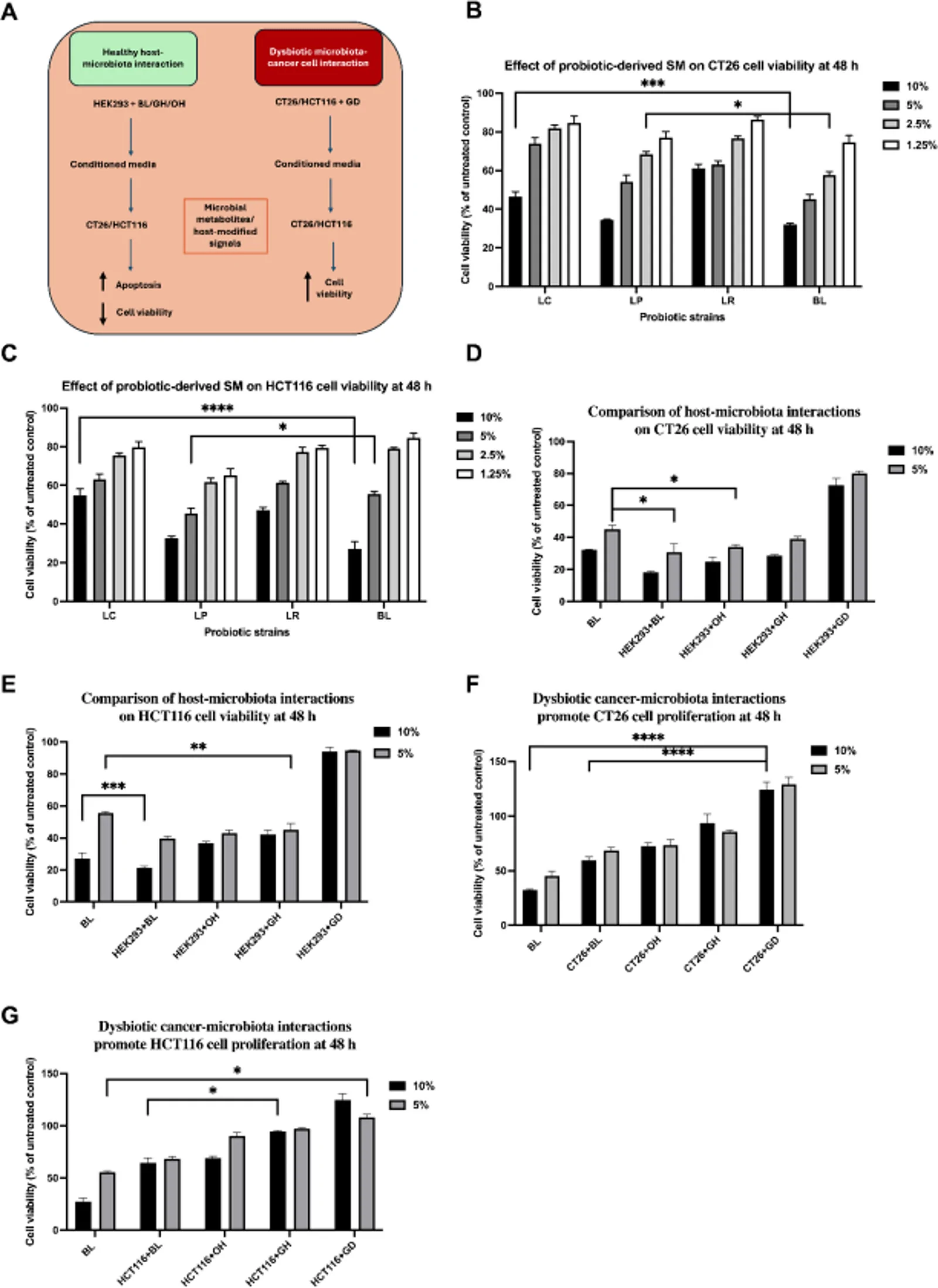
Effects of probiotic-derived metabolites and host-bacteria co-culture conditioned media on colorectal cancer cell viability; (**A**) Schematic representation of the experimental framework illustrating host-microbiota and cancer-microbiota interactions. Conditioned media generated from co-cultures of non-malignant epithelial cells (HEK293) with probiotic strains, or from co-cultures of colorectal cancer cells with bacteria under dysbiotic conditions, were subsequently applied to CT26 and HCT116 cells to assess effects on cell viability and apoptosis; (B-C) Cell viability analysis of murine CT26 (B) and human HCT116 (C) colorectal cancer cells treated with MS derived from probiotic strains *Lactobacillus casei* strain DN-114 001 (LC), *Lactobacillus rhamnosus* strain LR32 (LR), *Lactobacillus plantarum* strain LP-115 (LP) and *Bifidobacterium lactis* strain BB-12 (BL) at indicated concentrations (10%, 5%, 2.5%, and 1.25%) for 48 h; (**D-E**) Effects of conditioned media (CM) obtained from co-cultures of non-malignant epithelial cells (HEK293) and probiotic strains on the viability of CT26 (**D**) and HCT116 (**E**) cells after 48 h of treatment; (**F-G**) Effects of conditioned media (CM) derived from co-cultures of colorectal cancer cells (CT26 or HCT116) and bacteria under dysbiotic conditions on the viability of CT26 (**F**) and HCT116 (**G**) cells at 48 h. Data are presented as mean ± SEM from three independent experiments (*n* = 3). Statistical significance was determined using two-way ANOVA followed by Tukey’s multiple comparisons test. *p* < 0.05, *p < 0.01, **p < 0.001

To evaluate the effects of probiotic-derived MS on colorectal cancer (CRC) cell viability, murine CT26 and human HCT116 cells were treated with metabolites obtained from *Lactobacillus casei* strain DN-114 001 (LC), *Lactobacillus rhamnosus* strain LR32 (LR), *Lactobacillus plantarum* strain LP-115 (LP) and *Bifidobacterium lactis* strain BB-12 (BL) at concentrations ranging from 1.25% to 10%. Cell viability was assessed by MTT assay after 48 h of treatment (Fig. 7B-C). In CT26 cells, treatment with probiotic MS resulted in a dose-dependent reduction in viability, with the most pronounced effects observed at higher concentrations (5–10%). At 48 h, viability was reduced to 32–46% of untreated control depending on the probiotic strain, compared with untreated cells that retained near-baseline viability (Fig. 7B). Similarly, in HCT116 cells, probiotic MS significantly reduced cell viability, with values decreasing to 27–54% of control at higher concentrations (Fig. 7C). Time-course analyses at earlier (24 h) and later (72 h) time points demonstrated consistent trends in both cell lines and are provided in Supplementary Fig. S21.

Next, to assess the impact of host-microbiota interactions on CRC cell viability, conditioned media (CM) derived from co-cultures of healthy non-malignant epithelial cells (HEK293) and probiotic strains were applied to CT26 and HCT116 cells. Treatment with HEK293-healthy bacteria CM significantly reduced CRC cell viability at 48 h compared with baseline controls (Fig. 7D-E). In CT26 cells, viability decreased to 20–30% of untreated control following exposure to CM from healthy host-microbiota co-cultures, whereas HCT116 cells exhibited a reduction to 21–42% under similar conditions.

In contrast, conditioned media derived from co-cultures of CRC cells (CT26 or HCT116) with bacteria associated with dysbiosis, representing dysbiotic cancer-microbiota interactions, significantly increased tumor cell viability (Fig. 7F-G). In CT26 cells, exposure to dysbiotic CM resulted in a marked increase in viability, reaching 120–140% of untreated control at 48 h (Fig. 7F). A similar proliferative effect was observed in HCT116 cells, with viability increasing to 110–130% of control following treatment with dysbiotic CM (Fig. 7G).

Collectively, these data demonstrate that probiotic-derived metabolites and conditioned media from healthy host-microbiota interactions suppress CRC cell viability, whereas conditioned media generated under dysbiotic cancer-microbiota conditions promote tumor cell survival. These findings highlight the context-dependent effects of microbial metabolites and host-microbiota interactions on colorectal cancer cell behavior.

### Microbiota-conditioned media induces apoptosis in colorectal cancer cells

**Fig. 8:**
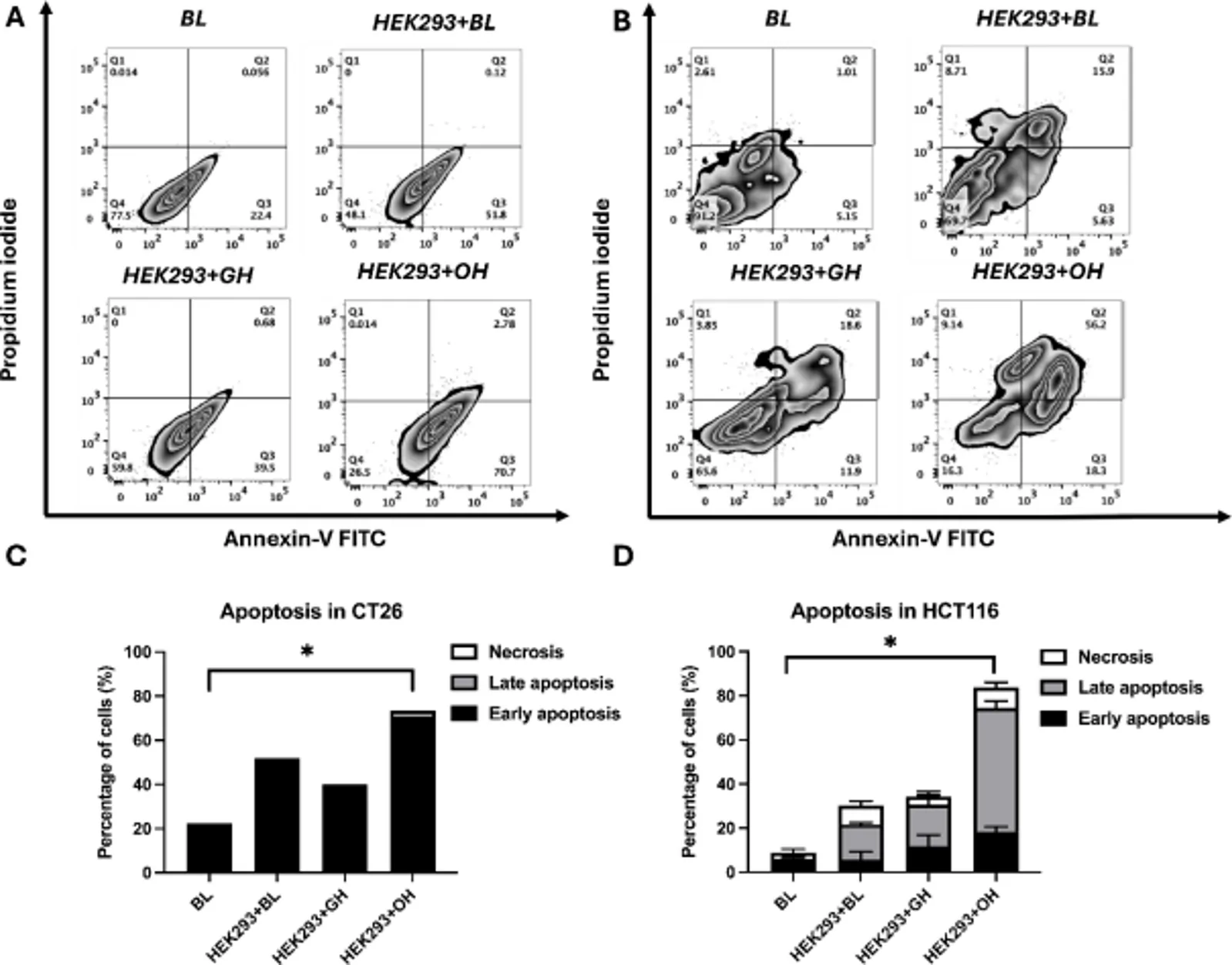
Host-microbiota interaction-derived conditioned media induces apoptosis in colorectal cancer cells. (**A-B**) Representative flow cytometry dot plots showing Annexin V-FITC and propidium iodide (PI) staining of CT26(A) and HCT116 (B) cells following treatment with 2.5% conditioned media (CM) derived from BL-MS, or co-cultures of non-malignant epithelial cells (HEK293) with bacteria (HEK293 + BL, HEK293 + GH, HEK293 + OH). Quadrants represent viable cells (Annexin V⁻/PI⁻), early apoptotic cells (Annexin V⁺/PI⁻), late apoptotic cells (Annexin V⁺/PI⁺), and necrotic cells (Annexin V⁻/PI⁺); (**C-D**) Quantification of apoptosis in CT26 (C) and HCT116 (D) cells following treatment with conditioned media. Data are presented as stacked bar graphs showing the relative proportions of early apoptosis, late apoptosis, and necrosis. Data are presented as mean ± SEM from three independent experiments (*n* = 3). Statistical analysis was performed on total apoptotic cells using unpaired one-way ANOVA followed by Tukey’s multiple comparisons test. Statistical significance is indicated as *p < 0.05

To determine whether HM-CM influences colorectal cancer (CRC) cell survival through induction of apoptosis, Annexin V-FITC/propidium iodide (PI) staining followed by flow cytometry was performed in murine CT26 and human HCT116 cells. Cells were treated with 2.5% (v/v) of BL-MS or from co-cultures of healthy non-malignant epithelial cells (HEK293) with bacteria (HEK293 + BL, HEK293 + GH, and HEK293 + OH). Representative quadrant plots are shown in Fig. 8A-B, and the control plots are provided in Supplementary Fig. S22.

In CT26 cells, treatment with BL-conditioned media resulted in a relatively low basal level of apoptosis, with total apoptotic cells (early + late apoptosis) comprising 20–25% of the population (Fig. 8C). In contrast, CM derived from HEK293-bacteria co-cultures significantly increased apoptosis in CT26 cells. Exposure to HEK293 + BL CM increased total apoptosis to 45–55%, while HEK293 + GH CM induced apoptosis in 35–45% of cells. The most pronounced effect was observed with HEK293 + OH CM, which increased total apoptotic cells to 70–75% of the population.

A similar but more pronounced apoptotic response was observed in HCT116 cells. Treatment with BL-conditioned media resulted in minimal apoptosis, with total apoptotic cells accounting for 5–10% of the population (Fig. 8D). In contrast, CM derived from HEK293-bacteria co-cultures markedly increased apoptosis in HCT116 cells. Total apoptosis increased to 25–30% following HEK293 + BL treatment and 30–35% following HEK293 + GH treatment. Notably, exposure to HEK293 + OH CM induced a robust apoptotic response, with total apoptotic cells reaching 75–80%.

Across both cell lines, the increase in total apoptosis was primarily driven by an expansion of the late apoptotic population, although early apoptotic cells also increased relative to BL-treated controls. Necrotic cell populations remained comparatively low across all conditions, indicating that CM-induced cell death predominantly occurred via apoptotic pathways.

Quantitative analysis of total apoptosis demonstrated a statistically significant increase in apoptotic cells following treatment with CM derived from HEK293-bacteria co-cultures compared with BL-conditioned media alone. Statistical significance was assessed using unpaired one-way ANOVA followed by Tukey’s multiple comparisons test (*p* < 0.05). Collectively, these findings indicate that CM-induced cell death occurred predominantly via apoptotic pathways under the tested conditions.

### In silico analysis of microbiota-derived metabolites with cancer-relevant targets

To gain mechanistic insight into the functional effects of healthy gut- and oral microbiota-derived metabolites, selected compounds identified by LC-MS analysis of GH-MS and OH-MS were subjected to molecular docking against host proteins implicated in epithelial integrity, inflammatory signaling, apoptosis, and stress responses. Targets were selected based on phenotypic pathways modulated in experimental assays, including apoptosis, EMT, and inflammatory signaling. Docking outcomes were summarized as a comparative interaction heat map to visualize relative binding propensities across ligand-protein pairs. The heat map revealed distinct and consistent interaction patterns between several healthy microbiota-derived metabolites and host proteins associated with epithelial homeostasis and apoptotic regulation (Supplementary Fig. S23A). Venn diagram represented many metabolites common among different samples (Supplementary Fig. S23B). Multiple metabolites suggested comparatively stronger predicted interactions with these targets, suggesting potential engagement of pathways relevant to epithelial cell viability and survival. While these docking analyses do not establish direct molecular mechanisms, the observed interaction profiles provide a hypothesis-generating framework that is consistent with the experimentally observed modulation of epithelial responses by GH-MS and OH-MS, supporting a role for healthy microbiota-derived metabolites in shaping host-epithelium interactions.

Semi-quantitative comparison of putatively annotated metabolites further revealed distinct metabolic trends across MS (Supplementary Table S3 and Fig. S24A). To further visualize global metabolite variation among the samples, PCA of LC-QTOF features demonstrated distinct clustering trends across GH-MS, OH-MS and GD-MS, supporting compositional differences in metabolite profiles between microbial origin and disease state (Supplementary Fig. S24B). Relative abundance of different metabolites in different environments have been given in Table 1. PCA-X analysis of LC-QTOF metabolite features generated a 3-component model explaining 99% cumulative variance (R2X(cum) = 99%), with distinct clustering trends observed among GH-MS, OH-MS and GD-MS, supporting differences in global metabolite composition across the three pools. Metabolite environments derived from GH-MS and OH-MS were enriched in short-chain fatty acid (SCFA)-related metabolites, amino acid derivatives, and TCA cycle intermediates, including butyrate, lactate, succinate, and glutamate. In contrast, GD-MS showed relatively higher peak intensity of metabolites associated with aromatic and fatty acid metabolism.

**Table 1:** Putatively annotated metabolites identified by untargeted LC-QTOF-MS in GH-MS, OH-MS and GD-MS. Metabolite annotation was performed based on accurate mass (m/z) matching against available spectral databases. Relative enrichment was assigned according to vendor-generated normalized abundance values and the highest mean signal intensity observed among experimental groups

Metabolite	m/z	Chemical class	Relative enrichment	Major source	Biological relevance	CRC/dysbiosis relevance
Acetic acid	59.01	SCFA	GH/OH	*Bacteroides*	Major SCFA metabolism	Altered in dysbiosis
Propionic acid	73.03	SCFA	GH	*Bacteroidetes*	Anti-inflammatory SCFA	Reduced in CRC
Butyric acid	87.05	SCFA	OH > GH	*Clostridia*	Barrier maintenance	Protective in CRC
Valeric acid	101.06	SCFA	GH	Gut anaerobes	SCFA metabolism	Gut homeostasis
Isobutyric acid	87.04	Branched SCFA	GH	Protein fermentation	Fermentation marker	Dysbiosis-associated
Isovaleric acid	101.06	Branched SCFA	GH	Amino acid fermentation	Gut fermentation	CRC-associated shifts
Lactic acid	89.02	Organic acid	OH	*Lactobacillus*	Fermentation/cross-feeding	Healthy microbiota marker
Formic acid	47.01	Organic acid	OH	Anaerobes	Fermentation by-product	Microbial turnover
Hydroxybutyric acid	104.05	Organic acid	OH	Fermentation	Energy metabolism	Metabolic homeostasis
Succinic acid	118.03	Dicarboxylic acid	OH	*Bacteroides*	Propionate precursor	Elevated in dysbiosis
Malic acid	134.02	Organic acid	OH	Central metabolism	TCA cycle	Carbon metabolism
Malonic acid	104.01	Organic acid	OH	Fatty acid metabolism	Biosynthesis intermediate	Metabolic remodeling
Citramalic acid	148.04	Organic acid	OH	Bacterial fermentation	Fermentation-associated	Gut microbial activity
Citric acid	191.02	TCA metabolite	GH	Host + microbiota	Central metabolism	Dysbiosis-associated
Fumaric acid	117.02	TCA metabolite	GH	Fermentation	Redox metabolism	Microbial metabolic activity
α-Ketoglutarate	145.01	TCA metabolite	GH	Amino acid metabolism	Nitrogen metabolism	Host–microbe signaling
Pyruvic acid	87.01	Keto acid	GH/OH	Glycolysis	Central carbon metabolism	Tumor metabolic shift
Oxaloacetic acid	131.02	TCA intermediate	GH	Central metabolism	Metabolic flux	Gut homeostasis
Aspartic acid	133.04	Amino acid	OH	Amino acid biosynthesis	Nitrogen metabolism	Active microbial turnover
Glutamic acid	147.05	Amino acid	OH	Nitrogen metabolism	Nutrient exchange	Microbial activity
Threonine	119.06	Amino acid	OH	Host + microbial	Mucin synthesis	Barrier support
Alanine	89.05	Amino acid	GH	Fermentation	Carbon metabolism	Common fecal metabolite
Glycine	75.03	Amino acid	GH	Protein metabolism	One-carbon metabolism	Gut homeostasis
Serine	105.04	Amino acid	GH	Amino acid metabolism	Biosynthesis	Metabolic signaling
Valine	117.08	BCAA	GH	Protein fermentation	Energy metabolism	CRC-associated
Leucine	131.09	BCAA	GH	Protein fermentation	Nutrient metabolism	Dysbiosis-associated
Isoleucine	131.09	BCAA	GH	Protein fermentation	Metabolic regulation	Altered in CRC
Methionine	149.05	Sulfur amino acid	GD	Protein metabolism	Methyl metabolism	Tumor metabolism
Lysine	147.11	Amino acid	GH	Protein metabolism	Bacterial growth	Dysbiosis marker
Arginine	175.12	Amino acid	GH	Nitrogen metabolism	Immune signaling	CRC-associated
Phenylacetic acid	136.05	Aromatic acid	OH > GH > GD	Phenylalanine degradation	Aromatic microbial metabolite	CRC-associated dysbiosis
Phenylpropionic acid	150.07	Aromatic acid	GH	Aromatic metabolism	Host–microbial signaling	Dysbiosis marker
Benzoic acid	122.03	Aromatic acid	GH	Aromatic metabolism	Microbial metabolism	Gut microbial signature
p-Cresol	108.06	Phenolic metabolite	GD	Tyrosine metabolism	Epithelial signaling	Dysbiosis-associated
Indole	117.06	Indole metabolite	GH	Tryptophan metabolism	Barrier regulation	Gut homeostasis
Indole-3-acetic acid	175.05	Indole derivative	GH	Tryptophan metabolism	AHR signaling	Protective
Indole-3-lactic acid	205.07	Indole derivative	OH	*Lactobacillus*	Anti-inflammatory	Healthy microbiota
Tryptophan	204.09	Amino acid	GH	Host + microbial	Indole precursor	Immune signaling
Tyramine	138.09	Biogenic amine	GD	Tyrosine fermentation	Microbial signaling	Dysbiosis marker
Putrescine	89.11	Polyamine	GD	Lysine metabolism	Cell proliferation	CRC-associated
Cadaverine	102.12	Polyamine	GD	Amino acid fermentation	Gut microbial amine	Dysbiosis-associated
Spermidine	146.17	Polyamine	GH	Polyamine metabolism	Cell growth	Host–microbial crosstalk
Biotin	245.1	Vitamin	GD	Microbial biosynthesis	Cofactor metabolism	Functional dysbiosis marker
Riboflavin	377.15	Vitamin	GH	Microbial biosynthesis	Redox metabolism	Microbial function
Nicotinic acid	124.04	Vitamin	GH	Vitamin metabolism	NAD metabolism	Metabolic signaling
Pantothenic acid	220.12	Vitamin	GH	Cofactor metabolism	Metabolic homeostasis	Functional marker
Pelargonic acid	157	Fatty acid	GD	Lipid metabolism	Fatty acid metabolism	CRC-associated
Oleic acid	281.25	Fatty acid	GD	Lipid metabolism	Membrane metabolism	Altered in dysbiosis
Gallic acid	170.05	Phenolic compound	GD	Polyphenol metabolism	Antioxidant signaling	CRC-associated shift
Salicylaldehyde-like metabolite	121.9	Aromatic phenolic	GD	Aromatic metabolism	Microbial biotransformation	Dysbiosis-associated

These differences in metabolite composition are consistent with the observed functional effects, where GH-MS and OH-MS were associated with reduced bacterial growth and suppressed CRC cell viability, whereas GD-MS supported tumor-associated phenotypes. While metabolite identities remain putative and no causal relationships are inferred, these patterns suggest that distinct microbiota-derived metabolic environments may contribute to differential regulation of bacterial fitness and host epithelial responses.

## Discussion

Colorectal cancer (CRC) is increasingly recognized as a disease shaped not only by host genetic and epithelial alterations but also by perturbations in the gut microbial ecosystem [45]. In this study, we integrated microbiome profiling with functional assays to delineate how CRC-associated dysbiosis, microbial metabolite-enriched supernatants, and host-microbiota interactions collectively influence bacterial fitness and colorectal cancer cell behavior. Several elegant studies have shown that individual microbiota-derived small molecules can exert potent, tumor-relevant effects. For example, *Fusobacterium nucleatum* derived succinic acid has been implicated in resistance to anti-PD-1 therapy by suppressing CD8⁺ T-cell trafficking and cGAS-IFNβ signaling in CRC models, providing a direct mechanistic link between a single microbial metabolite and therapy outcome [46]. Similarly, microbiome-derived formate was shown to promote tumor invasion and stemness through aryl hydrocarbon receptor signaling, linking a single metabolite to enhanced malignancy [47]. In parallel, numerous reports document the anti-tumor effects of canonical short-chain fatty acids (notably butyrate) on colorectal cancer cell apoptosis, differentiation and immune modulation in vitro and in vivo [48, 49].

While these single-molecule studies establish compelling molecular mechanisms, they do not capture the complex, combinatorial metabolite milieu produced by whole microbial communities. Our results extend these observations by demonstrating that community-derived metabolite mixtures can collectively shape both bacterial ecology and epithelial responses. In silico docking combined with functional assays revealed that MS derived from healthy gut and oral microbiota exert bacteriostatic effects on *Fusobacterium*, while GD-MS promote bacterial expansion. Inclusion of OH-MS in our experimental framework also reflects the growing recognition of an oral-gut microbial axis, where oral microbes and their metabolic products can influence intestinal microbial ecology and contribute to gastrointestinal disease processes [50, 51]. These findings are consistent with the possibility that microbial fitness in CRC is not determined solely by intrinsic bacterial properties but is strongly influenced by the surrounding metabolic environment generated by host-microbiota interactions.

Beyond bacterial growth, MS also impaired *Fusobacterium* interactions with host epithelial cells, significantly reducing bacterial adhesion, while only a modest and non-significant reduction in invasion was observed in vitro. Given the established role of *Fusobacterium* in promoting epithelial inflammation, immune evasion, and tumor progression, attenuation of these early host-microbe interactions may represent a critical mechanism by which healthy microbiota-derived signals limit CRC-associated pathogenicity. Notably, combined exposure of bacteria and host cells to MS resulted in greater reductions in adhesion, whereas effects on invasion remained limited, suggesting that microbial and epithelial responsiveness to metabolites differentially influences distinct stages of host-pathogen interaction.

At the epithelial level, microbiota-derived MS exerted robust inhibitory effects on colorectal cancer cell viability across both 2D monolayer and 3D spheroid models. Importantly, the reduced sensitivity of non-cancerous epithelial cells suggests that microbiota-derived MS may preferentially affect cancer-associated cellular pathways, supporting a context-dependent effect. The more pronounced effects observed in 3D systems underscore the importance of physiologically relevant models for capturing microbiota-driven functional outcomes. In parallel, MS suppressed cancer cell migration and were associated with increased E-cadherin and reduced vimentin expression, indicating modulation of epithelial-mesenchymal transition (EMT)-associated phenotypes. These findings align with growing evidence that microbial metabolites can influence epithelial plasticity and tumor cell behavior beyond direct cytotoxicity.

Host-microbiota interactions further amplified these effects. Conditioned media approaches have previously been used to interrogate microbial and host secreted factors: cancer cell conditioned media can alter bacterial metabolic behaviors, and probiotic culture supernatants or cell-free conditioned media have been reported to inhibit CRC cell proliferation and modulate signaling pathways [52, 53]. However, most prior studies examined *single-strain* supernatants or focused solely on host or bacterial outputs in isolation. By contrast, our host-microbiota conditioned media (HM-CM) experiments directly test the bidirectional consequences of host-bacteria co-culture. We find that HM-CM generated from colorectal cancer cells co-cultured with dysbiotic bacteria promotes *Fusobacterium* growth, whereas HM-CM from HEK293 co-cultured with probiotics exerts a stronger pro-apoptotic, anti-proliferative effect on CRC cells than probiotic-derived metabolites alone. These results indicate that host responses to bacteria (and not bacteria alone) amplify or modulate soluble signals that in turn feed back onto both microbial fitness and epithelial fate. The observation that sterile *F. nucleatum* conditioned medium itself can induce epithelial chemokine release (e.g., CXCL8) further supports the importance of secreted, soluble factors in mediating host-microbe crosstalk [54].

Finally, in silico docking analyses were performed exclusively as hypothesis-generating support to contextualize phenotypic observations. Docking results do not establish molecular mechanisms or direct biochemical interactions and are presented to highlight potential areas for future targeted investigation and experimental validation. Although our findings demonstrate significant effects of microbiota-derived MS in in-vitro and in ex-vivo systems, the absence of in-vivo validation limits the ability to directly extrapolate these effects to physiological CRC progression. Accordingly, the study establishes associations between metabolite environments and biological responses but does not establish direct causal mechanisms at the molecular level.

Several limitations of the present study should be acknowledged. Microbiome sequencing was performed on pooled fecal samples from each experimental group, which precluded analysis of inter-individual variability and limited statistical comparison of diversity metrics. Consequently, microbiome data were interpreted primarily as ecological context for downstream functional experiments. Importantly, the main conclusions of this study are supported by functional assays evaluating bacterial growth, host-microbe interactions, and epithelial responses to microbiota-derived metabolite environments. Although the isolate was characterized using selective culturing, antibiotic profiling, morphological characterization and biochemical assays consistent with *Fusobacterium* sp., definitive taxonomic identification using isolate-level 16 S rRNA sequencing or whole-genome sequencing was not performed. Therefore, the isolate is referred to as a putative *Fusobacterium* sp., and this represents a limitation of the study. We acknowledge that, in the absence of isolate-level molecular identification, the identity of the organism remains provisional and should be interpreted accordingly. Additionally, use of HEK293 as a non-malignant epithelial model for generating host-microbiota conditioned media is another limitation of this study. They were used as a standardized epithelial system to evaluate soluble factors generated during host-microbiota interactions under controlled conditions. Nevertheless, epithelial responses in the intestinal mucosa may differ from those observed in HEK293 cells. Future studies using intestinal epithelial models such as primary intestinal organoids will be important to further validate and extend host-microbiota interaction mechanisms identified in this study. Finally, although our data indicate that complex MS mixtures influence bacterial fitness and epithelial responses, the specific metabolites responsible for these effects remain to be identified. Future studies should fractionate metabolite preparations and perform targeted add-back or depletion experiments to determine which metabolite classes, such as SCFAs, organic acids, bile acid derivatives or microbial cofactors, are responsible for the bacteriostatic and host-modulatory phenotypes observed here.

Our findings indicate functional effects of microbiota-derived metabolite environments on bacterial behavior and epithelial responses. However, these results should be interpreted as associative rather than mechanistic, as the MS represent complex mixtures and individual bioactive components were not isolated. Thus, while distinct metabolite landscapes correlate with and modulate observed phenotypes, the specific metabolites responsible for these effects remain to be identified.

## Conclusion

In summary, this study provides evidence suggesting that CRC-associated dysbiosis represents a functionally distinct microbial state associated with altered bacterial fitness and epithelial responses. By integrating microbiome profiling with functional assays, we show that microbiota-derived metabolite environments differentially influence the growth and host interaction capacity of CRC-associated bacteria, particularly *Fusobacterium*. Collectively, these findings highlight microbiota-derived metabolites may function as higher-order regulators of host-microbe interactions during CRC-associated dysbiosis. Rather than acting through single dominant molecules, complex metabolite repertoires produced by microbial communities appear to contribute to ecological constraints on pathobiont expansion while modulating epithelial signaling networks linked to inflammation, hypoxia and tumor progression. These results provide a functional framework for understanding how microbial metabolic environments shape tumor-associated microbial behavior and host responses. Future studies aimed at identifying the specific metabolites and host signaling pathways responsible for these effects may reveal new opportunities for microbiome-based strategies to modulate tumor-associated microbial ecology and epithelial responses in colorectal cancer.

## Supplementary Information

Supplementary Material 1

Supplementary Material 2

## Data Availability

All data supporting this study’s findings are included within the article (and any supplementary files).

## Abbreviations

AUC: Area Under the Curve
CRC: Colorectal Cancer
GD: Gut Dysbiosis
GH: Healthy Gut
HM-CM: Host-Microbiota Conditioned Media
HIF-1α: Hypoxia-Inducible Factor 1 Alpha
IL: Interleukin
LC-MS: Liquid Chromatography-Mass Spectrometry
MOI: Multiplicity of Infection
MS: Metabolite-Enriched Supernatant
NF-κB: Nuclear Factor kappa B
OH: Oral Healthy
PCoA: Principal Coordinates Analysis
SEM: Standard Error of Mean
UT: Untreated
