# Supplementary material for: Microbiota-derived metabolite landscapes modulate Fusobacterium fitness and colorectal cancer cell behaviour
Source: Gut Pathog. 2026 Jul 16;18:81. doi: 10.1186/s13099-026-00859-9 (PMC13660147; doi:10.1186/s13099-026-00859-9)
Supplement: Supplementary file 1 [file 13099_2026_859_MOESM1_ESM.pdf]

SUPPLEMENTARY SECTION S1: MATERIALS AND METHODS

1. **Materials and reagents**

Dulbecco's Modified Eagle Medium (DMEM; Cat. No. 12100046), fetal bovine serum (FBS; Cat. No. 10270106), penicillin-streptomycin solution (Cat. No. 15140122), and trypsin-EDTA (Cat. No. 15400054) were obtained from Gibco (Thermo Fisher Scientific, Waltham, MA, USA). Dimethyl sulfoxide (DMSO; Cat. No. TC185-100ML; cell culture grade), Luria-Bertani (LB) broth (Cat. No. M1245-500G), bovine serum albumin (BSA; Cat. No. MB083; molecular biology grade), and vancomycin (Cat. No. MD063), Enriched thioglycolate broth (Cat. No. M738) were purchased from HiMedia Laboratories (Mumbai, India). MTT reagent (Cat. No. 33611) and agarose (Cat. No. 9012-36-6) were obtained from SRL (Sisco Research Laboratories)(Mumbai, India). Protease inhibitor cocktail (Cat. No. P8340-1ML) was purchased from Sigma-Aldrich (St. Louis, MO, USA). Enhanced chemiluminescence (ECL) detection reagents (Cat. No. 32106), Annexin V-FITC/propidium iodide apoptosis detection kit (Cat. No. V13242), and metronidazole (Cat. No. 210340050) were obtained from Thermo Fisher Scientific (Waltham, MA, USA). All reagents were of analytical or cell culture grade and were used according to the manufacturers' instructions.

2. **16S rRNA gene sequencing & microbiota analysis**

Genomic DNA was extracted from the mouse fecal samples using Xploregen stool DNA extraction kit (Xploregen, India) according to the manufacturer's protocol, which includes mechanical disruption in bead tubes followed by chemical lysis, removal of proteins and RNA, binding of DNA to silica membrane, sequential washing and elution in Tris-HCl buffer. DNA quantity and purity were assessed using a NanoDrop spectrophotometer, and only samples with A260/A280 ratios of approximately 1.8-2.0 and intact high-molecular-weight bands on agarose gel electrophoresis were used for amplification. The V3-V4 region of the bacterial 16S rRNA gene was amplified using universal primers 341F (5'-CCTACGGGNGGCWGCAG-3') and 806R (5'-GACTACHVGGGTATCTAATCC-3'). Each PCR reaction contained 40 ng of template DNA, 10 pM of each primer and a high-fidelity Taq master mix. Cycling conditions were: initial denaturation at 95 °C for 3 min, followed by 25 cycles of 95 °C for 15 s, 60 °C for 15 s, and 72 °C for 2 min, with a final extension at 72 °C for 10 min. Amplicon size and specificity were confirmed by agarose gel electrophoresis, and PCR products were purified using AMPure XP beads (Beckman Coulter, USA). Purified amplicons were subjected to a second PCR to attach dual illumina barcoded adapters, using eight additional cycles under the

manufacturer's recommended conditions. Libraries were again purified with AMPure beads, quantified using the Qubit dsDNA High Sensitivity assay (Thermo Fisher Scientific, USA), normalized and pooled equimolarly. Paired-end sequencing (2 x 300 bp) was performed on an Illumina MiSeq platform using the MiSeq v3 chemistry. Raw base call (bcl) files were demultiplexed into sample-wise FASTQ files using the MiSeq software. Read quality was assessed with FastQC (v0.11.9) and MultiQC (v1.10.1), and low-quality reads and adapter sequences were filtered using the in-house metagenomics pipeline provided by the sequencing facility. High-quality reads were processed to generate an operational taxonomic unit (OTU) table using the standard 16S workflows (merged paired reads, quality trimming, chimera removal and clustering), and taxonomy was assigned against the NCBI 16S rRNA database. The resulting OTU table was used for downstream diversity and compositional analyses. Normalization was performed using rarefaction to the minimum library size across samples followed by total sum scaling (TSS), as implemented in the standardized analysis workflow. Alpha diversity metrices, including Chao1, Shannon, Simpson and Fisher indices, were calculated using filtered OUT tables. Beta-diversity was assessed based on Bray-Curtis distances with principal coordinates analysis (PCoA), and visualization of relative taxonomic abundances and differentially abundant taxa between GH and GD groups. Taxonomic composition was analyzed at phylum, class, order, family, genus, and species levels. Heatmaps were constructed using the top 50 genera based on average abundance, applying unit variance scaling and correlation-based clustering. Core microbiome analysis was performed using a prevalence threshold of 35% and a relative abundance cutoff of 1%. Predicted functional profiling was performed using PICRUSt2 to infer KEGG orthologs and pathway-level functions from OTU-based taxonomic data. Functional predictions were used to identify potential pathway-level trends associated with microbial compositional differences. Given the inherent limitations of predictive metagenomic inference from 16S rRNA gene data, functional predictions were interpreted as exploratory and were used solely to inform downstream experimental and mechanistic analyses.

3. Cell viability assay

Cell viability was assessed using the MTT assay to determine the cytotoxic effects of microbiota- and host-microbiota-derived metabolite preparations. Murine colorectal carcinoma cells CT26 and human colorectal carcinoma cells HCT116 were seeded at a density of 7×10^3 cells per well in 96-well plates in complete DMEM and allowed to adhere overnight under

standard culture conditions (37 °C, 5% CO_2). The following day, culture medium was replaced with fresh medium containing GH-, GD-, OH-SM, probiotic-derived metabolites, or host-microbiota conditioned media at specified concentrations (v/v). Control wells received equivalent volumes of corresponding CM1X media. Cells were incubated for 24, 48, and 72 h**.** At each time point, 20 µL of MTT solution (5 mg/mL in PBS) was added to each well and plates were incubated for 3 h at 37 °C to allow formation of formazan crystals. The medium was then carefully removed, and the crystals were dissolved in 50 µL dimethyl sulfoxide (DMSO). Absorbance was measured at 570 nm using a microplate reader. Cell viability was expressed as a percentage relative to control cells. Each condition was tested in triplicate wells per experiment, and experiments were repeated independently at least three times.

4. 3D Spheroid formation and viability assay

Three-dimensional (3D) tumor spheroids were generated using a hybrid hanging drop-agarose overlay method to better mimic in vivo tumor architecture. Colorectal cancer cells CT26 and HCT116 were suspended at a density of 5×10^3 cells in 20 µL complete DMEM. Drops were carefully placed on the inner surface of a 96-well plate lid (hanging drop method). Wells of the plate base were filled with sterile PBS to maintain humidity and prevent evaporation. Plates were incubated under standard culture conditions (37 °C, 5% CO_2) for 48 h to allow formation of initial compact spheroids. After 2 days, the hanging drops containing spheroids were carefully transferred into 96-well plates pre-coated with 0.6% agarose to prevent cell attachment. Plates were further incubated for an additional 48 h to allow maturation of spheroids. On day 4 of spheroid formation, culture medium was supplemented with fresh medium containing GH-, GD-, OH-SM, probiotic-derived metabolites, or HM-CM at 5% (v/v) for 48 h. Following treatment, cell viability within spheroids was assessed using the MTT assay. MTT was added to each well at a final concentration of 0.5 mg/mL and incubated for 4 h at 37 °C. Plates were then centrifuged to facilitate formazan crystal sedimentation. The medium was carefully removed without disturbing spheroids, and 50 µL DMSO was added to dissolve formazan crystals. Absorbance was measured at 570 nm using a microplate reader. Spheroid viability was expressed relative to control spheroids. Experiments were performed in triplicate and repeated independently at least three times.

5. Spheroid-forming capacity assay (tumorigenic potential)

To evaluate the effect of metabolites on the tumorigenic and spheroid-forming capacity of colorectal cancer cells, a post-treatment spheroid formation assay was performed. Colorectal cancer cells CT26 and HCT116 were first seeded at a density of 1×10^5 cells per well in 12-well plates and allowed to adhere overnight. Cells were then treated with GH-SM and OH-SM at the concentration of 2.5% (v/v) for 48 h. Following treatment, both attached and floating cells were collected, trypsinized where required, and resuspended as a single-cell suspension. Cells were then subjected to spheroid formation using the hanging drop method. Briefly, 20 μL drops containing 5000 viable cells were placed on the inner surface of a 96-well plate lid, and PBS was added to the bottom wells to maintain humidity. Plates were incubated for 48 h to allow formation of initial spheroids. Spheroids were subsequently transferred to 96-well plates coated with 0.6% agarose and cultured under non-adherent conditions. Spheroid formation and growth were monitored by phase-contrast microscopy, and images were captured on days 2, 4, and 7 following transfer to agarose-coated plates. Spheroid size and morphology were compared between treated and control groups to assess the effect of metabolite exposure on spheroid-forming efficiency.

6. Scratch assay

HCT116 and CT26 cells were seeded into 12-well plates and grown to confluence. The following day, cells were treated with fresh medium containing SM at a concentration of 2.5% (v/v) for 24 hours, followed by a uniform scratch using a sterile 200 μL pipette tip. Cells were given a gentle PBS wash to remove detached cells and supplemented with fresh media. Images were captured at 0, 24 and 48 hours using an inverted microscope. Wound area was quantified using ImageJ and migration was expressed as percentage wound closure relative to time 0.

7. Western Blot analysis

Murine colorectal carcinoma cells CT26 were seeded at a density of 2×10^5 cells per well in 6-well plates and allowed to adhere overnight. Cells were treated with 2.5% (v/v) of GH-SM, GD-SM, OH-SM, and TG (thioglycolate media) for 24 h. Following treatment, cells were washed with cold PBS and lysed using cell lysis buffer supplemented with protease inhibitor cocktail. Lysates were collected and clarified by centrifugation to remove debris. Protein concentration was determined using the Bradford assay. Equal amounts of protein (30 μg per sample) were resolved by SDS-PAGE and transferred onto PVDF membranes. Membranes were blocked with 5% bovine serum albumin (BSA) in TBST for 1 h at room temperature and

incubated overnight at 4 °C with primary antibodies against proteins related to epithelial-mesenchymal transition (EMT), hypoxia, inflammation, and apoptosis pathways. After washing with TBST, membranes were incubated with appropriate HRP-conjugated secondary antibodies for 1 h at room temperature. Protein bands were visualized using enhanced chemiluminescence (ECL) reagents and imaged using a chemiluminescence detection system. Band intensities were quantified using ImageJ software and normalized to β-actin. Experiments were performed in at least three independent biological replicates. The same antibody panel (listed in Supplementary Table S1) was used across specified treatment groups.

Target	Host	Supplier	Catalog No.	Dilution
**E-cadherin**	Rabbit	ProteinTech	20874-1-AP	1:1000
**Vimentin**	Rabbit	CST	D21H3	1:1000
**Bax**	Rabbit	CST	D2E11	1:1000
**Bcl-2**	Rabbit	ProteinTech	12789-1-AP	1:1000
**IL-6**	Rabbit	ProteinTech	21865-1-AP	1:1000
**IL-1β**	Rabbit	ProteinTech	98142-1-RR	1:1000
**TNF-α**	Rabbit	CST	D1G2	1:1000
**NF-κB**	Rabbit	CST	D14E12	1:1000
**HIF-1α**	Rabbit	ProteinTech	20960-1-AP	1:1000
**β-actin**	Rabbit	Invitrogen	PA1-183	1:2000
**HRP-conjugated anti-rabbit secondary antibody**	Goat	ProteinTech	SA00001-2	1:10000

Supplementary Table S1. *List of primary and secondary antibodies used for Western blot analysis in this study, including target proteins, host species, suppliers, catalogue numbers and working dilutions.*

8. Apoptosis assay (Flow Cytometry)

Colorectal cancer cells CT26 and HCT116 were seeded in 6-well plates and allowed to adhere overnight. Cells were treated with 2.5% (v/v) of HEK293-GH and HEK293-OH conditioned media for 48 h. Following treatment, both adherent and floating cells were collected to avoid loss of apoptotic cells. Cells were washed twice with cold PBS and resuspended in binding buffer. Annexin V-FITC and PI were added according to the manufacturer's protocol, and

samples were incubated for 15 min at room temperature in the dark. Stained cells were analyzed using a flow cytometer (BD FACS). A minimum of 10,000 events per sample were acquired. Data were analyzed using FlowJo v10 software to quantify viable cells (Annexin V^-/PI^-), early apoptotic cells (Annexin V^+/PI^-), late apoptotic/necrotic cells (Annexin V^+/PI^+), and necrotic cells (Annexin V^-/PI^+). Results were expressed as the percentage of total apoptotic cells (early + late apoptosis). Experiments were performed in triplicate and repeated independently at least three times.

9. Untargeted metabolomic profiling of microbiota-derived metabolites

Untargeted liquid chromatography-mass spectrometry (LC-MS) analysis was performed to profile metabolites present in sterile, cell-free culture supernatants derived from healthy gut (GH-MS), oral microbiota (OH-MS), and colorectal cancer gut microbiota (GD-MS). Supernatants were clarified and subjected to LC-MS analysis using standard reverse-phase chromatographic separation coupled to high-resolution mass spectrometry. Acquired spectra were processed for peak detection, alignment, and normalization, and metabolites were annotated based on accurate mass matching against public metabolite databases. Metabolite annotation was considered putative and based on accurate mass matching in accordance with metabolomics reporting standards. Comparative analysis was performed to identify differentially abundant metabolites across GH-MS, OH-MS, and GD-MS samples. Metabolites of interest were selected based on relative abundance, differential enrichment between groups, and reported biological relevance to host signaling, inflammation, apoptosis, and cancer-associated pathways. Metabolite selection was used to generate biologically informed hypotheses rather than definitive pathway quantification. The identified metabolites were subsequently used as ligands for molecular docking analyses.

10. In silico host target interaction analysis of microbiota-derived metabolites

This section provides extended computational methodology corresponding to the docking studies described in the main Methods.

10.1 Ligand Preparation

Computational studies were performed using a curated ligand library prepared with the LigPrep module (Schrödinger Release 2025-2, Schrödinger, LLC, New York, USA). Ligands were processed using the OPLS_2005 force field, and all possible ionization states were generated at pH 7.0 ± 2.0 using Epik, while retaining the original chemical state of each ligand.

Stereochemistry was preserved where specified, and energetically minimized three-dimensional conformations were generated for subsequent docking studies.

10.2 Protein Preparation

Crystal structures of the target proteins - PDB: 6O0K | BCL-2, PDB:5IWG HDAC2, PDB: 1T69 HDAC8, PDB: 2O72 human E-cadherin, PDB: 1ALU human interleukin-6 and PDB: 2H24 human IL-10 were retrieved from the RCSB Protein Data Bank. Protein preparation was carried out using the Protein Preparation Wizard in Maestro 14.4 (Schrödinger 2025-2). The preparation protocol included bond order assignment, addition of hydrogen atoms, removal of crystallographic water molecules, optimization of hydrogen-bonding networks, and restrained energy minimization using the OPLS_2005 force field. All ionizable residues were adjusted to pH 7.0, and structures were converted from 2D to optimized 3D conformations.

10.3 Active Site Prediction

The active sites of PDB: 6O0K | BCL-2, PDB:5IWG HDAC2, PDB: 1T69 HDAC8, PDB: 2O72 human E-cadherin, PDB: 1ALU human interleukin-6, and PDB: 2H24 human IL-10 were identified using the Sitemap module or by generating receptor grids based on co-crystallized ligands, where available. Sitemap analysis was performed to locate energetically favorable ligand-binding regions on the protein surface by evaluating pocket size, enclosure, hydrophobicity, and hydrogen-bonding capability. Grid boxes were generated around the predicted binding sites using Maestro 14.4 (Schrödinger 2025-2).

10.4 Glide Docking

Prepared ligands and reference co-crystallized compounds were docked into the active sites of the target proteins using the Glide docking module in Maestro 14.4 (Schrödinger 2025-2). Docking was performed using the Standard Precision (SP) mode with flexible ligand sampling. Default docking parameters recommended by Schrödinger were applied. Binding poses were ranked based on Glide Score (G-score) and docking score, and the most energetically favorable conformations were selected for interaction analysis.
