# Supplementary material for: Microbiota-derived metabolite landscapes modulate Fusobacterium fitness and colorectal cancer cell behaviour
Source: Gut Pathog. 2026 Jul 16;18:81. doi: 10.1186/s13099-026-00859-9 (PMC13660147; doi:10.1186/s13099-026-00859-9)
Supplement: Supplementary file 2 [file 13099_2026_859_MOESM2_ESM.pdf]

1. **CRC-associated dysbiosis is characterized by a pronounced loss of gut microbial diversity**

GC content and sequencing quality: The GC content of the sequenced libraries was highly comparable between normal and dysbiosis-associated mouse samples, with values of ~55% across groups (**Supplementary Fig. S1 and Table S1**). The uniform GC distribution indicates consistent amplification of the V3-V4 region and minimal sequencing bias, confirming overall data quality and suitability for downstream microbiome analyses.

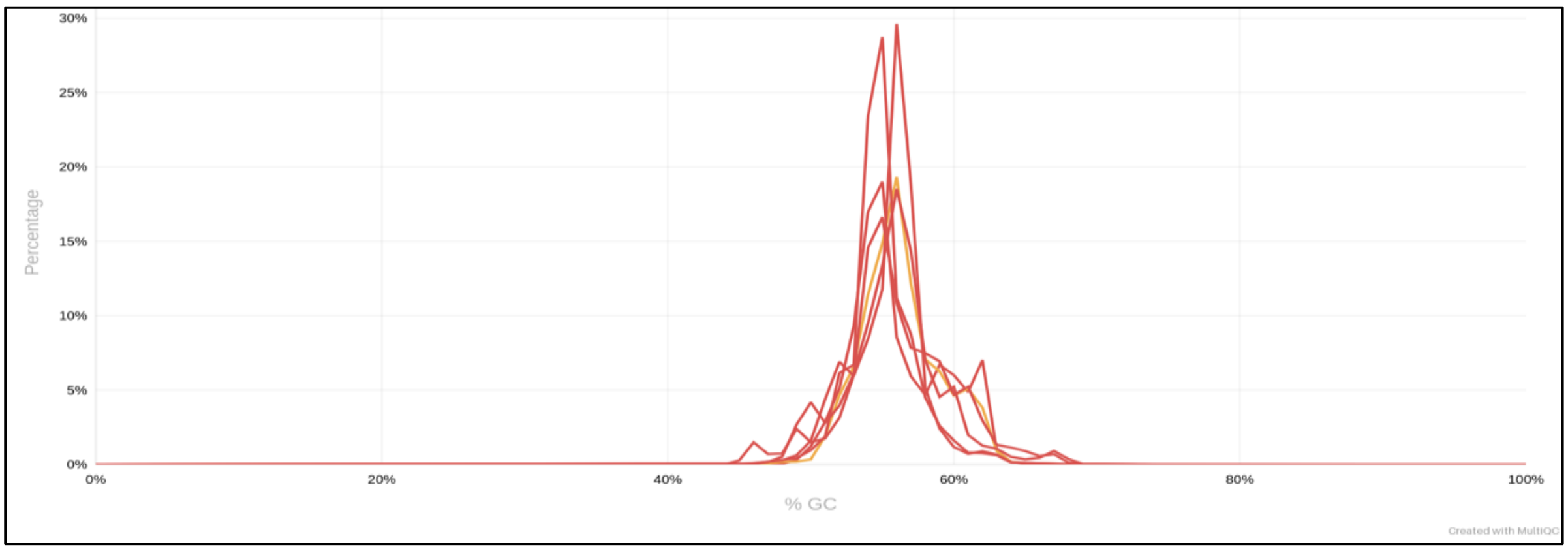

Figure S1. *Per-sequence GC content distribution generated by FastQC for 16S rRNA amplicon sequencing libraries. GC profiles show consistent distribution across libraries, indicating no major GC bias.*

SL.no	Sample Name	%GC	Total Sequences(million reads)
1	CRC-ASSOCIATED DYSBIOSIS MICE	55.5	0.106
2	NORMALMICE	55.0	0.109

Table S1 GC-content of different samples.

Library size and sequencing depth: Total sequencing output revealed comparable library sizes between groups, with an average of approximately 0.10 million reads per sample (**Supplementary Fig. S2**). The similarity in read depth across samples minimizes technical variability and ensures that observed differences in microbial composition are reflective of biological variation rather than sequencing artifacts.

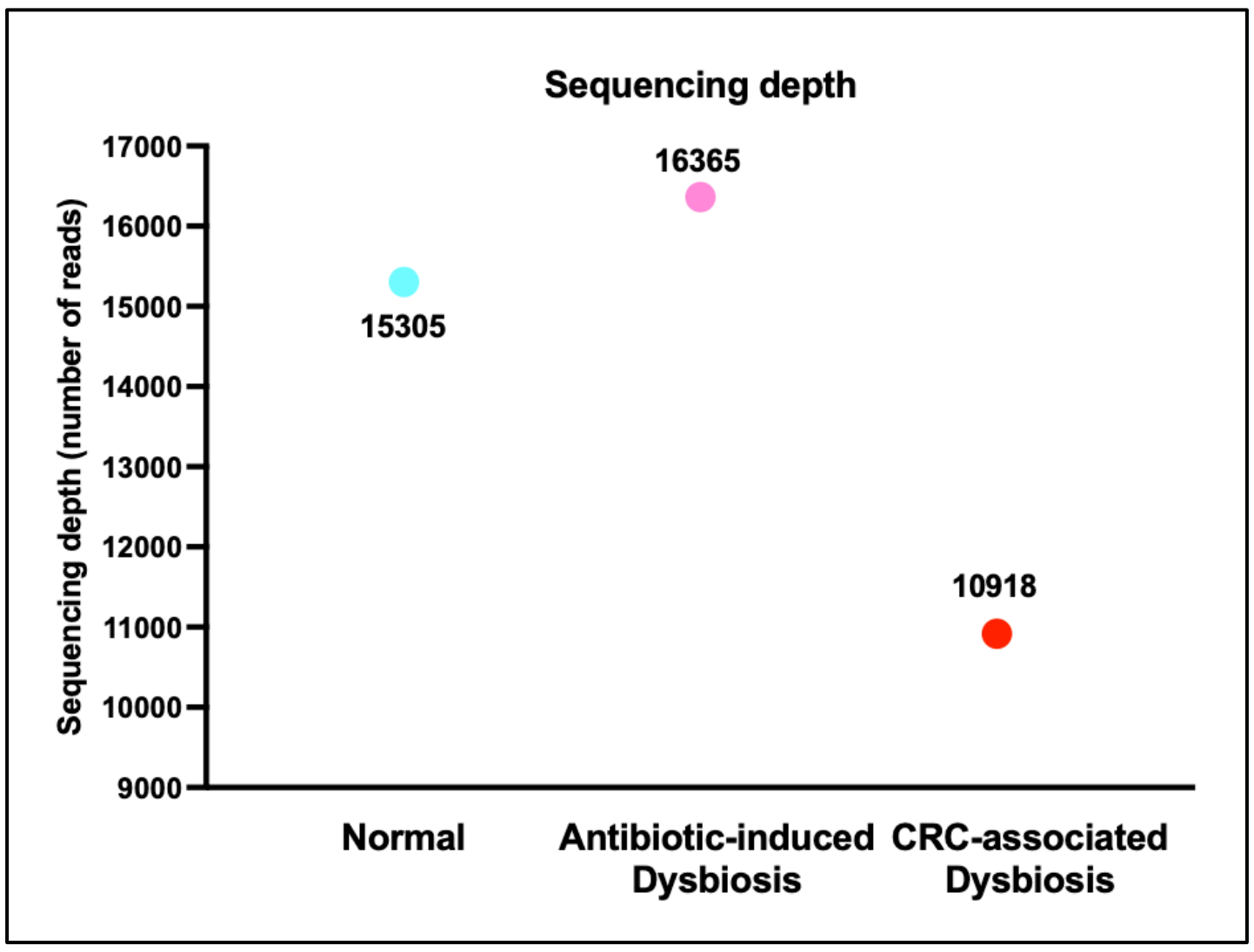

Figure S2. Sequencing depth of pooled microbiome libraries from normal, antibiotic-induced dysbiosis and CRC-associated dysbiosis groups. *Values indicate total number of high quality reads obtained after quality filtering for each pooled sample.*

Core microbiome analysis: Core microbiome analysis was performed using a relative abundance detection threshold of 1% and a prevalence cutoff of 35%, as provided by the sequencing analysis pipeline. Under these criteria, the normal microbiome profile exhibited a larger and more stable set of shared taxa, whereas dysbiosis-associated samples showed a reduced number of consistently detected genera (Supplementary Fig. S3). This reduction in shared microbial members is indicative of disrupted microbial stability and loss of conserved gut-associated taxa under dysbiotic conditions.

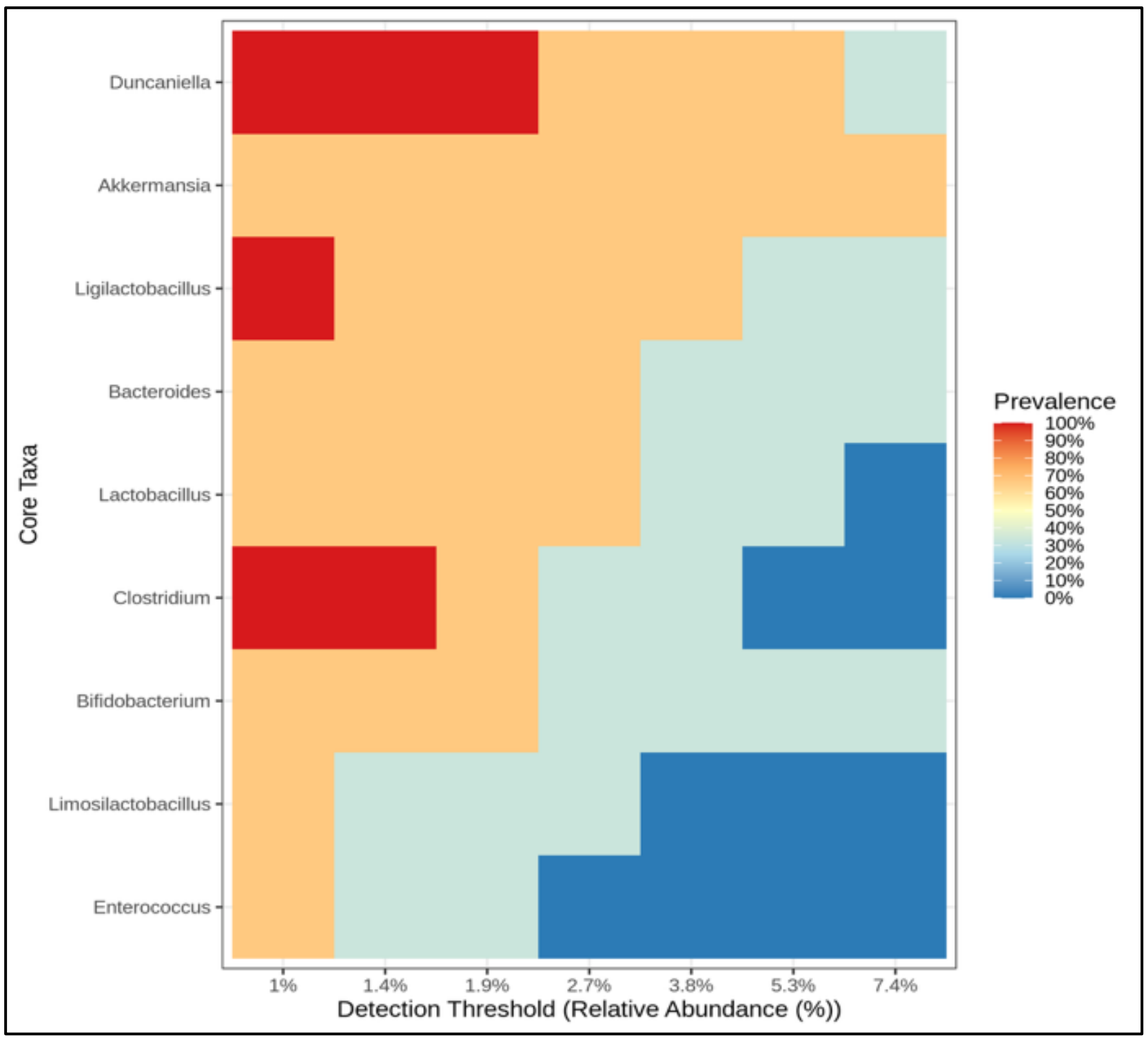

Figure S3: Core microbiome analysis

Heatmap of dominant genera: A heatmap based on the top 50 genera ranked by mean relative abundance was generated to visualize compositional differences between groups (Supplementary Fig. S4). Hierarchical clustering revealed distinct genus-level microbial signatures in normal and dysbiosis-associated samples. Dysbiosis samples were characterized by increased abundance of CRC-associated genera, including *Fusobacterium*, while normal samples showed higher representation of commensal gut bacteria. The clustering pattern further demonstrates clear separation between experimental groups, supporting disease-associated restructuring of the gut microbiota.

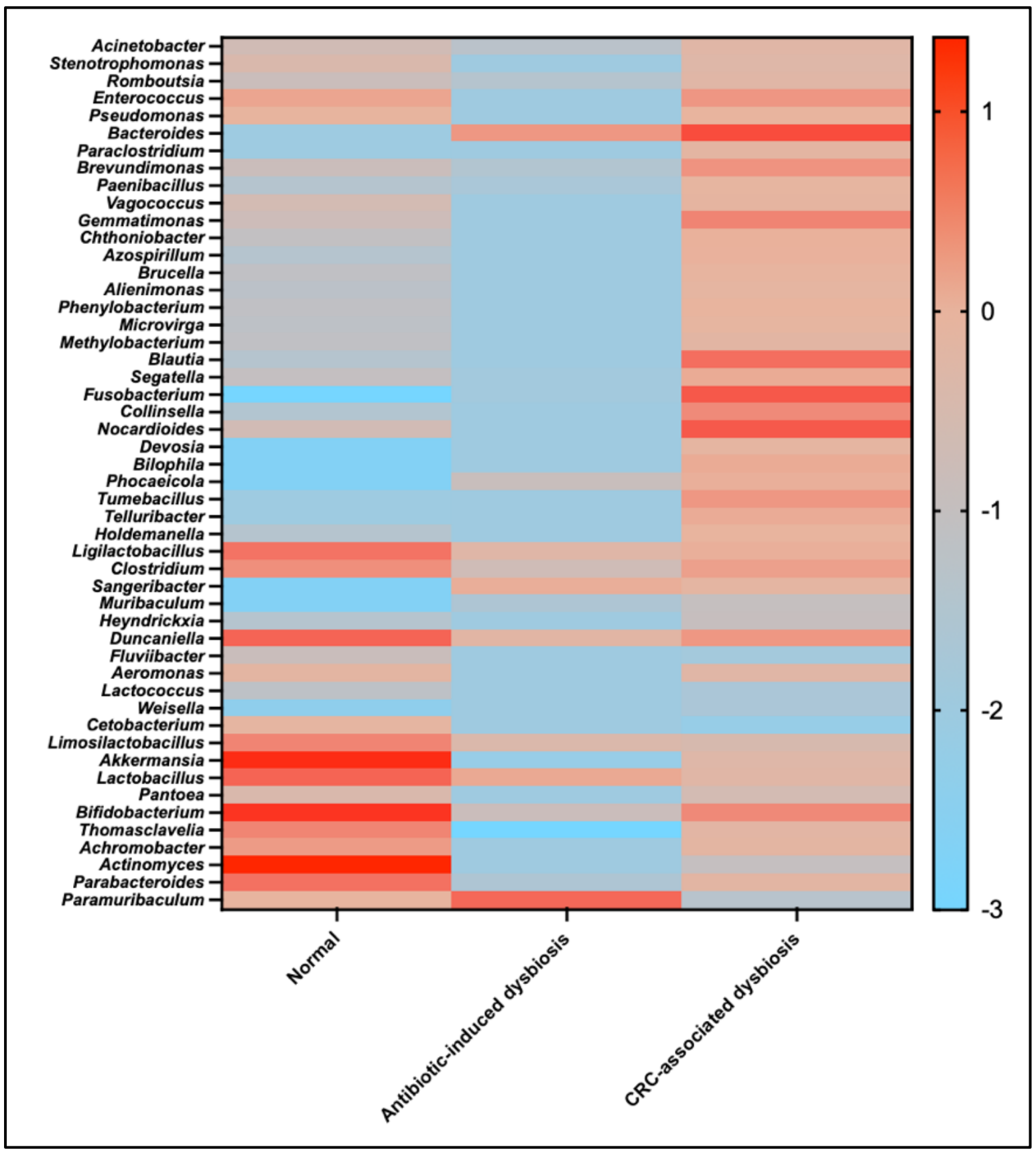

Figure S4. *Genus-level microbial composition heatmap. Heatmap showing z-score normalized relative abundance of the top 50 bacterial genera across pooled microbiome profiles from normal, antibiotic-induced dysbiosis, and CRC-associated dysbiosis groups. Colors indicate relative enrichment (red) or depletion (blue) of taxa within each microbiome state.*

2. **Secondary metabolites, host-, and tumor-derived cues differentially modulate CRC-associated bacterial growth**

Phenotypic assays were used as supportive characterization of the isolate. The observed antibiotic tolerance, hemolytic activity, and partial IMViC profile are consistent with reported biochemical characteristics of CRC-gut-associated ***Fusobacterium*** species and were not used as standalone taxonomic identifiers (Table S2). The representative images of *Fusobacterium*

isolation are given in Supplementary Fig. S5. Morphological characterization was done by Gram staining and FE-SEM, and representative images are given in Supplementary Fig. S6.

Biochemical Assay	Selection / Principle	Result	Interpretation	Differential Exclusion Rationale
**Vancomycin resistance**	Selective pressure against Gram-positive bacteria	**Growth observed**	Consistent with *Fusobacterium* spp.	Excludes Gram-positive genera such as *Clostridium*, *Enterococcus*, and *Lactobacillus*, supporting selection of Gram-negative anaerobes including *Fusobacterium*
**Neomycin resistance**	Selective pressure against facultative aerobes	**Growth observed**	Supports anaerobic gut-associated bacterium	Excludes facultative aerobic and enteric genera such as *Escherichia*, *Klebsiella*, and *Enterobacter*, enriching for obligate anaerobic gut-associated bacteria including *Fusobacterium*
**Hemolysis on blood agar**	Mild Erythrocyte lysis under anaerobic conditions	**Positive (β-hemolysis)**	Characteristic of *Fusobacterium*	Differentiates from non-hemolytic commensal genera such as *Bacteroides* and *Prevotella*, supporting identification of hemolytic anaerobes including *Fusobacterium* spp.
**Indole test**	Tryptophan	**Positive (+)**	Consistent with *Fusobacterium* spp.	Rules out indole-negative anaerobes such as *Bacteroides*

	degradation to indole			*fragilis* group members, while remaining consistent with indole-positive *Fusobacterium* spp.
**Methyl Red (MR)**	Stable mixed-acid fermentation	**Positive (+)**	Indicates acidogenic anaerobic metabolism	Supports fermentative anaerobic metabolism and excludes genera with mixed or neutral fermentation profiles such as *Enterobacter* and *Klebsiella*.
**Citrate utilization**	Use of citrate as sole carbon source	**Negative (–)**	Typical of gut-associated *Fusobacterium*	Excludes citrate-utilizing genera such as *Enterobacter*, *Klebsiella*, and *Citrobacter*, consistent with gut-associated *Fusobacterium* spp.

Supplementary Table S2. Biochemical profiling of isolated Fusobacterium sp.

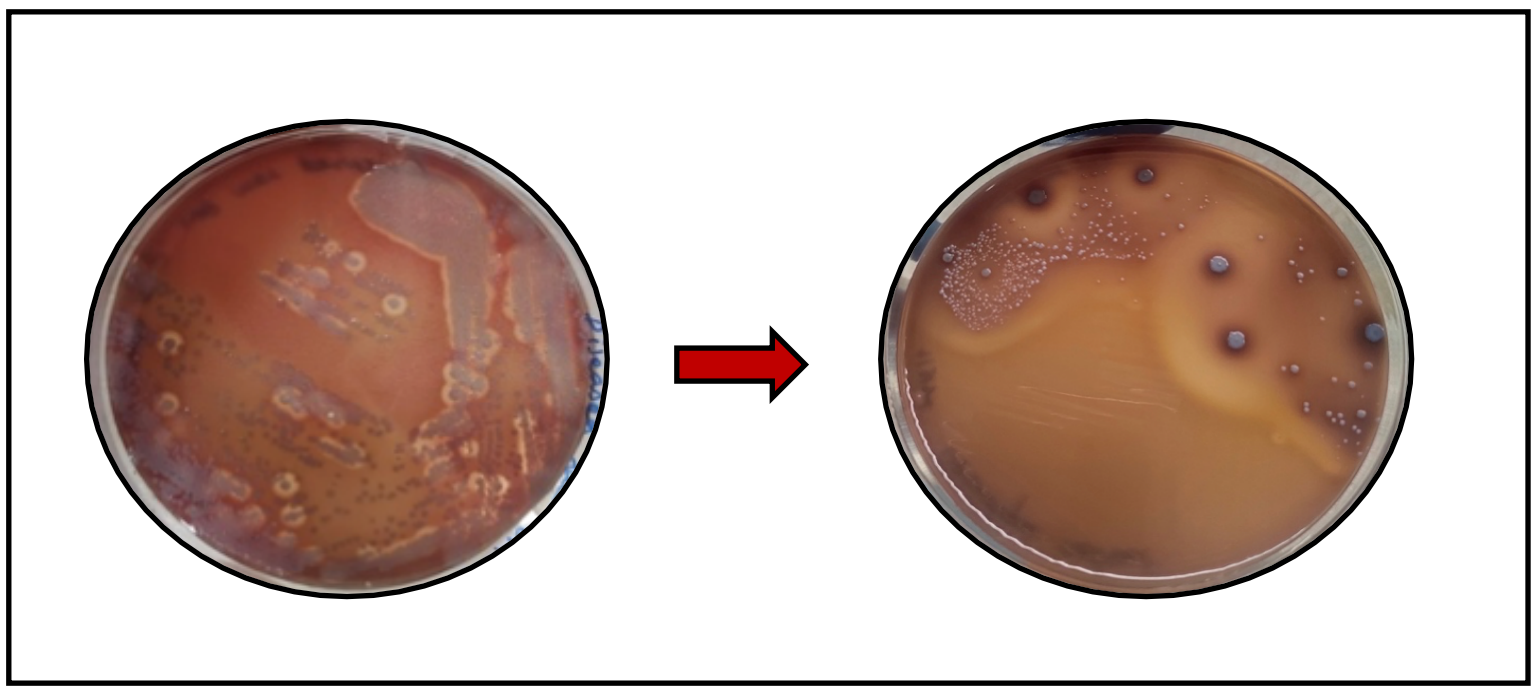

Figure S5. Isolation of Fusobacterium sp. using selective media.

Detailed bacterial growth kinetics underlying the integrated AUC analyses shown in Figure 2 are presented in Supplementary Fig S7. Growth curves (OD_{600} over time) demonstrate the temporal effects of MS, host epithelial conditioned media, and tumor-derived conditioned media on CRC-associated bacterial isolates and *Fusobacterium* sp.

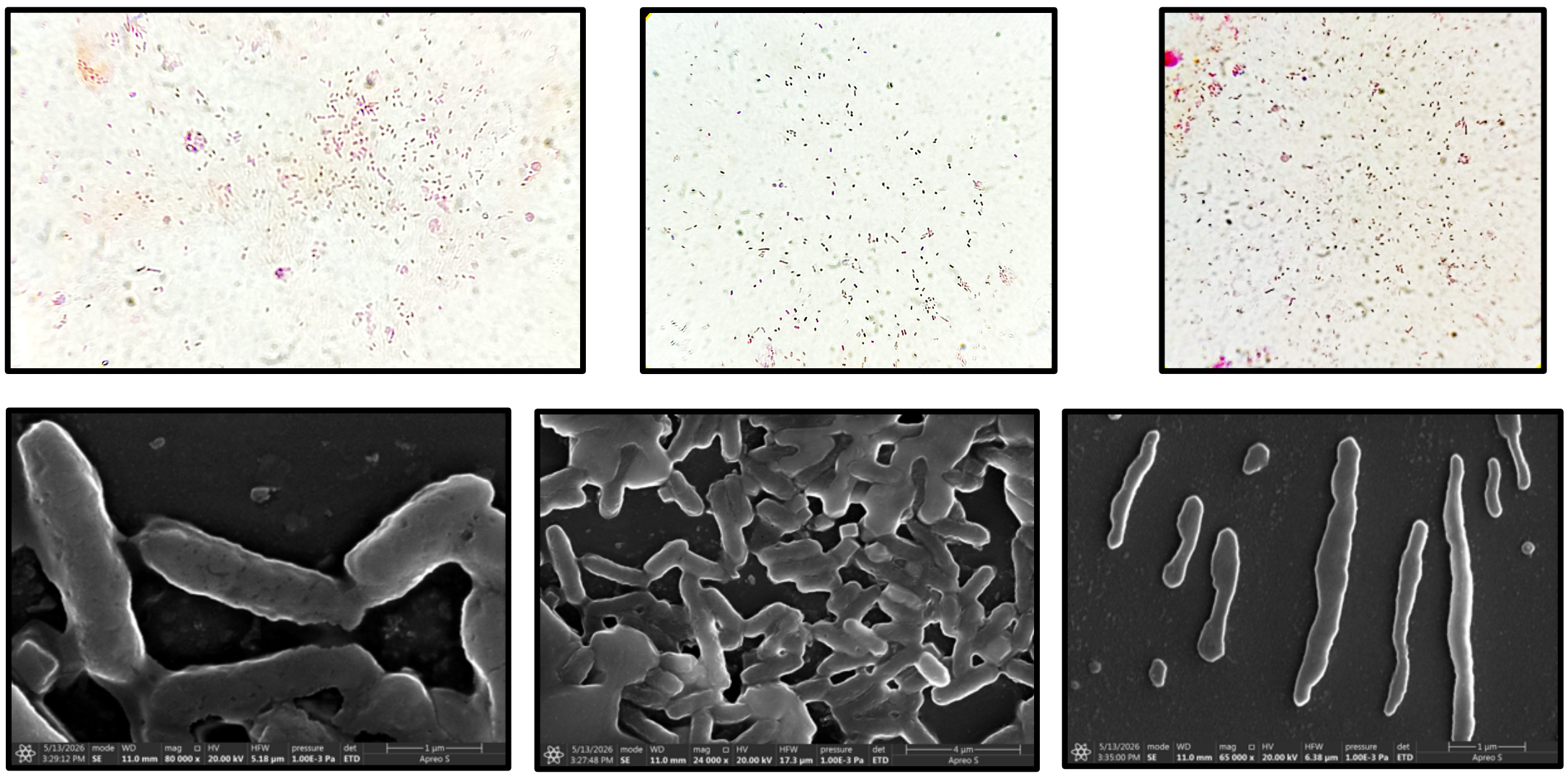

Figure S6. Morphological characterization of putative Fusobacterium sp. isolated from CRC-associated dysbiosis. *(Upper panel) Gram staining of the anaerobic bacterial isolate showing Gram-negative rod-shaped morphology consistent with Fusobacterium sp.; (Lower panel) Representative FE-SEM images of the isolate showing rod-shaped bacterial morphology under anaerobic culture conditions at different magnifications.*

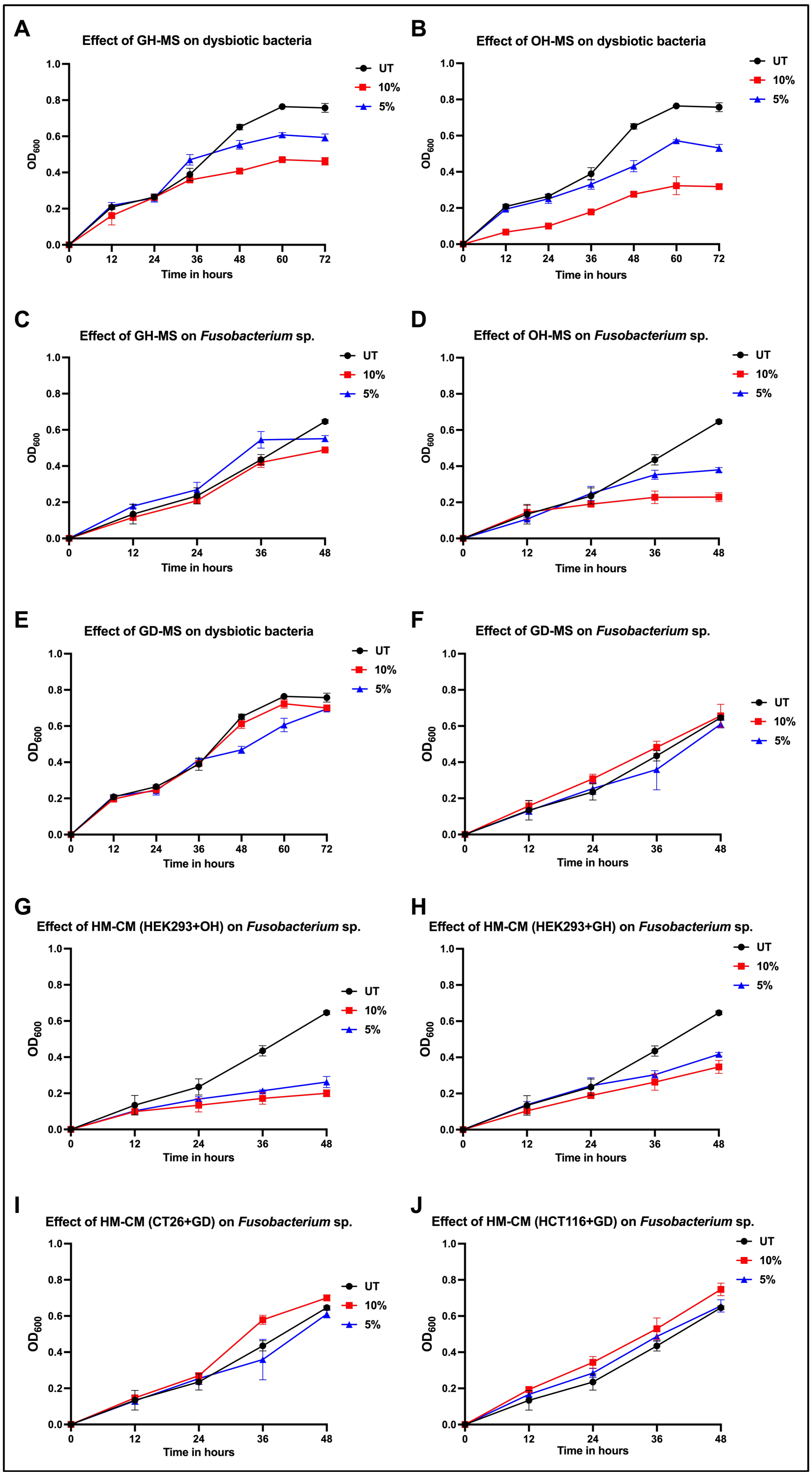

Figure S7. ***Growth kinetics of CRC-associated bacteria and Fusobacterium sp. under metabolite and conditioned media treatments.*** *Time-course growth curves (OD_{600}) for CRC-associated bacterial isolates and Fusobacterium sp. cultured under metabolite-enriched supernatant fractions (MSs), host-derived conditioned media (HM-CM), and tumor-derived conditioned media. Panels (A-B) show the effects of gut healthy (GH-MS) and oral healthy (OH-MS) metabolite fractions on CRC-associated dysbiotic bacteria. Panels (C-D) show the corresponding effects on Fusobacterium sp. Panels (E-F) show the effects of gut dysbiotic microbiota-derived metabolites (GD-MS) on CRC-associated bacteria* and *Fusobacterium sp., respectively. Panels (G-H) illustrate the effects of host epithelial cell-conditioned media derived from HEK293 cells combined with OH or GH metabolite fractions on Fusobacterium growth. Panels (I-J) show the effects of tumor cell-conditioned media derived from CT26 and HCT116 colorectal cancer cells combined with GD metabolite fractions. Untreated controls (UT) are shown for comparison. Data represent mean ± SEM from three independent experiments. Growth curves were used to calculate the integrated growth (area under the curve, AUC) presented in Figure 2.*

3. GH-MS and OH-MS reversibly suppress *Fusobacterium* sp. growth and impair host interaction

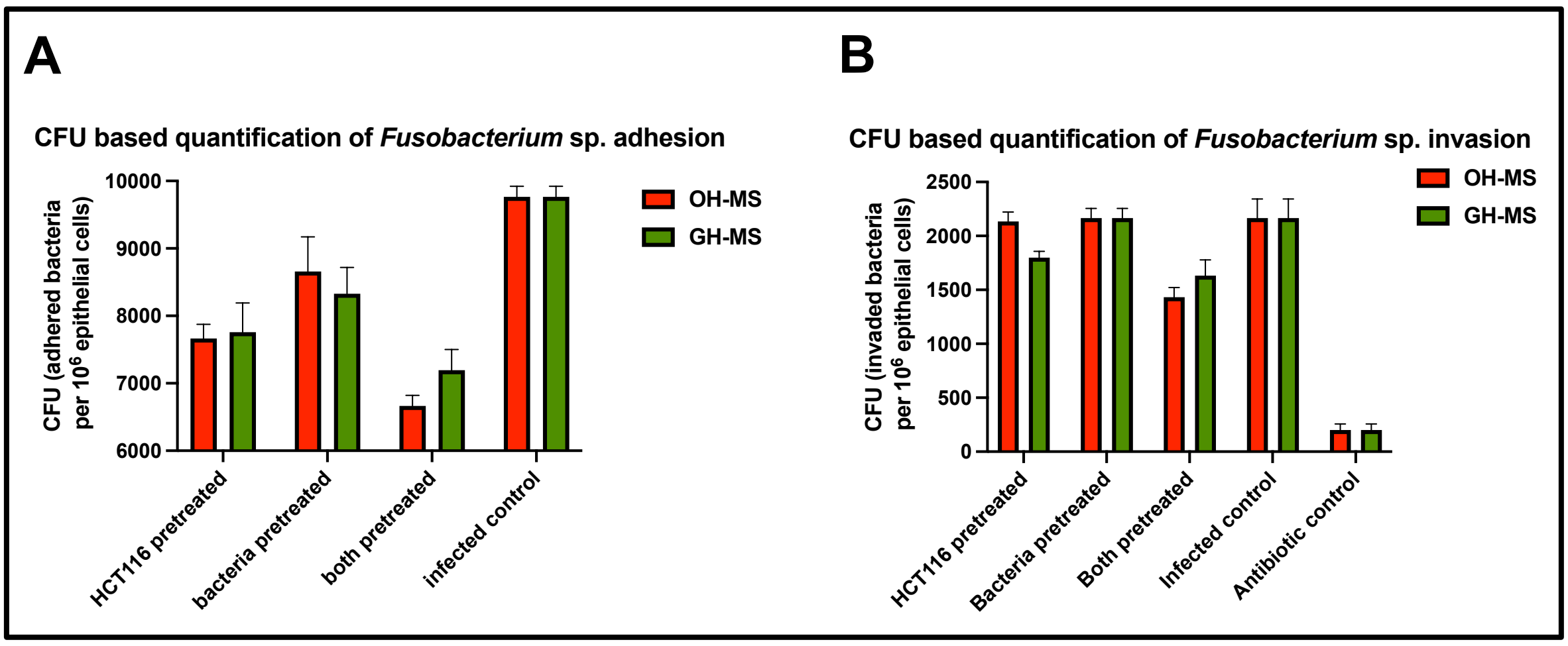

Figure S8. Absolute CFU-based quantification for adhesion and invasion of Fusobacterium sp. on HCT116 cells.

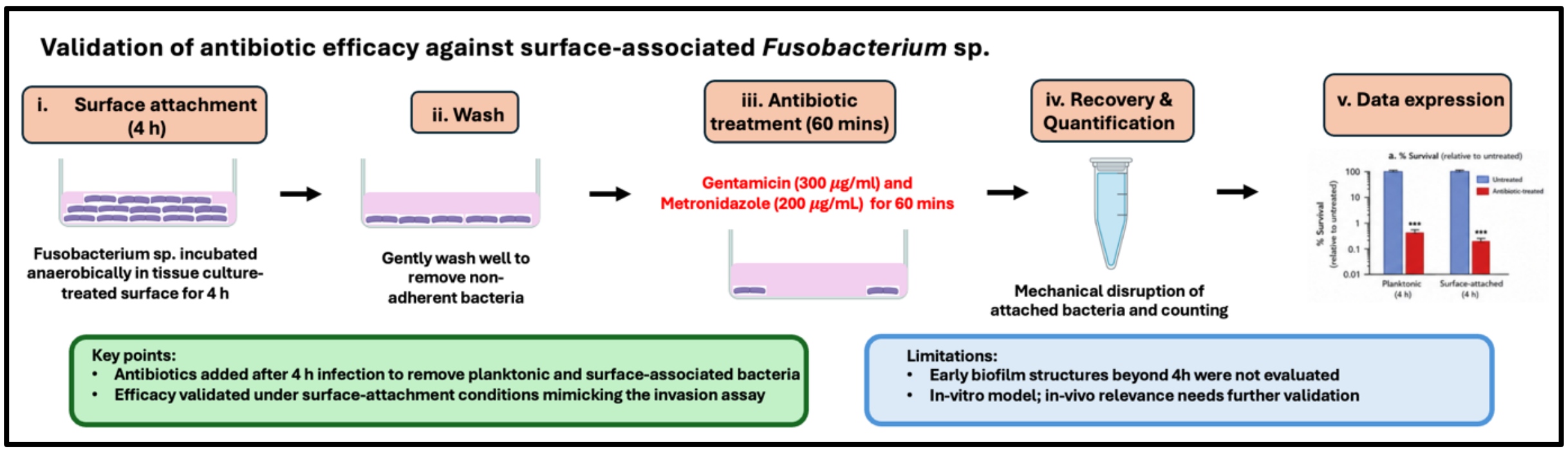

Figure S9. Schematic workflow of antibody validation against surface-associated Fusobacterium sp.

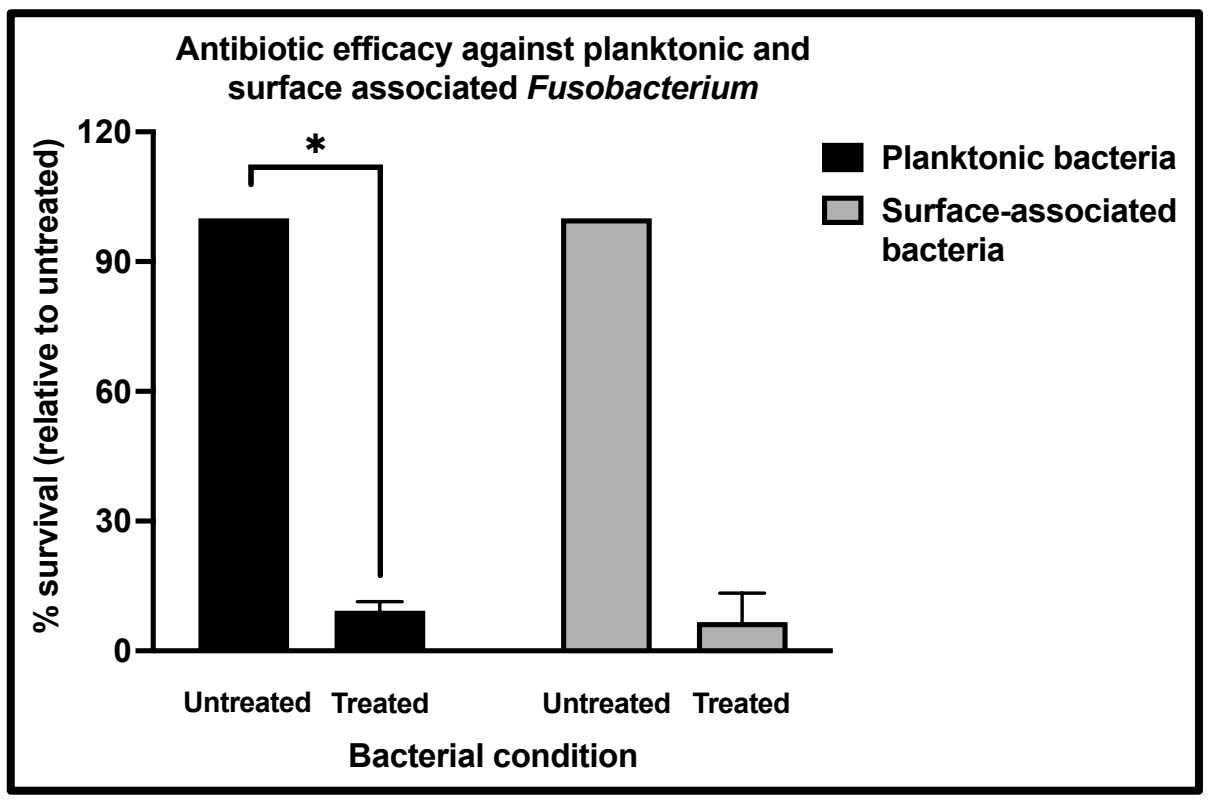

Figure S10.Antibiotic efficacy against planktonic and surface-associated Fusobacterium sp. *Fusobacterium cultures were analyzed under planktonic conditions or after 4 h surface attachment to tissue culture-treated wells to model early adhesion. Following incubation, extracellular bacteria were treated with gentamicin (300 µg/mL) and metronidazole (200 µg/mL) for 1 h. Viable bacteria were quantified by CFU enumeration. Survival was normalized to the respective untreated controls (set to 100%). Antibiotic treatment significantly reduced bacterial survival under planktonic conditions ($p < 0.05$) and produced a comparable reduction under surface-associated conditions, indicating effective elimination of extracellular bacteria under conditions mimicking the invasion assay. Data are presented as mean ± SEM (n = 3).*

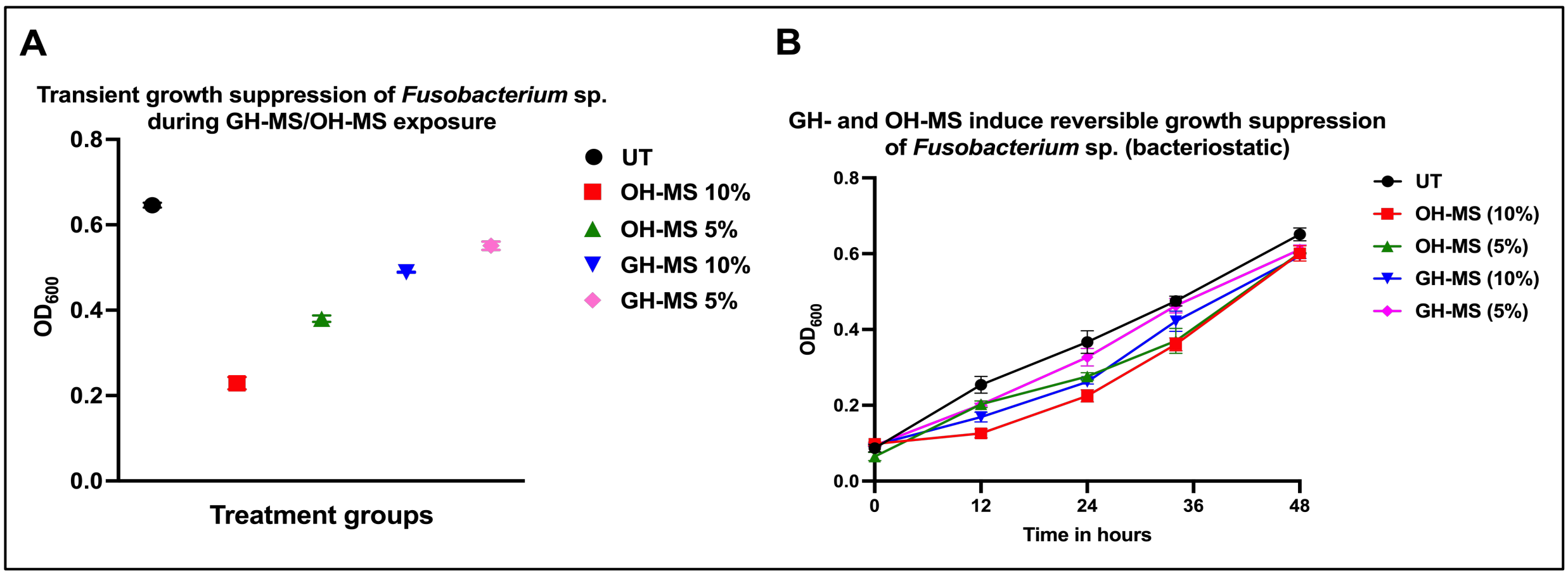

Figure S11. Reversible suppression of Fusobacterium growth by metabolite-enriched supernatant fractions*. (A) Optical density (OD_{600}) measurements of Fusobacterium sp. following 48 h exposure to metabolite-enriched supernatant fractions derived from oral healthy microbiota (OH-MS) and gut healthy microbiota (GH-MS) at 5% and 10% concentrations, compared with untreated controls (UT). Both metabolite fractions suppressed bacterial growth during exposure. (B) Growth recovery of Fusobacterium sp. following removal of MS fractions. Bacteria were washed and resuspended in fresh medium after exposure to OH-MS or GH-MS, and regrowth was monitored over time. Restoration of bacterial growth following washout indicates that MS-mediated growth suppression is reversible and consistent with a bacteriostatic effect. Data represent mean ± SEM from three independent experiments.*

4. MS fractions derived from healthy gut and oral microbiota suppress epithelial viability and spheroid growth in 2D and 3D models

Supplementary Fig. S12 shows the concentration-dependent effects of microbiota-derived metabolite supernatants (MS) on the viability of CT26 and HCT116 cells at 24, 48, and 72 h. Both GH-MS and OH-MS progressively reduced cell viability with increasing concentrations, whereas GD-MS and untreated controls showed minimal inhibitory effects across time points. Consistent with the observed reduction in viability, densitometric analysis of Bax/Bcl2 expression in CT26 cells demonstrated a significant increase in the Bax/Bcl2 ratio following GH-MS and OH-MS treatment, suggesting induction of apoptotic signaling.

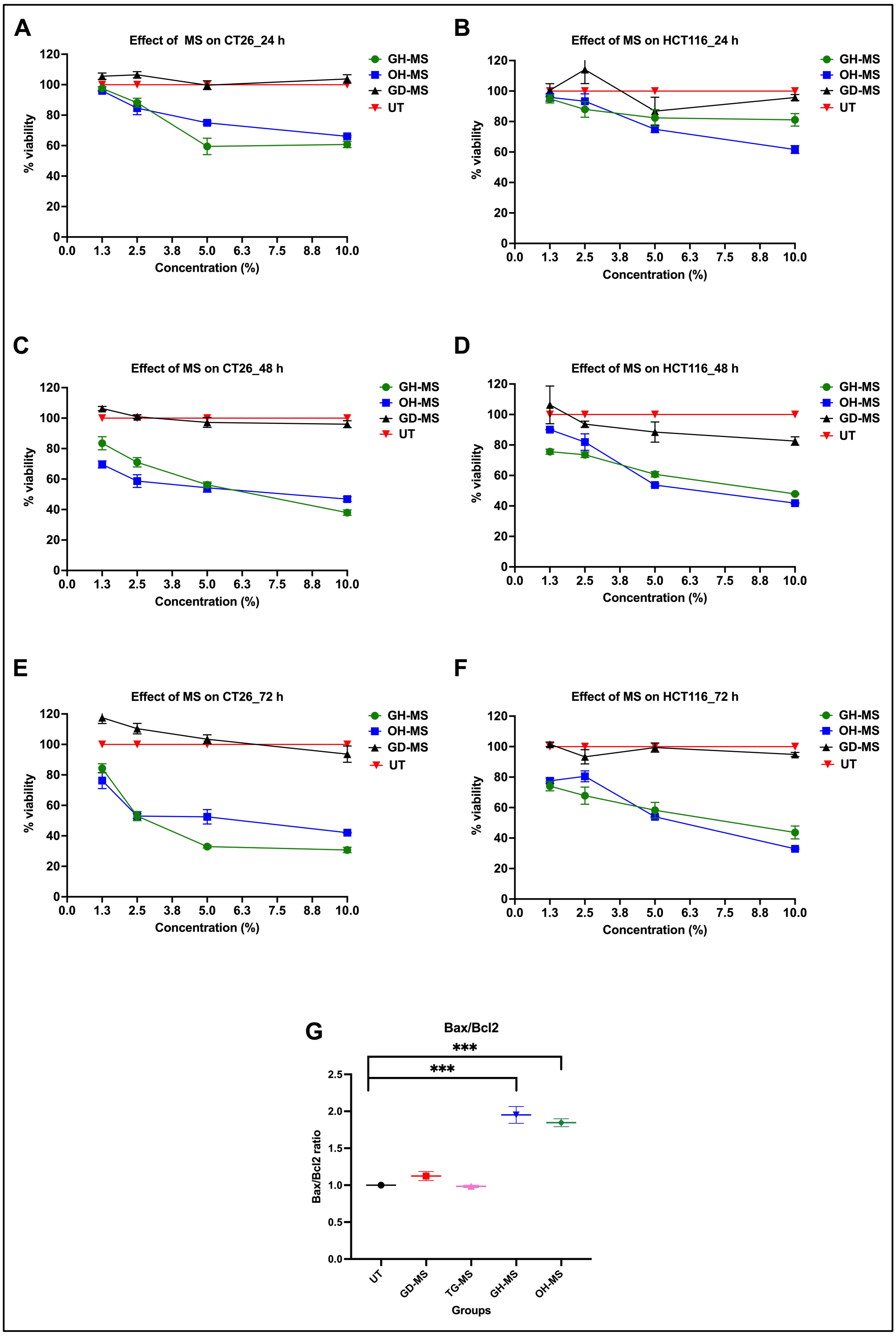

Figure S12. Concentration- and time-dependent effects of microbiota-derived metabolite supernatants on colorectal cancer cell viability and apoptosis markers. *(A-F) Dose-response analysis of metabolite supernatants (MS) on CT26 and HCT116 cell viability measured by MTT assay at 24 h (A, B), 48 h (C, D), and 72 h (E, F)***.*** *Cell viability is expressed as percentage relative to untreated controls. Data represent mean ± SEM from three independent experiments. (G) Densitometric analysis of Bax/Bcl2 protein expression ratio in CT26 cells following treatment with different MS conditions. Increased Bax/Bcl2 ratios were observed in GH-MS and OH-MS treated cells compared with control groups, indicating activation of apoptotic signaling pathways. Statistical significance was determined by one-way ANOVA (*p < 0.05, ***p < 0.001).*

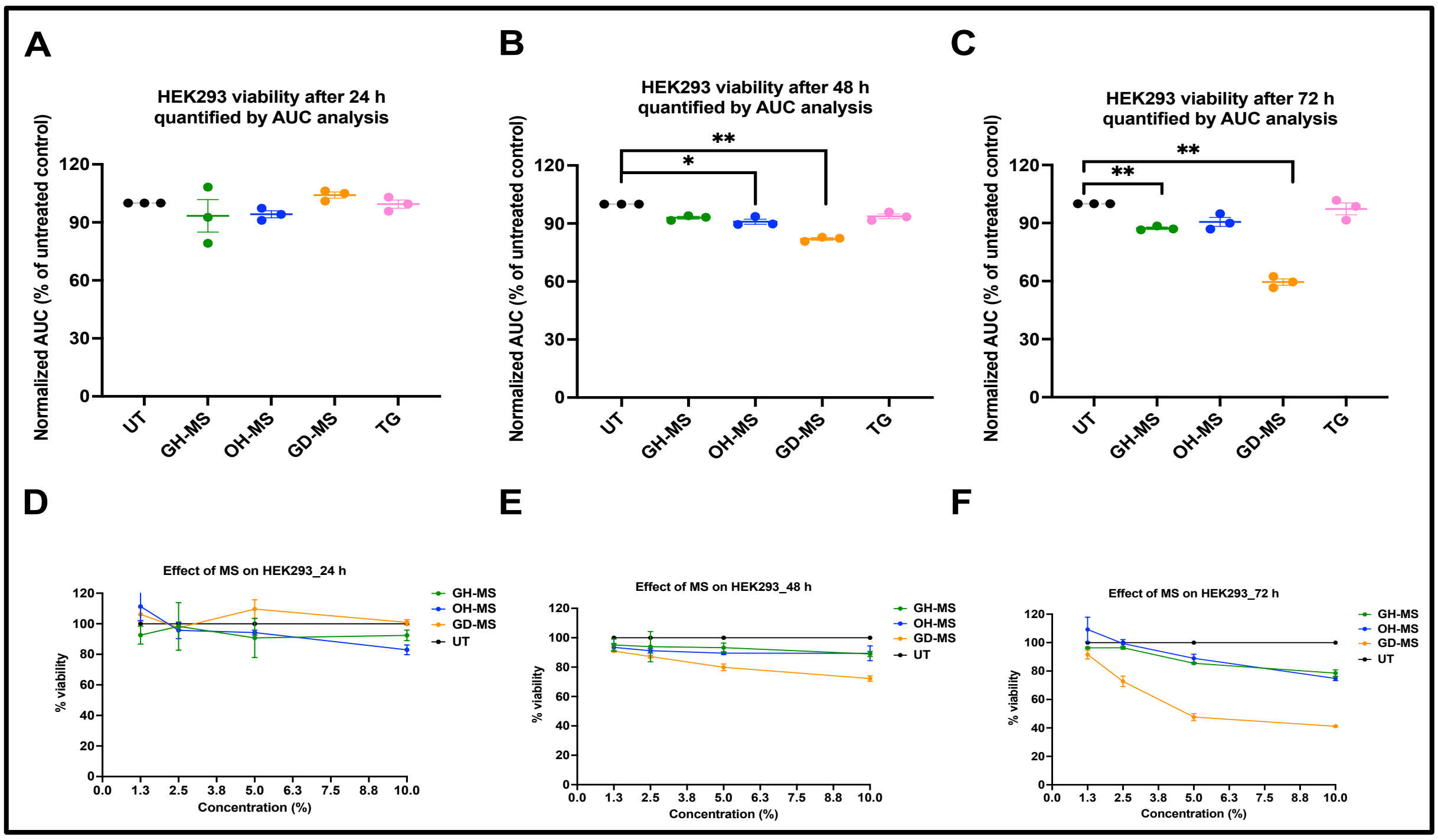

***Figure S13*. Effects of microbiota-derived metabolite-enriched supernatants (MS) on viability of non-cancerous HEK293 cells.** *(A-C) Area under the curve (AUC)-based quantification of HEK293 cell viability following treatment with GH-MS, OH-MS, and GD-MS at 24 h (A), 48 h (B), and 72 h (C). Viability is expressed as percentage relative to untreated control (UT); (D-F) Dose-dependent effects of MS on HEK293 viability across increasing concentrations (1-10%) at 24 h (D), 48 h (E), and 72 h (F); GH-MS: gut healthy microbiota-derived metabolite supernatant; OH-MS: oral microbiota-derived metabolite supernatant; GD-MS: dysbiotic microbiota-derived metabolite supernatant. Data represent mean ± SEM from three independent experiments. Statistical significance was determined using one-way ANOVA (*p < 0.05, **p < 0.01).*

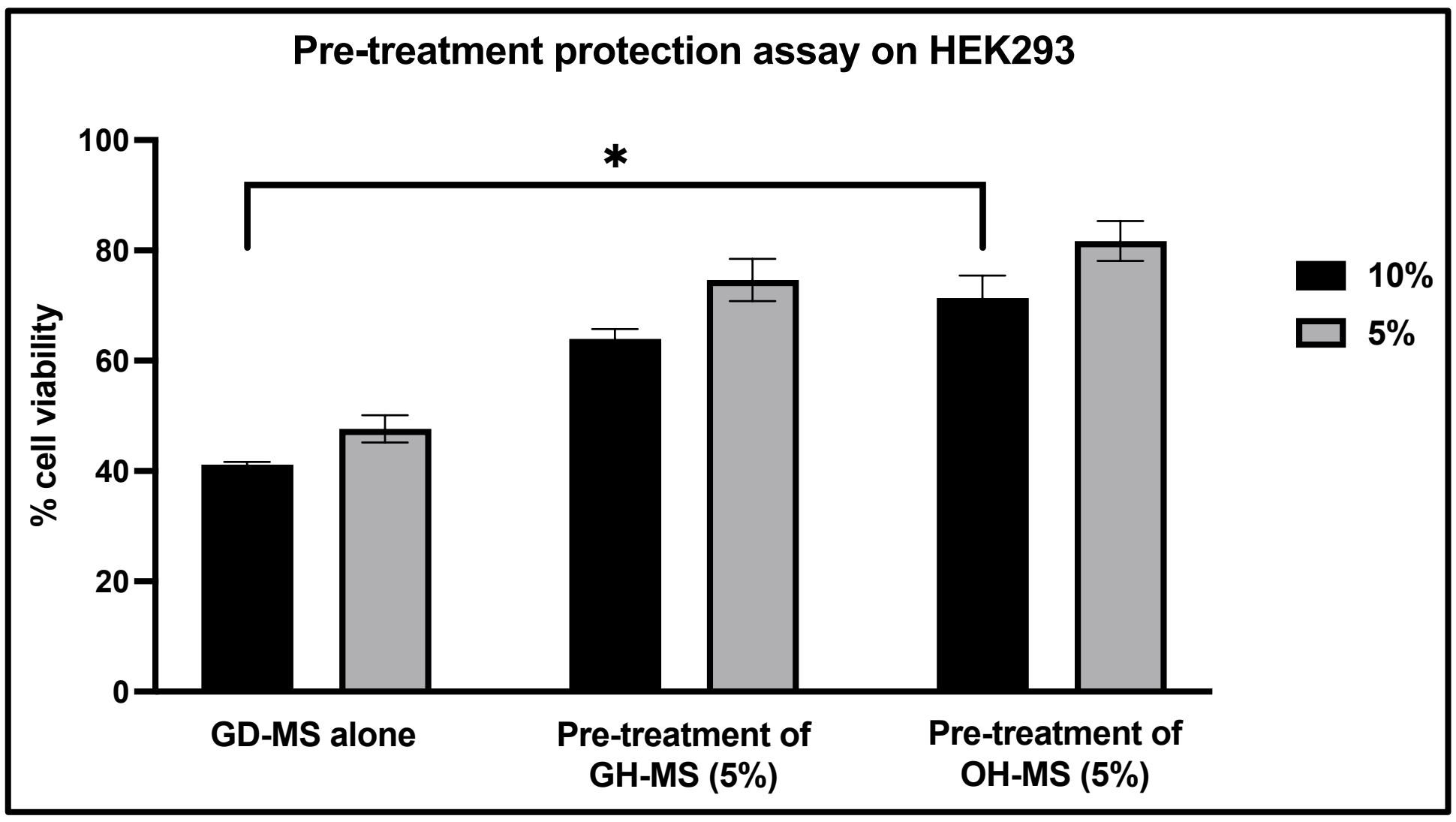

***Figure S14*. Protective effects of healthy microbiota-derived metabolite preconditioning against GD-MS-induced epithelial stress in HEK293 cells.** *HEK293 cells were pre-treated with GH-MS or OH-MS (5% for 4 hours) prior to GD-MS exposure, and viability was assessed by MTT assay. Preconditioning with healthy microbiota-derived metabolite fractions partially restored viability compared with GD-MS treatment alone. Data represent mean ± SEM from three independent experiments. Statistical significance was determined using one-way ANOVA followed by Dunnet's multiple comparison's test (*p<0.05).*

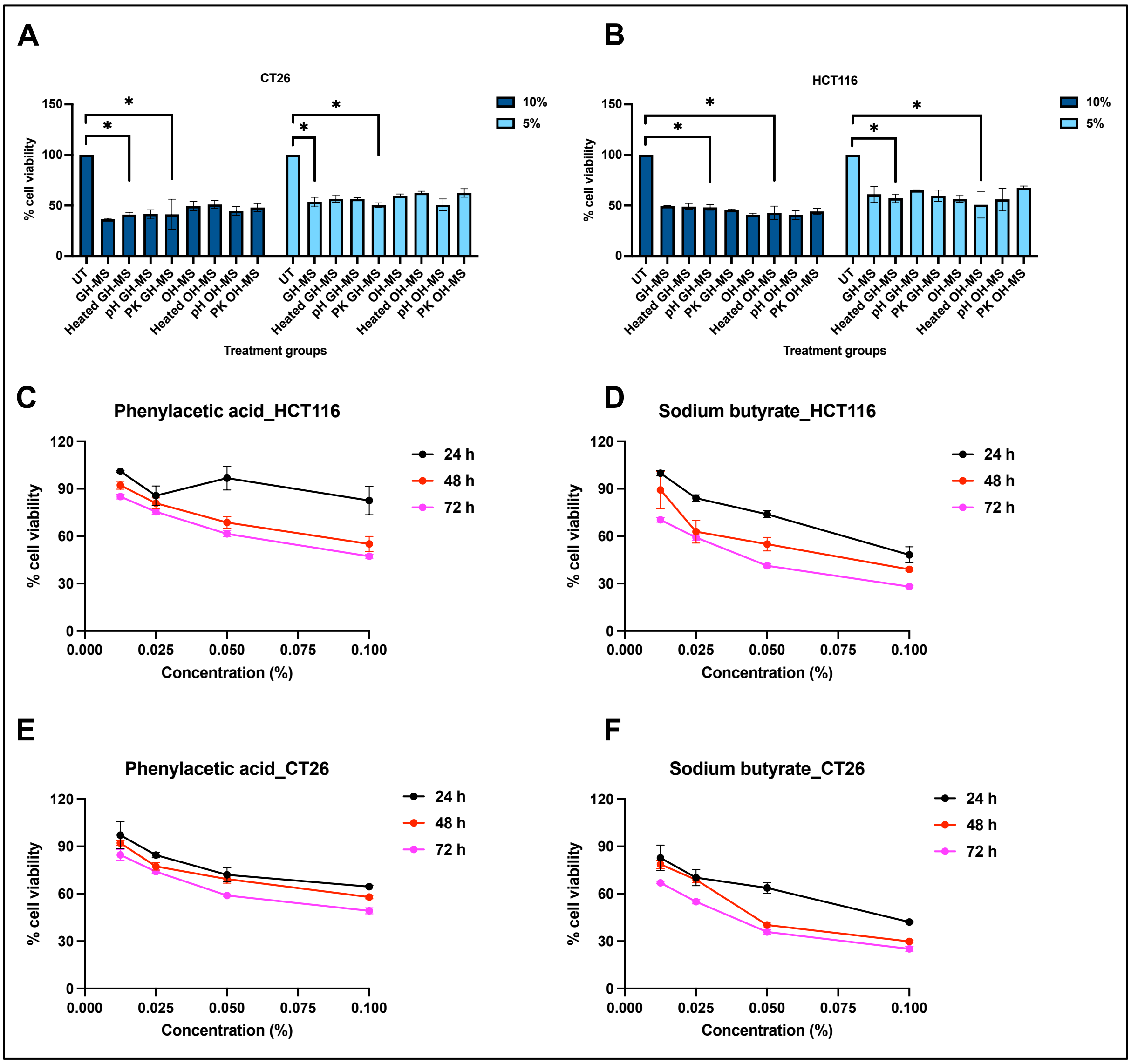

Supplementary Figure S15. Biochemical characterization of microbiota-derived metabolite-enriched supernatants (MS) and functional validation of representative metabolites.*(A-B) CT26 and HCT116 cells were treated with untreated GH-MS/OH-MS or corresponding heat-treated, proteinase K-treated, and pH-normalized preparations at indicated concentrations (5% and 10%) for 48 h followed by MTT assay. (C-F) Functional validation of representative metabolites identified by LC-MS profiling. CT26 and HCT116 cells were treated with increasing concentrations of sodium butyrate or phenylacetic acid for 24, 48, and 72 h followed by MTT assay. Data are presented as mean ± SEM from three independent biological replicates. Statistical significance was determined using two-way ANOVA followed by Dunnett's multiple comparison test. *p < 0.05 compared with untreated control.*

In-silico docking of metabolites derived from healthy microbiota with Bcl-2

Phenylacetic acid binds to BCL-2 (PDB ID: 6O0K) within a predominantly hydrophobic groove, consistent with the canonical BH3-binding pocket, where its aromatic and acidic features are differentially recognized; the phenyl ring is deeply accommodated by a hydrophobic cluster formed by PHE 104, TRP 144, PHE 198, TYR 202, VAL 148, and GLY 145, enabling favorable π–π stacking and van der Waals interactions that anchor the ligand core, while the acetic acid moiety is oriented toward the pocket entrance and forms a key hydrogen-bond interaction between the carboxylic acid group and ASP 103, providing

directional stabilization; additional electrostatic and polar contributions arise from proximity to ARG 107, which further supports binding of the acidic head group, and ALA 100 helps shape the pocket without direct strong contacts (Fig. S16A); overall, the interaction profile reflects a binding mode dominated by hydrophobic enclosure of the phenyl ring with a single polar anchor, characteristic of weak to moderate affinity interactions of small aromatic acids within the BCL-2 BH3 groove and suggesting potential for affinity enhancement through hydrophobic ring substitution or extension toward additional pocket subregions.

Succinic acid binds within the BCL-2 (6O0K) pocket mainly through polar interactions, with a key hydrogen bond formed between one of its carboxylate oxygens and Asp103, which appears to be the primary stabilizing contact. The ligand sits in a pocket lined by a mix of hydrophobic and polar residues, including Phe104, Phe198, Val148, and Trp144 that provide van der Waals support to the carbon backbone, while Arg107 and Tyr108/Tyr202 are positioned to contribute additional electrostatic or hydrogen-bonding interactions, possibly water-mediated (Fig. S16B). Because succinic acid is small and highly polar compared with typical BCL-2 inhibitors, its binding is likely weaker and driven more by this limited hydrogen-bond network and pocket shape complementarity than by extensive hydrophobic burial, suggesting a modest and potentially transient interaction rather than tight inhibitory binding.

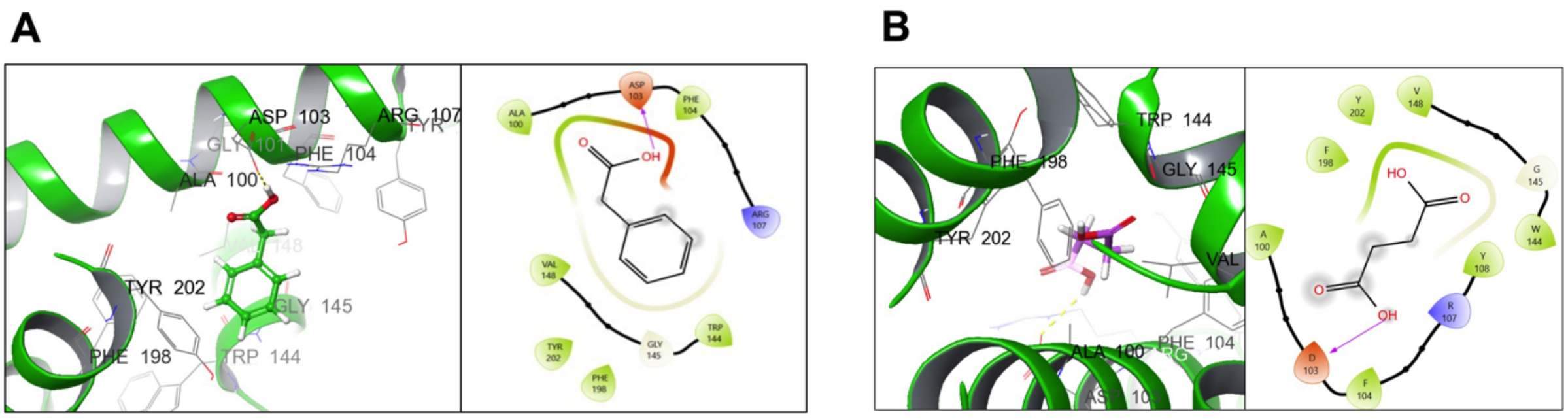

Figure S16. In silico docking of Phenylacetic acid (A) and Succinic acid (B) with Bcl-2.

In-silico docking of metabolites derived from healthy microbiota with HDAC-2

Phenylacetic acid binds in the HDAC2 active site (PDB: 5IWG) with its carboxylate group coordinating the catalytic **Zn^{2+} ion (Zn401)**, which is the central anchoring interaction typical of HDAC inhibitors. One oxygen of the carboxylate appears to form a metal coordination bond with zinc, while the other participates in hydrogen bonding with nearby residues such as **Tyr308** and possibly **His145**, helping stabilize the ligand's polar head within the catalytic pocket. Additional hydrogen-bond or electrostatic support may come from **His146** and **Asp181**, which are positioned along the rim of the active site channel (Fig. S17A). The phenyl

ring extends into the hydrophobic tunnel of HDAC2, where it is surrounded by residues like **Leu144, Phe155, Cys156, and Met35**, forming van der Waals and aromatic interactions that help position the ligand within the narrow channel. Glycine residues (Gly142, Gly143, Gly154, Gly305, Gly306) contribute to shaping the flexible backbone of the pocket rather than making strong side-chain contacts. Overall, binding is driven by classic **zinc chelation at the catalytic center combined with hydrophobic packing of the aromatic ring**, consistent with the recognition mode of small carboxylic acid HDAC ligands, though likely weaker than bulkier hydroxamate-based inhibitors.

Lactate binds in the HDAC2 catalytic pocket (5IWG) by anchoring its carboxylate group to the catalytic **Zn^{2+} ion (Zn401)**, which is the dominant interaction stabilizing the ligand. One oxygen of the lactate carboxylate coordinates directly with zinc, while the other forms a hydrogen bond with **Tyr308**, a key residue that often participates in stabilizing zinc-bound inhibitors. The hydroxyl group on lactate is oriented toward **His145**, suggesting an additional hydrogen bond that helps position the molecule within the active site channel. Surrounding residues such as **His146, Asp181, and Asp269** contribute to the polar environment of the catalytic pocket and may provide secondary electrostatic stabilization or water-mediated contacts (Fig. S17B). The small aliphatic portion of lactate sits in a relatively narrow hydrophobic corridor lined by residues including **Leu144, Met35, Phe155, and Cys156**, forming modest van der Waals contacts but not deeply filling the channel. Several glycine residues (Gly143, Gly154, Gly305, Gly306) shape the backbone of the pocket without strong side-chain interactions. Overall, lactate shows a **simple, low-affinity binding mode** dominated by zinc coordination and a small hydrogen-bond network, lacking the extended hydrophobic and bidentate metal-chelating features seen in more potent HDAC inhibitors.

Threonine occupies the HDAC2 catalytic pocket (PDB: 5IWG) with its **carboxylate group coordinating the catalytic Zn^{2+} ion (Zn401)**, forming the principal anchoring interaction in a manner typical for small carboxylate-containing ligands. One oxygen of the carboxylate engages in metal coordination while also forming a hydrogen bond with **Tyr308**, which helps stabilize the zinc-bound state. The α-amino group of threonine is oriented toward the lower part of the pocket near **Gly305/Gly306** and may participate in hydrogen bonding with backbone carbonyls or structured water molecules rather than strong direct side-chain contacts. The side-chain hydroxyl group projects toward **His145/His146**, suggesting a possible hydrogen bond that helps define ligand orientation within the channel (Fig. S17C). The surrounding environment includes polar catalytic residues such as **Asp181 and His183**, which help maintain the electrostatic environment of the zinc center, while hydrophobic tunnel

residues like **Leu144, Phe155, Cys156, and Met35** provide van der Waals contacts along the ligand's carbon framework. Overall, threonine exhibits a **simple, low-affinity binding mode dominated by monodentate zinc coordination and a small hydrogen-bond network**, lacking the extended hydrophobic cap and strong bidentate zinc-chelating group characteristic of potent HDAC inhibitors.

Glutamic acid binds in the HDAC2 catalytic pocket (PDB: 5IWG) through a network of polar and metal-coordination interactions centered on the catalytic **Zn^{2+} ion (Zn401)**. One of the carboxylate oxygens coordinates directly with zinc, serving as the principal anchoring point, while the second oxygen forms a hydrogen bond with **Tyr308**, a key residue that stabilizes zinc-bound ligands in HDAC enzymes. The side-chain carboxylate of glutamate extends deeper into the pocket and is positioned to form additional hydrogen bonds with **His145 and His146**, helping orient the ligand within the active-site channel. The α-amino group lies closer to the entrance of the pocket near **Gly154** and may engage in backbone-mediated hydrogen bonding or water-bridged contacts rather than strong side-chain interactions. The surrounding environment includes **Gly305 and Gly306**, which shape the lower pocket contour, and hydrophobic residues such as **Phe155, Cys156, Leu144, and Met35**, which provide van der Waals stabilization along the ligand's carbon backbone (Fig. S17D). Overall, glutamic acid shows a **polar, zinc-anchored binding mode dominated by monodentate metal coordination and multiple hydrogen bonds**, but, like other small endogenous acids, it lacks the extended hydrophobic cap and strong bidentate zinc-chelating group characteristic of high-affinity HDAC inhibitors, indicating relatively weak binding.

Butyric acid binds within the HDAC2 catalytic pocket (PDB: 5IWG) in a manner typical of small carboxylate HDAC ligands, with its **carboxylate group coordinating the catalytic Zn^{2+} ion (Zn401)** as the primary anchoring interaction. One oxygen of the carboxylate engages in metal coordination while the other forms a hydrogen bond with **Tyr308**, helping stabilize the zinc-bound state. The hydroxyl proton of the carboxylic acid is oriented toward **His145**, suggesting an additional hydrogen bond that contributes to positioning the ligand in the active site. The aliphatic chain of butyrate extends upward into the narrow hydrophobic channel, where it makes van der Waals contacts with residues such as **Leu144, Phe155, Cys156, and Met35**, providing modest hydrophobic stabilization but not deeply occupying the tunnel. Polar catalytic residues including **His146, Asp181, and His183** shape the electrostatic environment around the zinc center and may contribute indirect, water-mediated interactions (Fig. S17E). Structural glycines (**Gly154, Gly305, Gly306**) help define pocket geometry without strong side-chain contacts. Overall, butyric acid exhibits a **simple, low-affinity binding mode**

dominated by monodentate zinc coordination and limited hydrophobic interactions, lacking the extended cap group and strong bidentate zinc-chelating functionality seen in more potent HDAC inhibitors.

Citramalic acid binds in the HDAC2 catalytic pocket (PDB: 5IWG) through a multidentate polar interaction network centered on the catalytic **Zn^{2+} ion (Zn401)**. One of its carboxylate groups coordinates directly with zinc, forming the primary anchoring interaction, while the second carboxylate engages in hydrogen bonding with **Tyr308**, a residue commonly involved in stabilizing zinc-bound ligands in HDAC enzymes. The additional hydroxyl substituent on citramalate is oriented toward the upper region of the pocket and appears to form a hydrogen bond with a **bound water molecule**, which is in turn positioned near **His183**, suggesting a water-mediated interaction that further stabilizes the ligand. The ligand's chiral center helps orient the backbone so that its polar groups remain clustered near the metal center, while the small alkyl portion extends into a shallow hydrophobic region bordered by **Phe155, Gly154, and Phe210**, contributing modest van der Waals contacts (Fig. S17F). Residues such as **His145, His146, Asp181, and Asp269** define the polar catalytic environment and may provide additional electrostatic or indirect stabilization. Overall, citramalic acid displays a **zinc-anchored, hydrogen-bond–rich binding mode** with possible water mediation, but due to its small size and limited hydrophobic surface, it likely binds weakly compared with larger HDAC inhibitors that possess extended hydrophobic caps and stronger bidentate zinc-chelating groups.

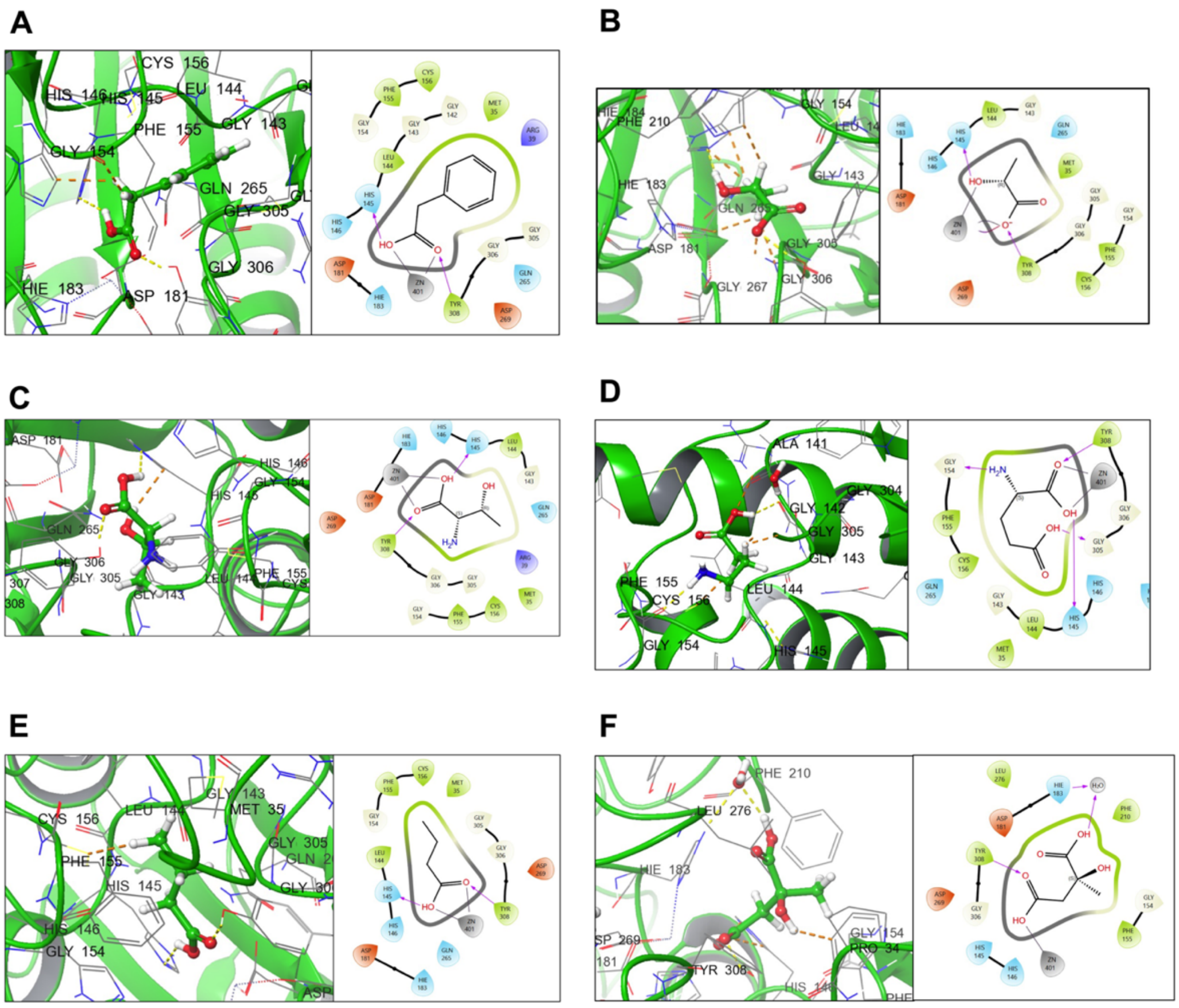

Figure S17. In-silico docking of phenylacetic acid (A), lactate (B), threonine (C), glutamic acid (D), butyric acid (E) and citramalic acid(F) with HDAC-2.

In-silico docking of metabolites derived from healthy microbiota with HDAC-8

Phenylacetic acid binds in the HDAC8 catalytic pocket (PDB: 1T69) with its carboxylate group coordinating near the catalytic Zn^{2+} ion (Zn378), a hallmark interaction for HDAC inhibitors. One oxygen of the carboxyl group is positioned to interact with the zinc center, while the hydroxyl oxygen can form hydrogen bonds with nearby histidines (His142 and His143) that participate in the metal-coordination network. The phenyl ring extends into the hydrophobic channel, making van der Waals contacts with Phe152, Phe208, Cys153, and surrounding aliphatic residues, helping anchor the ligand in the pocket (Fig. S18A). Additional stabilization may come from interactions with Tyr306 and backbone atoms near Gly304 at the rim of the channel. Overall, binding is zinc-assisted and hydrophobically supported, but the ligand is relatively small and lacks a long linker or capping group, so it likely shows weak HDAC8 inhibition compared with classical hydroxamate-based inhibitors.

Lactate occupies the HDAC8 catalytic pocket (PDB: 1T69) in a metal-centered binding mode. Its carboxylate group coordinates with the catalytic Zn^{2+} ion (Zn378), forming a key

electrostatic/metal interaction typical of small carboxylic acids in HDAC active sites. The same carboxylate oxygen also participates in hydrogen bonding with Tyr306, helping position the ligand in the catalytic channel. The hydroxyl group of lactate forms additional hydrogen bonding with His143 (and possibly His142), which are part of the zinc-binding and proton-relay network. Surrounding hydrophobic residues such as Phe152, Phe208, Trp141, and Cys153 line the pocket and provide weak van der Waals stabilization, though lactate's small size limits extensive hydrophobic contact (Fig. S18B). Overall, lactate shows a simple zinc-anchored, hydrogen bond–supported interaction, but due to its short chain and lack of a hydrophobic cap group, it would be expected to bind weakly compared with classical HDAC8 inhibitors.

Citramalic acid binds within the HDAC8 catalytic pocket (PDB: 1T69) primarily through its carboxylate group, which coordinates with the catalytic Zn^{2+} ion (Zn378), anchoring the ligand in a typical metal-dependent interaction. One carboxylate oxygen also forms a hydrogen bond with Tyr306, a key residue lining the active site channel. The hydroxyl substituent on the chiral center contributes additional stabilization via hydrogen bonding with His143 (and possibly His142), part of the catalytic proton-transfer network (Fig. S18C). Surrounding residues such as Phe208, Cys153, and Gly304 provide mild van der Waals contacts but limited hydrophobic enclosure. Overall, citramalic acid shows a zinc-chelating, hydrogen bond–driven binding mode, yet its small size and high polarity suggest weak inhibitory potential compared with classical HDAC8 inhibitors.

Threonine binds in the HDAC8 catalytic pocket (PDB: 1T69) mainly through polar and metal-associated interactions. Its carboxylate group coordinates with the catalytic Zn^{2+} ion (Zn378) and also forms a hydrogen bond with Tyr306, helping anchor the ligand near the metal center. The β-hydroxyl group of threonine participates in hydrogen bonding with His143 (and possibly His142), which are part of the catalytic histidine pair involved in proton transfer. Meanwhile, the amino group (NH_2) is oriented toward the pocket entrance and can form additional hydrogen bonding with nearby backbone residues such as Gly151 or Gln263 (Fig. S18D). Surrounding residues including Phe152, Phe208, Cys153, and Met274 provide weak van der Waals contacts, but the pocket remains largely polar around the ligand. Overall, threonine shows a zinc-associated, hydrogen bond–dominated binding mode, suggesting low affinity and weak inhibitory potential compared with typical HDAC8 inhibitors that possess stronger zinc-chelating groups and hydrophobic capping regions.

Butyric acid binds shallowly in the HDAC8 catalytic pocket (PDB: 1T69) with a simple, weak interaction pattern. The carboxylate group coordinates to the catalytic Zn^{2+} ion (Zn378) and

forms a hydrogen bond with Tyr306, which helps anchor the ligand near the metal center. A secondary polar contact is seen between the carboxyl oxygen and His143, part of the catalytic histidine pair. The aliphatic chain of butyrate extends into a narrow hydrophobic channel lined by Phe152, Phe208, Met274, and Cys153, where it makes weak van der Waals interactions (Fig. S18E). However, the molecule lacks a strong zinc-chelating motif and a bulky surface-interacting cap group, so its binding is dominated by single-point metal coordination plus limited hydrophobic contacts, indicating low affinity and weak inhibitory potential compared with classical HDAC8 inhibitors.

Glutamic acid binds in the HDAC8 catalytic pocket (PDB: 1T69) primarily through polar and metal-associated interactions. One carboxylate oxygen coordinates with the catalytic Zn^{2+} ion (Zn378), while the same group forms a hydrogen bond with Tyr306, helping position the ligand in the metal center region. The second carboxyl group extends toward the pocket entrance, forming additional hydrogen bonding with His143 from the catalytic histidine pair (Fig. S18F). The α-amino group (NH_2) contributes further polarity and may engage in weak electrostatic or hydrogen-bond interactions with nearby backbone atoms. The carbon chain sits along a hydrophobic channel lined by Phe152, Phe208, Cys153, and Met274, giving modest van der Waals stabilization. Overall, binding is driven by Zn^{2+} coordination plus multiple hydrogen bonds, but the small size and high polarity of glutamic acid limit deep hydrophobic engagement, suggesting weak inhibitory strength compared with classical HDAC8 inhibitors.

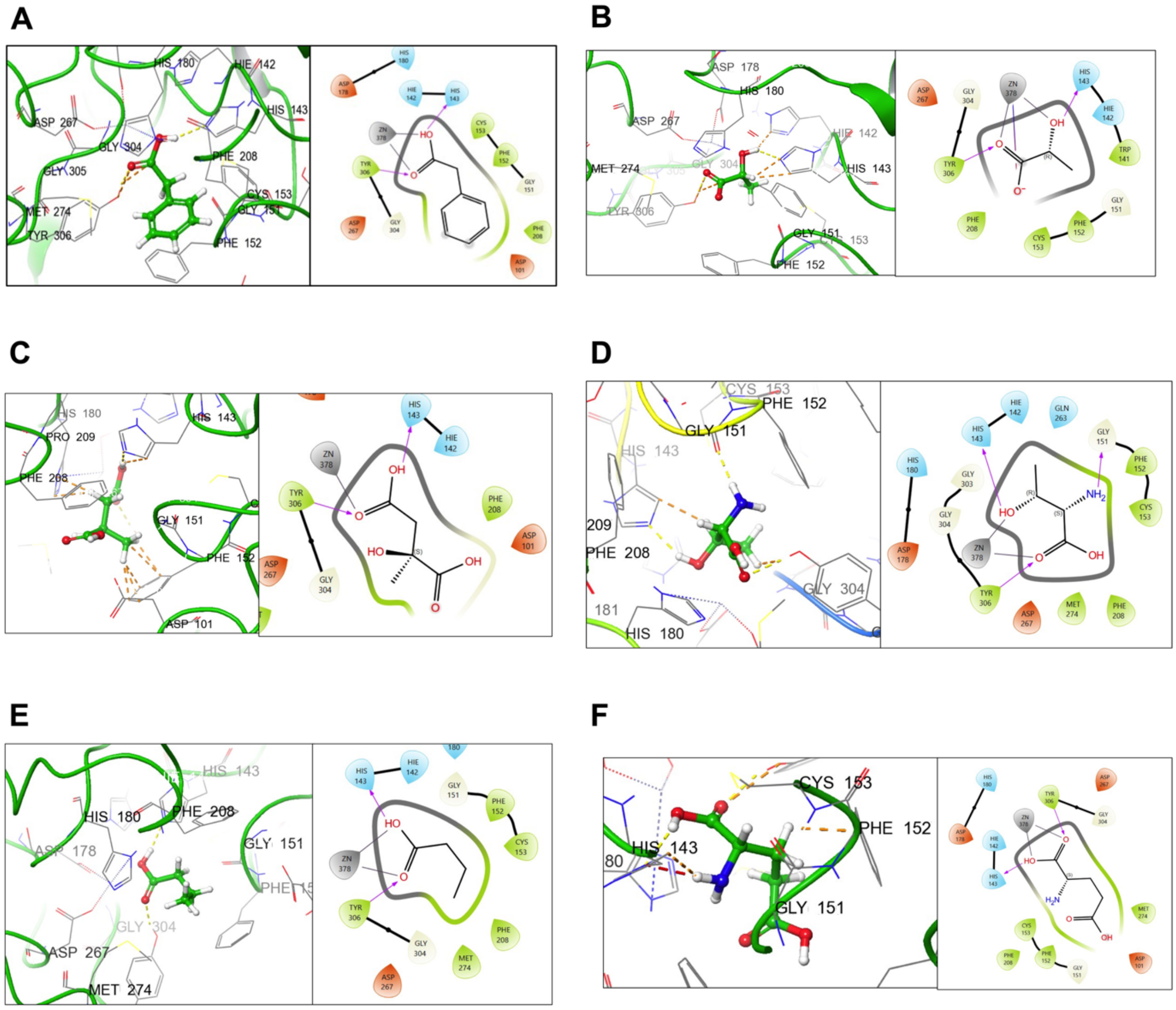

Figure S18. In-silico docking of phenylacetic acid (A), lactate (B), citramalic acid(C), threonine (D), butyric acid (D), glutamic acid (E) and with HDAC-8

5. MS suppress colorectal cancer cell migration and modulate EMT-associated markers

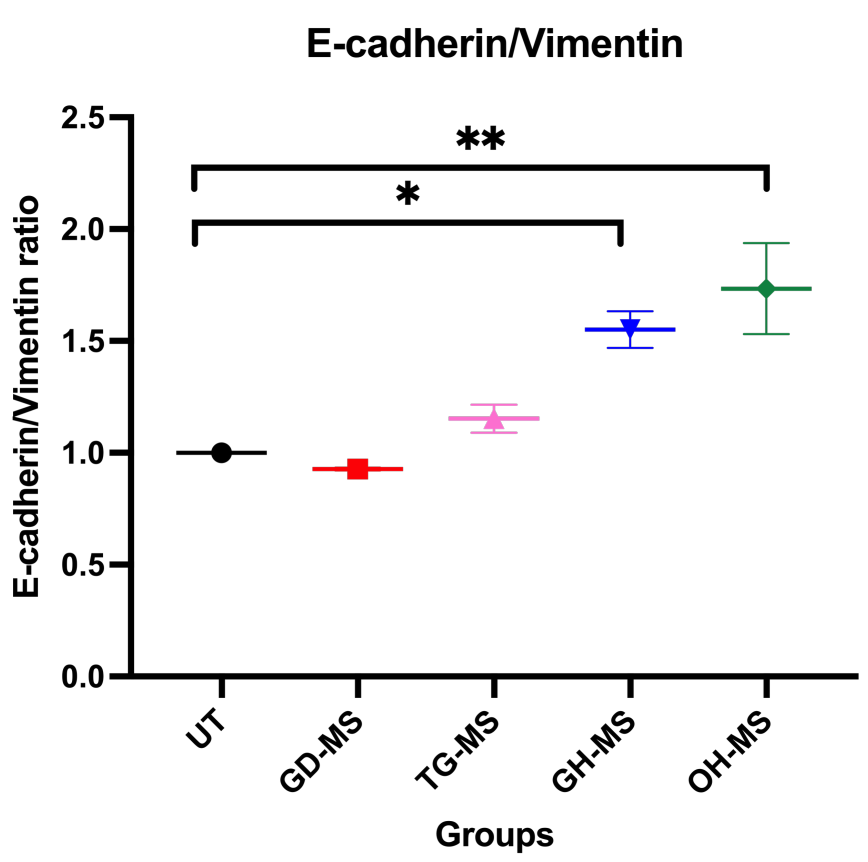

Figure S19. Densitometric analysis of E-cadherin/vimentin protein expression ratio in CT26 cells following treatment with different MS conditions. *Increased E-cadherin/vimentin ratios were observed in GH-MS and OH-MS treated cells compared with control groups.*

6. GH-MS and OH-MS modulate inflammation in CT26 cells

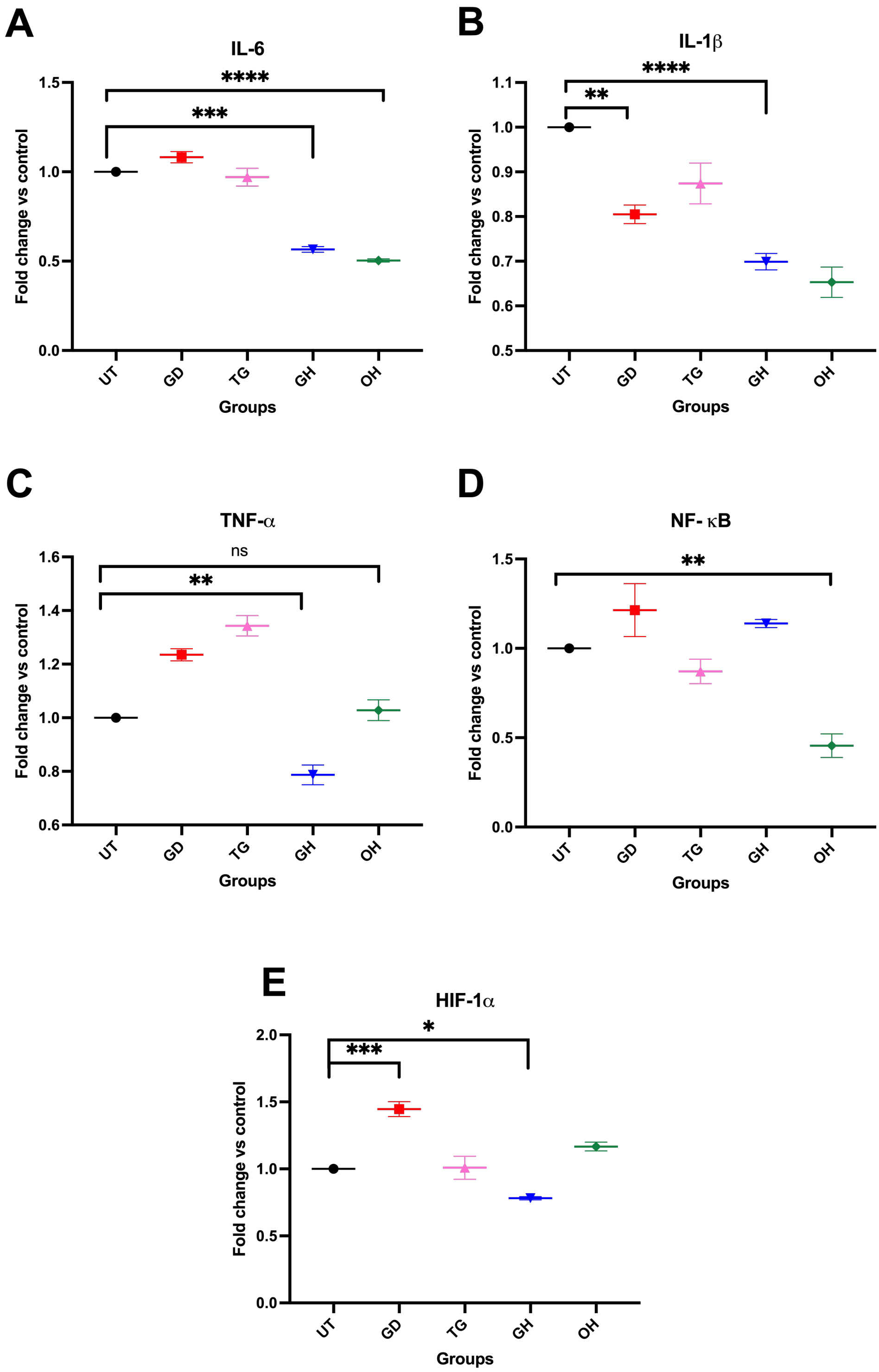

Figure S20. Densitometric analysis of (A) IL-6, (B) IL-1β, (C) TNF-α, (D) NF-κB and (E) HIF-1α expression in CT26 cells following treatment with different MS conditions.

7. **Probiotic-derived metabolites and host-microbiota conditioned media differentially modulate colorectal cancer cell viability**

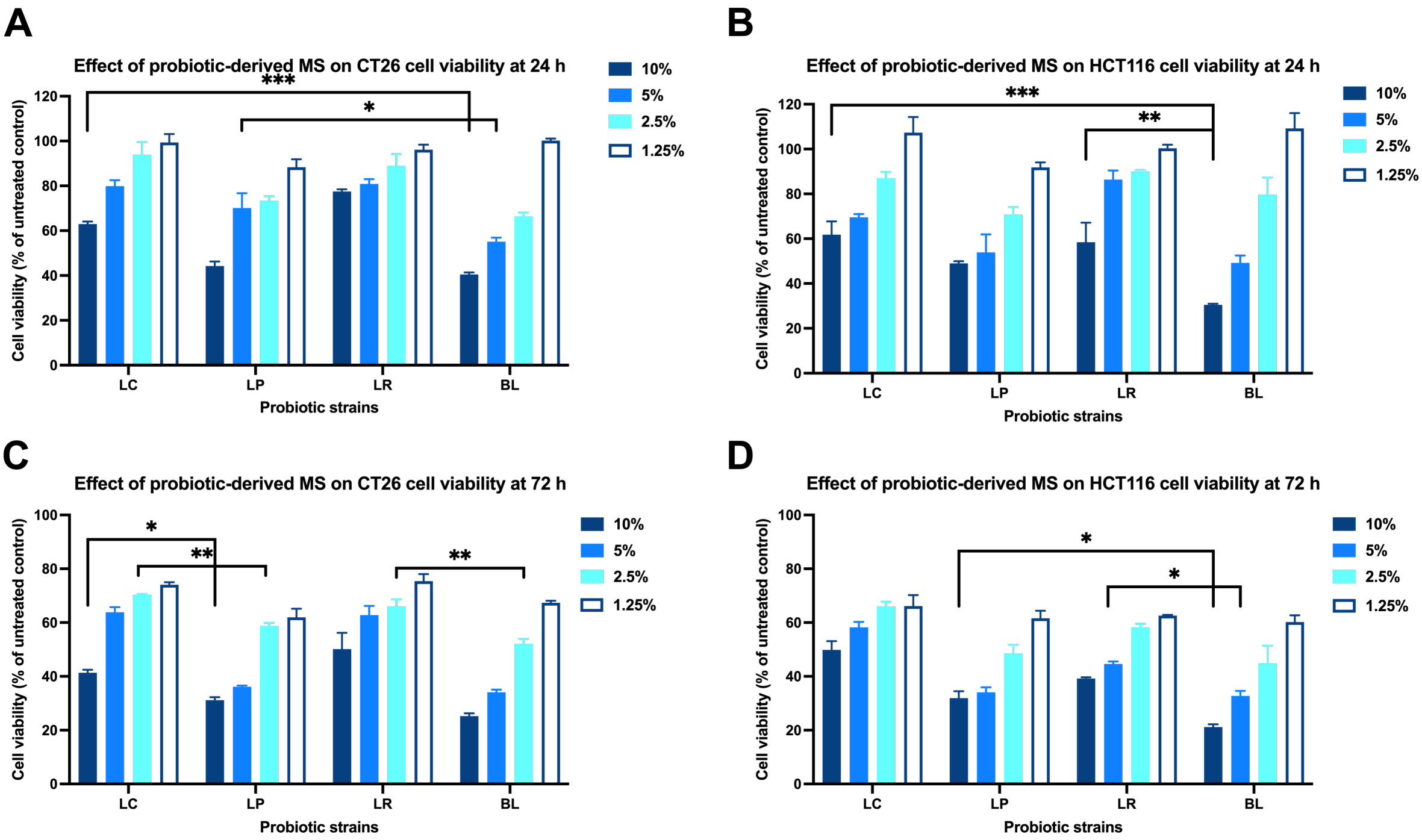

Figure S21. Cell viability analysis of murine CT26 (A,C) and human HCT116 (B,D) *colorectal cancer cells treated with MS derived from probiotic strains Lactobacillus casei strain DN-114 001 (LC), Lactobacillus plantarum strain LP-115 (LP), Lactobacillus rhamnosus strain LR32 (LR), and Bifidobacterium lactis strain BB-12 (BL) at indicated concentrations (10%, 5%, 2.5%, and 1.25%) for 24 h and 72 h respectively.*

8. **Microbiota-conditioned media induces apoptosis in colorectal cancer cells**

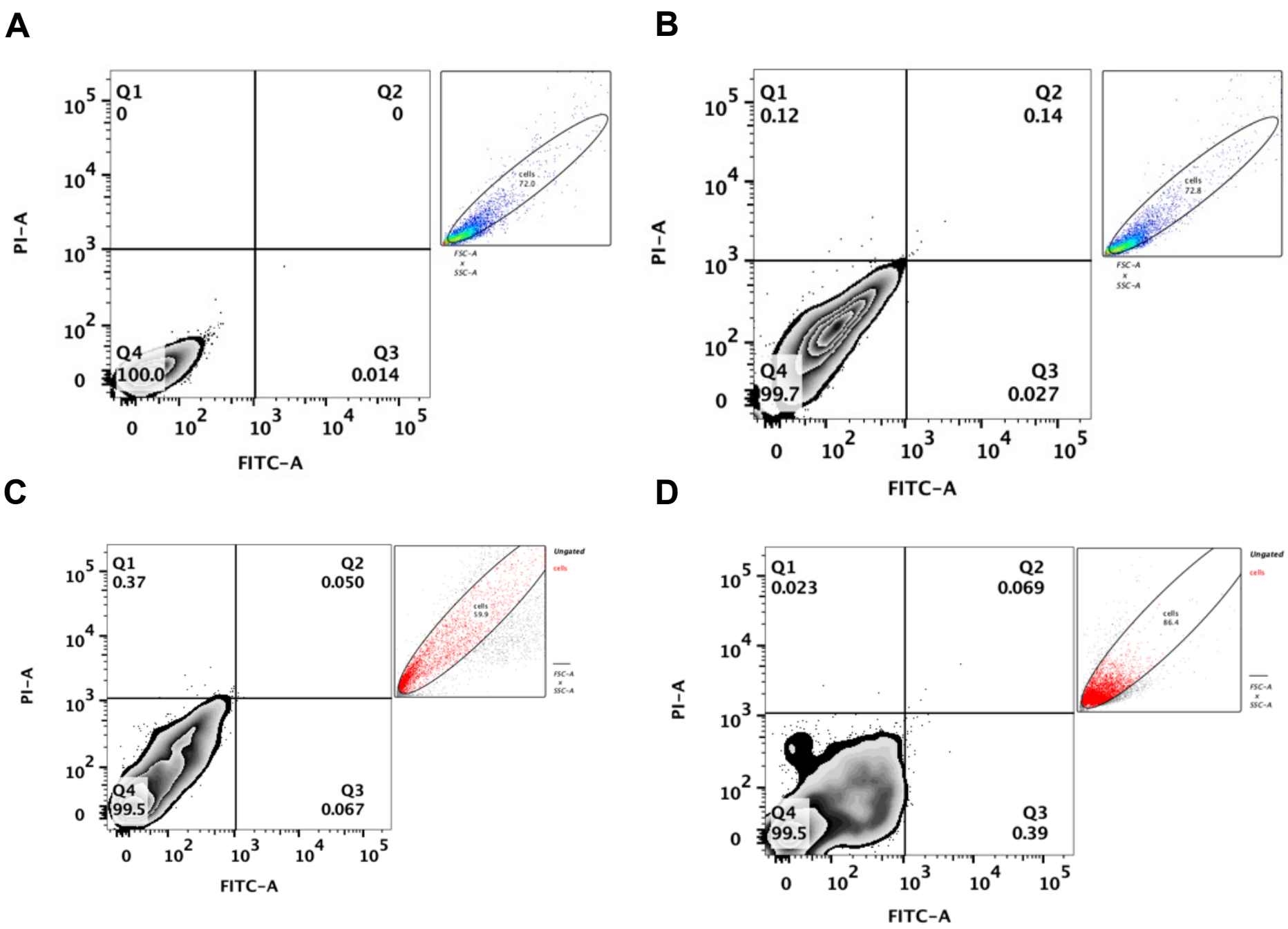

Figure S22. Control dot plots of apoptosis FACS done on CT26 and HCT116 post treatment with different MS. *(A) and (B) Unstained and stained controls of CT26 respectively; (C) and (D) Unstained and stained controls of HCT116 respectively*

9. In silico analysis of microbiota-derived metabolites with cancer-relevant target

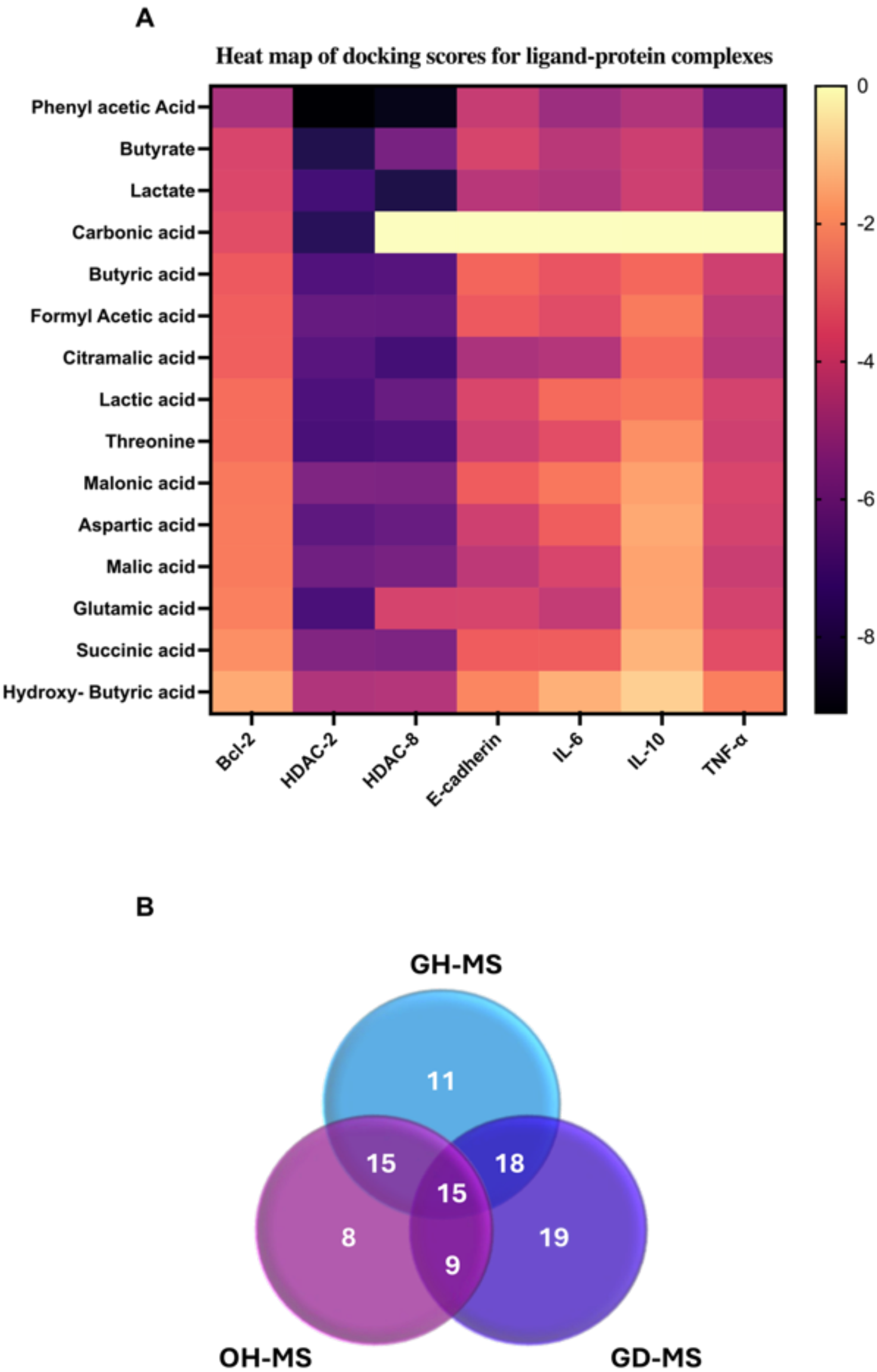

Figure S23. (A) Heatmap of docking score of in-silico docking between putative ligands identified in MS and proteins associated with cancer survival, apoptosis and inflammation. (B) Venn diagram of identified metabolites among different sources of MS.

Supplementary Table S3. Semi-quantitative comparison of putative identified metabolites across three samples identified by LC-MS.

Putative metabolite	Exact mass ESI (+/-) (with reference)	Formula	Pathway (supported by reference)	Relative intensity of identified metabolite		
				GH-MS	OH-MS	GD-MS
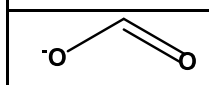 formate	186.90 [M + HCOO]$^-$ formate adduct	$HCOO^-$	Fermentation	+	++	+++
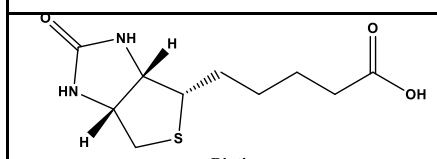 Biotin	245.10 [M+ H] +	$C_{10}H_{16}N_2O_3S$	Vitamin metabolism	+	++	+++
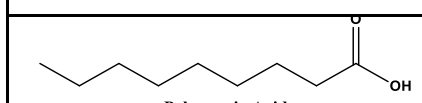 Pelargonic Acid	157.00 [M- H] $^-$	$CH_3(CH_2)_7COOH$	Fatty acid metabolism	+	++	+++
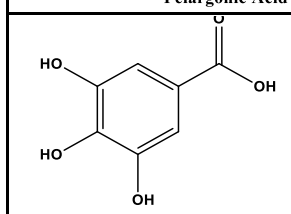 Gallic Acid	170.05 $[M+H-C_2H_2O]^+$	$C_7H_6O_5$	Phenolic metabolism	+	+	+++
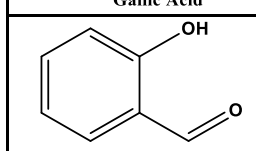 Salicylaldehyde	121.90 $[M+H]^+$	$C_7H_6O_2$	Aromatic metabolism	+	++	+++
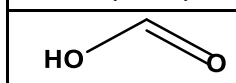 formic acid	90.07 [2M - H]$^-$	HCOOH	Fermentation	++	+	+++
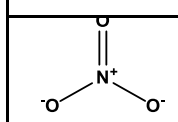 Nitrate Ion	62.20 [M - H]$^-$	NO_3^-	Dissimilatory nitrate reduction pathways	-	+	+++
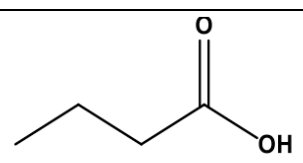 Butyric acid	88.05 [M - H]$^-$	$C_4H_8O_2$	SCFA metabolism	++	+++	+
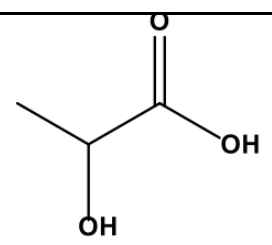 Lactic acid	90.03 [M - H]$^-$	$C_3H_6O_3$	SCFA metabolism	++	+++	+
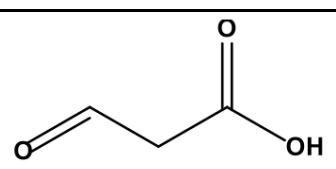 Formylacetic acid	88.02 [M - H]$^-$	$C_3H_3O_3$	Organic acid metabolism	++	+++	+
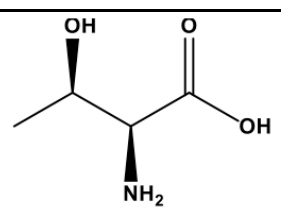 Threonine	119.06 [M - H]$^-$	$C_4H_9NO_3$	Amino acid metabolism	++	+++	+
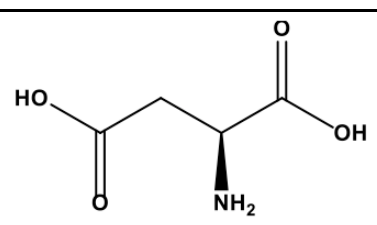 Aspartic acid	133.04 [M - H]$^-$	$C_4H_7NO_4$	Amino acid metabolism	++	+++	+
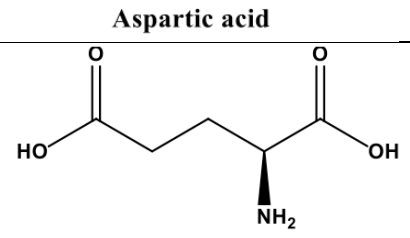 Glutamic acid	147.05 [M - H]$^-$	$C_5H_9NO_4$	Amino acid metabolism	++	+++	+
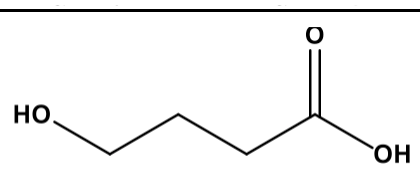 Hydroxybutyric acid	104.05 $[M+H]^+$	$C_4H_8O_3$	Energy metabolism	++	+++	+

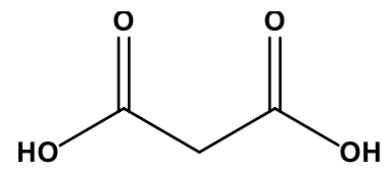 Malonic acid	104.01 $[M+Na]^+$	$C_3H_4O_4$	Dicarboxylic acid. metabolism	++	+++	+
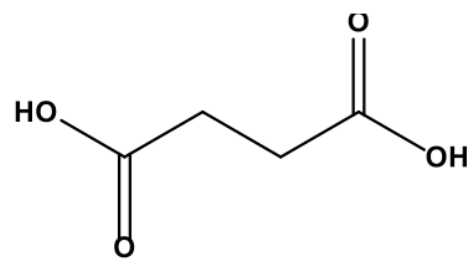 Succinic acid	118.03 $[M+Na]^+$	$C_4H_6O_4$	TCA cycle	++	+++	+
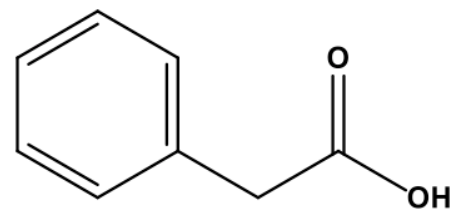 Phenylacetic acid	136.05 $[M+Na]^+$	$C_8H_8O_2$	Aromatic metabolism	+	+	+
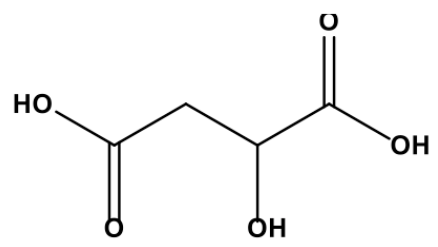 Malic acid	134.02$[M -H]^-$	$C_4H_6O_5$	TCA cycle	+	+++	+
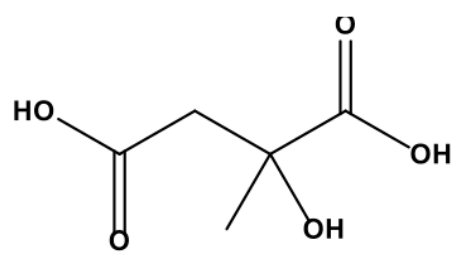 Citramalic acid	148.04$[M+Na]^+$	$C_5H_8O_5$	Microbial fermentation	++	+++	-
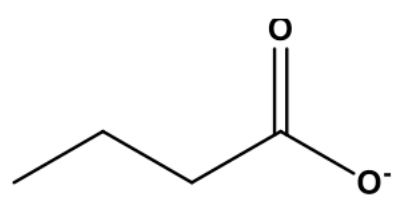 Butyrate	87.05 [M-H]-/+	$C_4H_7O_2^-$	SCFA metabolism	++	+++	+
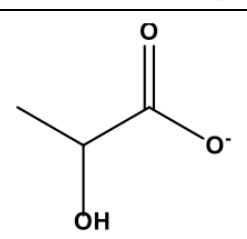 Lactate	89.02[M-H]-/+	$C_3H_5O_3^-$	SCFA metabolism	++	+++	+

Relative metabolite abundance was estimated from LC-MS peak intensities and categorized semi-quantitatively (low, moderate, high). CRC-associated samples (GD-MS) showed enrichment of lipid- and vitamin-related metabolites, whereas oral microbiota-derived samples (OH-MS) were enriched in short-chain fatty acids and amino acid-derived metabolites. Healthy gut samples (GH-MS) exhibited moderate baseline levels.

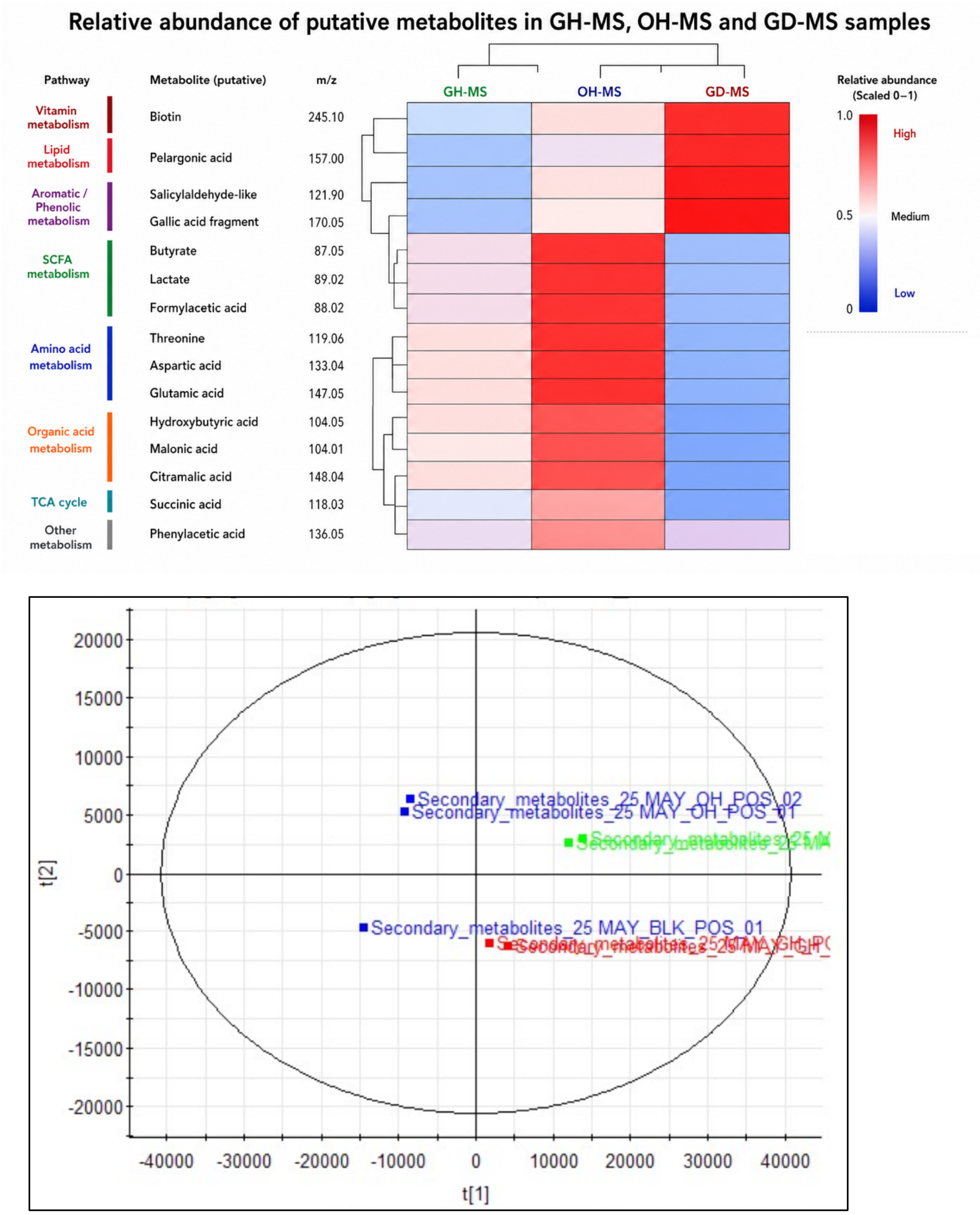

Figure S24. *Comparative LC-QTOF metabolomic profiling of microbiota-derived metabolite-enriched supernatants. Upper panel:* *Heatmap showing relative abundance of putatively annotated metabolites detected across GH-MS, OH-MS and GD-MS samples by untargeted LC-QTOF-MS analysis;* ***Lower panel:*** *Principal component analysis (PCA) score plot generated from LC-QTOF metabolite profiling demonstrating distinct clustering trends among GH-MS, OH-MS and GD-MS pooled metabolite-enriched supernatants. Blue represents OH-MS, green represents GD-MS and red represents GH-MS. Data represent pooled representative samples analyzed in exploratory untargeted metabolomic mode.*

A heatmap representing relative metabolite abundance was generated using GraphPad Prism. Semi-quantitative LC-MS peak intensities were scored to a relative scale (0-1), where 0

represents the lowest detected intensity and 1 represents the highest across samples. The heatmap was visualized using a continuous color gradient to reflect differences in metabolite abundance across GH-MS, OH-MS, and GD-MS samples. The heatmap provides a comparative overview of relative metabolite abundance derived from LC-MS peak intensities and supports the observed trends between microbiota-derived samples. The heatmap highlights distinct metabolic clustering between sample groups, with GD-MS enriched in lipid and vitamin-associated metabolites, while OH-MS shows enrichment in short-chain fatty acids and amino acid metabolism. Metabolites were organized based on their biochemical pathways and relative abundance patterns across samples. Hierarchical relationships shown in the heatmap represent similarity in abundance trends rather than strictly unsupervised clustering.
